# Physiological condition of nestling great tits (*Parus major*) declines with the date of brood initiation: a long term study of first clutches

**DOI:** 10.1038/s41598-019-46263-z

**Published:** 2019-07-08

**Authors:** Adam Kaliński, Mirosława Bańbura, Michał Glądalski, Marcin Markowski, Joanna Skwarska, Jarosław Wawrzyniak, Piotr Zieliński, Jerzy Bańbura

**Affiliations:** 1 Department of Experimental Zoology and Evolutionary Biology, Faculty of Biology and Environmental Protection, University of Łódź, Banacha 12/16, 90-237 Łódź, Poland; 2 Museum of Natural History, Faculty of Biology and Environmental Protection, University of Łódź, Kilińskiego 101, 90-011 Łódź, Poland; 3 Department of Ecology and Vertebrate Zoology, Faculty of Biology and Environmental Protection, University of Łódź, Banacha 12/16, 90-237 Łódź, Poland

**Keywords:** Ecology, Zoology

## Abstract

In seasonal environments, a temporal decline in breeding performance (e.g. clutch size, nestling condition, and fledging success) of altricial bird species is a well-known phenomenon. In this study, we present the effect of laying phenology on the physiological condition of nestling great tits (*Parus major)* in 14 consecutive breeding seasons. We used blood haemoglobin and baseline glucose concentrations as indicators of nestling physiological condition. Nestling blood haemoglobin reflects food base quality and availability during the breeding period. Blood glucose concentration can be used as a supplementary reverse index of condition, since it is negatively related to environmental quality. It might be indicative of the stress caused by unfavourable extrinsic factors, though, due to potential confounding factors such as adverse weather conditions, low food quality, or feeding interruptions, glucose levels should be used in this ecological context with caution. Great tit nestlings from earlier broods were characterised by higher mean haemoglobin concentrations, indicating a seasonal decline in food quality and availability. The blood glucose concentration displayed an opposite pattern, with nestlings from earlier broods being characterised by lower mean concentrations than those from later broods. However, very little of the variation in blood glucose concentration can be explained by the variation in laying date, which suggests that blood glucose concentration is of little importance in the context of breeding phenology. Our results show that the physiological condition of nestlings of this species decreases as the breeding season progresses, most probably due to environmental factors.

## Introduction

Proper timing of breeding is crucial in highly seasonal environments at temperate latitudes, and is primarily determined by the prevailing weather and trophic conditions^1–3^. Environmental factors influencing the onset of reproduction have been widely studied across various avian taxa^4–6^. Breeding phenology is affected by selective pressures acting on different life history stages^7^. In birds, photoperiodic cues together with the rise in temperature in spring appear to be key proximate factors activating breeding behaviour^8–11^. The match-mismatch hypothesis suggests that the timing of breeding activity evolved to match the timing of maximum food demands of nestlings with the period of peak food availability, and fitness is lower for birds that breed both earlier and later than the seasonal food peak^12,13^. However, in general, selection is thought to favour an earlier onset of reproduction, which might be beneficial for parents, enabling them to access rich food sources and proper feeding conditions for their nestlings; this is caused mainly by seasonally deteriorating external conditions, including predation or intra- and inter-specific competition^14,15^. Early-breeders then benefit from access to rich food sources, which is particularly important for resident species in temperate zones, including the European tits *Paridae*, where there might be inter- and intra-specific competition for nesting sites and good-quality territories^16^. Breeding early also allows parents to raise more than one clutch and produce more offspring per season^17,18^. The seasonal decline in reproductive performance might be caused by variation in quality between early breeding high-quality birds and late breeding low-quality birds, however, it is possible that the effects of date and parental quality are not mutually exclusive^19^.

Factors influencing the onset of reproduction are especially important in the context of relatively rapid climate changes in the last several decades, which might cause phenological disjunction^20^. Recent advancements in breeding phenology induced by global warming have been studied in many bird species, including tits^6,21–27^. However, it is not quite clear whether these shifts in phenology have positive or negative consequences for fitness, since rapid shifts to earlier breeding might also be disadvantageous if the period of maximum food requirements of nestlings mismatches the main food peak^28^. We focus here on a well-studied European model species, the great tit (*Parus major*), for which an advancement in egg laying in some areas has been observed^29,30^, whereas in other sites, no such effect has been found^6^. The occurrence of such an advancement in breeding phenology might lead to fitness consequences and, ultimately, to selection for earlier breeding. Since the great tit readily occupies nest boxes and is among the most intensively studied bird species in the world^31^, it offers opportunities to investigate the relationships between nestling condition and breeding phenology and subsequently to compare the results from various locations within its geographical range. In altricial birds, reproductive performance declines as the breeding season progresses, and it has been suggested that nestlings hatched early in the season are in better condition, which results in higher chances of survival and subsequent recruitment^14,32–34^.

For the above reasons, we should be able to link physiological condition with fitness components in nestlings. Body condition reflects a multitude of both extrinsic and intrinsic factors, which understandably attracts the attention of many researchers. There are several recent studies that have provided plenty of data on the physiological state of wild birds at various life stages, including results on offspring performance^35,36^. Physiological variation might be even more important in the temporally changing environments where European Parids breed and where decisions of when to initiate broods are of crucial importance.

Therefore, the main aim of this study was to show the relationship between laying date and the physiological condition of nestlings. We chose two physiological traits (blood haemoglobin and baseline blood glucose concentrations) to indicate the condition state of great tit nestlings. It has been previously shown that the haemoglobin concentration can be used as a robust physiological condition index in nestling great tits, since it reflects the abundance and quality of food delivered by the parents^35–37^. Blood glucose concentration is a complex trait and, in general, higher levels of blood glucose are found in individuals during the most demanding stages, i.e. migration^38^. However, it is also known that prolonged starvation or adverse environmental conditions increase baseline blood glucose levels^39,40^. Ruiz *et al*.^41^ found that rufous-collared sparrows (*Zonotrichia capensis*) had higher blood glucose levels in poorer habitats. We also analysed the applicability of blood glucose concentration as an additional index of the stress response in our study system. We showed in one of our previous studies that under bad trophic conditions, the mean blood glucose concentration was higher, which we interpreted as a result of elevated corticosteroids in response to environmental stress^42^. Therefore, we expect that rearing conditions in the environment should temporally deteriorate, which consequently should lead to a gradual decline in the physiological condition of nestlings during the course of the breeding season, i.e. we expect lower mean haemoglobin concentrations and higher mean blood glucose concentrations as the breeding season progresses. To test these predictions, in this study, we analysed haemoglobin and blood glucose concentrations in relation to laying dates using a long-term data set.

## Results

**Figure 1:**
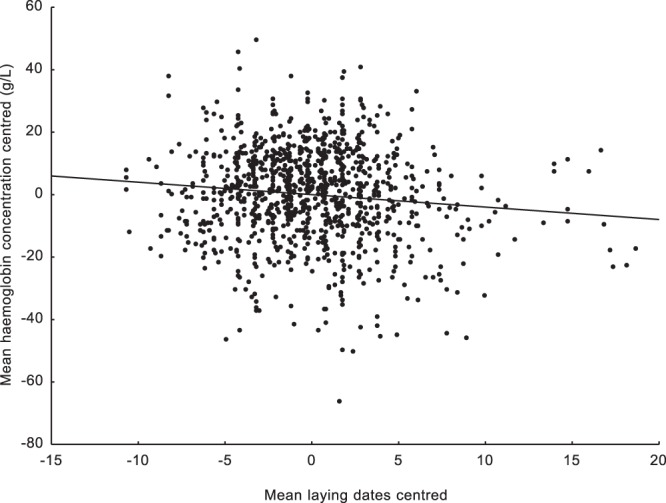
Relationship between mean centred haemoglobin concentrations and mean centred laying dates. Brood means were used (N = 1042; r = −0.108; p < 0.0001).

**Figure 2:**
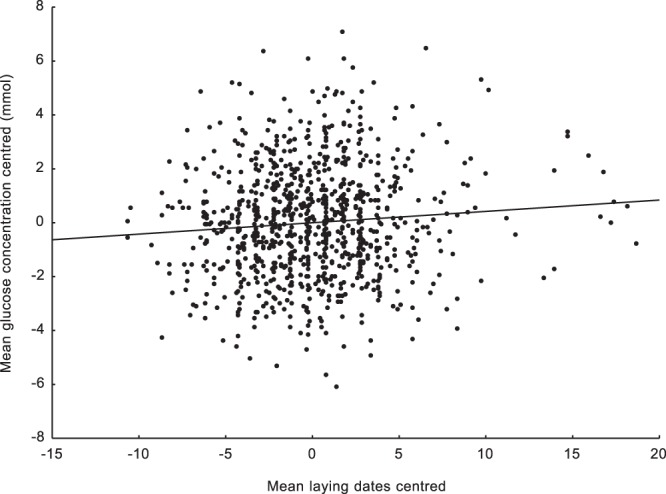
Relationship between mean centred glucose concentrations and mean centred laying dates. Brood means were used (N = 919; r = −0.088; p = 0.012).

**Figure 3:**
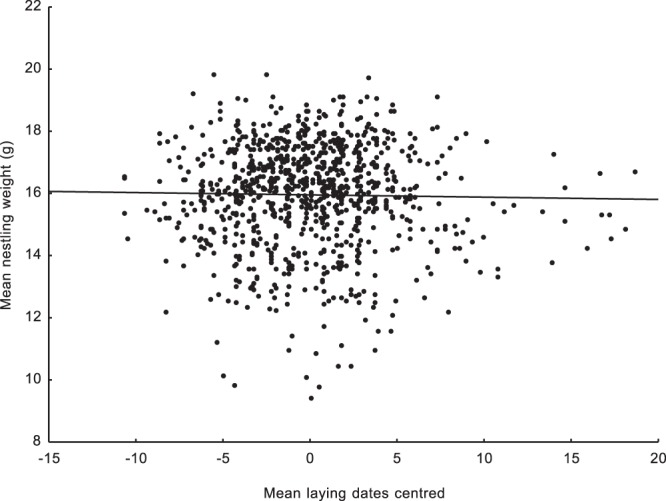
Relationship between mean body mass values and mean centred laying dates. Brood means were used (N = 944; r = −0.018; p = 0.572).

**Figure 4:**
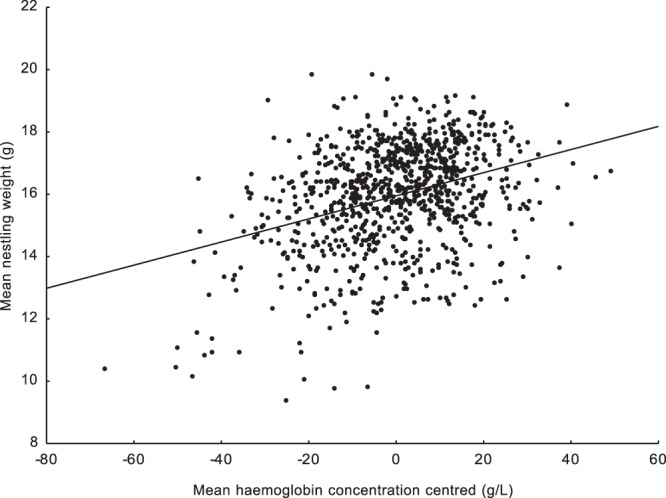
Relationship between mean body mass values and mean centred haemoglobin concentrations. Brood means were used (N = 940; r = 0.339; p < 0.0001).

**Figure 5:**
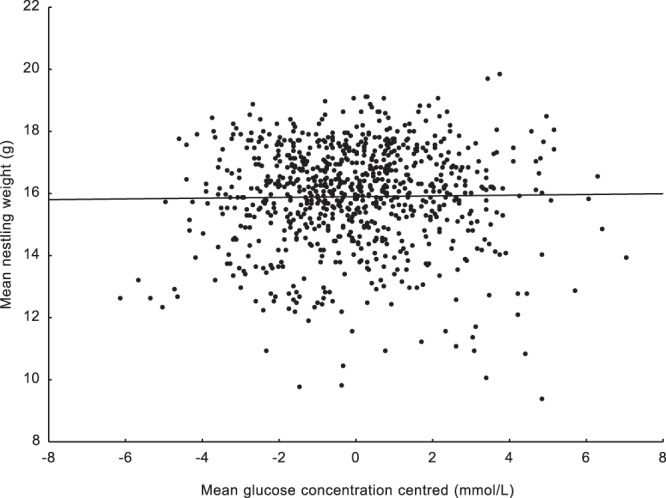
Relationship between mean body mass values and mean centred glucose concentrations. Brood means were used (N = 818; r = 0.015; p = 0.669).

We found a significant negative relationship between centred laying dates and centred haemoglobin levels. Such a result means that nestlings in earlier broods were characterised by higher mean haemoglobin levels than nestlings raised in later broods (Table 1). We were also able to show this effect using brood means of centred haemoglobin levels (N = 1042; r = −0.108; p < 0.0001; Fig. 1). This clearly shows that the mean haemoglobin concentration steadily decreases as the breeding season progresses. Moreover, the second covariant in the mixed model, nestling body mass, was positively correlated with haemoglobin concentration, which means that heavier nestlings were characterised by higher mean haemoglobin levels. The interaction between centred laying dates and nestling weight was also significant and positive (Table 1). The R^2^ value indicating how much of the variation in haemoglobin concentration is explained by the final mixed model was 0.33. An opposite pattern was found for the centred blood glucose concentration. The mean blood glucose level reached its minimum at the beginning of breeding season. The blood glucose concentration increased as the breeding season progressed (Table 1). We were also able to show this effect using brood means of centred blood glucose levels (N = 919; r = 0.088; p = 0.012; Fig. 2). The body mass covariate and the interaction between body mass and centred laying dates were nonsignificant (Table 1). The R^2^ value indicating how much of the variation in blood glucose concentration is explained by the breeding phenology in the final mixed model was 0.003. Since we used the nestling body mass as a second covariant in the models, we checked how this variable was related to laying date, haemoglobin concentration, and blood glucose concentration. The mean nestling body mass was significantly positively associated with the centred haemoglobin concentration (N = 940; r = 0.339; p < 0.001; Fig. 3). The mean nestling body mass was nonsignificantly related to both centred laying dates and centred blood glucose concentration (N = 944; r = −0.018; p = 0.572 and N = 818; r = 0.015; p = 0.669, respectively; Figs 4 and 5).

**Table 1:** Results of two linear mixed model tests for the effects of mean-centred laying dates and nestling body mass on mean-centred haemoglobin and centred glucose concentrations in the blood of nestling Great Tits. Significant effects are marked in bold. R^2^ values for the final models are given.

Trait	Effect	Est (±SE)	D_f_	F	*p*
Haemoglobin (centred)	**Intercept**	**−32.16 (±2.75)**	**1;3219.52**	**139.45**	**<0.000**
	**Laying dates (centred)**	**−1.93 (±0.67)**	**1;1476.21**	**8.316**	**0.004**
	**Nestling body mass**	**2.01 (±0.17)**	**1;3255.32**	**140.35**	**<0.000**
	Laying dates (centred)*Nestling body mass R^2^ = 0.33	**0.10 (±0.04)**	**1;1837.03**	**5.81**	**0.016**
Glucose (centred)	Intercept	−17.41 (±9.88)	1;1824.57	3.10	0.078
	Laying dates (centred)	**5.52 (±2.56)**	**1;2079.78**	**4.67**	**0.031**
	Nestling body mass	1.08 (±0.61)	1; 1841.29	3.098	0.079
	Laying dates (centred)* Nestling body mass R^2^ = 0.003	−0.31(±0.16)	1: 2097.92	3.77	0.05

## Discussion

The timing of reproduction affects various traits of avian breeding. Clutch size, fledging success, and other components of offspring condition tend to decline during the course of the breeding season^14,18,19,33,34^. In this study, we tested whether there is a relationship between the time of clutch initiation and the physiological condition indices of nestlings. In line with our expectations, breeding phenology exerts an influence on the physiological performance of nestlings. Our results show that nestlings from earlier broods were characterised by higher haemoglobin concentrations. Simultaneously, blood glucose concentrations were lower in early broods than late broods. Furthermore, heavier nestlings were characterised by higher haemoglobin concentrations. No such relationship for blood glucose concentration was found.

It is well known that many breeding parameters decrease with the progression of the nesting season, however, phenological changes in physiological parameters have not often been studied^36,43^. We suppose that the temporal reduction in mean blood haemoglobin concentrations we found results mainly from the decreasing availability of optimal food for developing chicks, since a deterioration in the caterpillar food supply during the course of the breeding season is a common feature of temperate zone habitats^44–47^. In fact, in both our study sites, the caterpillar food base decreases as the season progresses^48^. The inferior quality leading to a reduced survival probability of late hatched nestling great tits has been shown by Norte *et al*.^43^. They demonstrated a lower level of haemoglobin and haematocrit later in the season, which in general is indicative of a poor health state and anaemia resulting from poorer nutrition and a deterioration of the food base, however, it should be pointed out that the authors did not measure the total haemoglobin but the concentration of haemoglobin in haemolysate. Moreover, studies comparing first broods with second broods (which are in general raised under suboptimal feeding conditions) have revealed a lower oxygen-carrying capacity of nestlings from second broods^35,43,48–50^. Other possible and probably not mutually exclusive causes of temporal health deterioration might be related to higher levels of ecto-parasites and blood parasite loads later in the season and the accompanying blood loss of nestlings^51^. One of our experimental studies on micro- and macro-parasite loads in blue tits (*Cyanistes caeruleus*) broods showed that the impact of parasites on the health state of nestlings can be profound^52^. However, the nestlings studied by Norte *et al*.^43^ did not manifest increased white blood cell counts in response to infection, which might be due to a seasonal decline in nestling immunocompetence. Such an effect was in turn demonstrated by Dubiec & Cichoń^34,53^. They showed a seasonal decline in the nestling T-cell mediated response to phytohaemagglutinin as well as a decline in leukocyte counts, which are features directly related to the health status of an individual. Nevertheless, they also demonstrated that the seasonal variation in haematocrit was limited, which was contrary to expectations, since haematocrit is modulated to some extent by nutritional status. In fact, in one of our previous studies we showed that the haemoglobin and haematocrit of nestlings were significantly associated, however, their relationship was relatively weak, which might result from different sensitivities to ecological factors of both of these traits^54^.

We found that the blood glucose concentration increased as the season progressed in our study population. Although this relationship is significant, the effect size associated with this relationship is very small. It might arise from the fact that the blood glucose level is related to a multitude of factors, including environmental factors, that can potentially act as stressors. High levels of blood glucose might be linked to corticosterone secretion, which is stimulated by the hypothalamo-pituitary-adrenal (HPA) axis in response to chronic environmental stress of different origins^55^. Corticosteroids have the ability to increase blood glucose concentrations^56,57^. However, the underlying physiological mechanisms of how corticosterone impacts glucose levels are less clear in avian than in mammalian taxa^58^. Bearing in mind all of the confounding factors and the unclear relationship between stress and blood glucose levels, it should be pointed out that we did not measure plasma corticosterone in nestlings, therefore, we cannot draw any valid conclusion on this point. Thus, for the reasons mentioned above, the use of glucose levels as an index of physiological condition should be applied with much care, with respect to the ecological context of the research, and, preferably, as a supplemental method to complement other assays.

Since the physiological indexes of body condition are good predictors of successful fledging^36,43^ and, consequently, of subsequent survival and recruitment into the breeding population, the possible mechanisms related to the timing of reproduction should be analysed. The ascertained seasonal decline in physiological condition in nestlings suggests that early raised fledglings are generally in better condition than later raised fledglings and, therefore, their future prospects for becoming breeders in subsequent seasons are higher. It is possible that not only condition *per se* but also competition between early and late fledglings might lead to the mortality of late fledglings, since both age and prior occupancy of territories are important in establishing future dominance structure^59^. Nevertheless, the seasonal decline in fledging body condition might result from environmental circumstances or from parental quality, or a combination of both^18^. Parental quality might be of crucial importance, since it is known that the observed deterioration in quality of nestlings during the course of the breeding season results at least in part from the fact that lower quality birds tend to breed later in the season^60^. An attempt to quantitatively estimate components of reproductive success was made by Verhulst *et al*.^19^ studying great tits. They conducted a study on experimentally delayed pairs compared to control early and late breeders and assessed tentatively that 87% of the seasonal decline in breeding success was attributed to a timing effect *per se*, whereas quality differences accounted for the remaining 13%. Similar results have been shown for other bird species, e.g. the coot (*Fulica atra*), for which breeding success was causally related to the timing of breeding, with an accompanying positive correlation between male age and fledging success^61^. The above mentioned results seem to confirm the finding that parental quality is an important variable in this context, although it is often difficult to discriminate between timing and parental quality. It is possible that in our study system, environmental influences, especially deteriorating trophic conditions during the course of the breeding season, are of crucial importance. Parental quality is also likely to be important, since it is known that inexperienced first-year females, as well as senile females, breed later and less efficiently^62,63^. However, in our study system, we do not have enough pedigree data on the parents, therefore this problem remains open, and no valid conclusion can be drawn on this issue. Supposedly parental birds of higher quality that are able to provide nestlings with adequate food breed earlier. However, laying extremely early might induce costs for finding enough food for egg formation^62^. Breeding too early might also reduce the first-year post-fledging survival probability due to sudden episodes of environmental instability in early spring^64^^, own. obs.^. However, an excessive delay in laying is prone to the risk of higher post-fledging mortality^18^.

In conclusion, we want to emphasise that fledglings raised earlier in the season are in better physiological condition according to their higher haemoglobin concentrations. This study shows that in the case of great tits, early breeding is advantageous in terms of the health status of nestlings and the chances of fledging successfully, and, therefore, the prospects for future survival and recruitment. Thus, the selection pressure for breeding early in these particular great tit populations seems to be strong enough to shape phenological aspects of their life-histories. To our knowledge, there are few other studies showing negative relationships between nestling physiological condition and breeding phenology in altricial passerines in a long-term perspective. However, since there are other factors related to nestling performance during breeding season, including the quality of the parents, which were not controlled for in this study, we should treat the above results with caution. It is likely that the relatively weak correlations with accompanying high variances obtained in the models, especially in the case of blood glucose concentrations, result from these uncontrolled factors. Therefore, it should be stressed that further analyses are needed to discriminate between the effects of the timing *per se* and the quality of the parents.

## Methods

### Study sites

This study was carried out during 14 consecutive breeding seasons (2003–2016) in two sites with different habitat types: an urban park habitat of botanical and zoological gardens and a deciduous forest. The study sites are ~10 km apart and are separated by the heavily urbanised area of the city of Łódź, central Poland. The urban park site (51°45′N; 19°24′E), ca. 80 ha in total (67 ha of botanical and 13 ha of zoological gardens), belongs to a larger parkland area (~500 ha in total) in the western part of the city and is mainly of anthropogenic origin. It is characterised by a fragmented tree cover with many open spaces, intensive human traffic during spring and summer months, and a large proportion of alien tree species. The forest study site (51°50′N; 19°29′E) in the NE part of Łódź is ~130 ha in area and situated in the interior of a rich, mature, mainly deciduous and mixed forest, ~1250 ha in total, with oaks (*Quercus robur* and *Q*. *petraea*) as the dominant tree species. The forest site is characterised by a much less intense human pressure during the breeding season than the parkland site.

### Field procedures

All fieldwork for this study was performed in compliance with Polish legislation. All procedures were approved by the Local Ethical Committee and the State Office for Environment Protection. Both the study sites were supplied with standard wooden nest boxes^65^, approximately 300 in the forest site and approximately 200 in the parkland. During each breeding season, the nest boxes were inspected at least once a week from the end of March to record the basic breeding characteristics of the broods, including the date of laying the first egg. The great tit is a small, altricial passerine with a mean time to fledging of approximately three weeks in our study populations. Since nestling great tits show an increase in blood haemoglobin content during the course of their development, with the maximum being reached at the age of approximately two weeks^66^, we sampled them when the oldest chicks in a particular nest were 13–14 days old. First, we banded them with individually numbered metal rings and weighed them to the nearest 0.1 g. Subsequently, a random subsample of five (in 2003–2006) or three (in 2007–2016) nestlings was drawn from every brood for blood sampling. Blood samples were taken immediately after taking the nestling out of the nest with consecutive nestlings waiting for sampling in a non-transparent cotton bag in order to reduce a potential stress reaction (potentially caused by elevated corticosteroid levels which might affect blood glucose concentrations). The nestlings were bled sequentially from the ulnar vein using disposable hypodermic needles and two samples each of ~5 μL blood were taken. The whole procedure of sampling a particular nestling took no longer than 20–30 seconds. The first sample was transferred into HemoCue cuvettes (HemoCue AB, Angelholm, Sweden) and analysed in the field using a portable HemoCue Hb 201+ photometer to measure the haemoglobin concentration (g/L). As described by Simmons and Lill 2006^67^, this method shows slightly higher concentrations than a standard cyanmethemoglobin assay. The HemoCue photometer has been used in and recommended by many other studies on birds^67–70^. Therefore, we consider the HemoCue system to be a reliable system for examining haemoglobin concentrations in field studies of birds. Similarly, the second sample was transferred into HemoCue cuvettes and analysed in a portable HemoCue Glucose 201+ photometer (HemoCue AB, Angelholm, Sweden) to measure the blood glucose concentration (mg/dL) in peripheral blood. The haemoglobin concentration was measured over the course of the whole 14-year study period, whereas the glucose concentration was only measured from the year 2005 onwards. The nestlings were placed back in the nest immediately after sampling. All the procedures were carried out between 0900 h and 1400 h. We conducted our research at both study sites simultaneously using two teams of people to avoid confounding habitat with sampling time. During the 14 years of the study, 3,466 nestlings from 1,046 first broods were sampled, resulting in 3,440 records for haemoglobin (from 1,042 broods) and 2,832 records for glucose concentration (from 919 broods). Due to occasional technical problems with the HemoCue analysers, the haemoglobin and glucose concentrations were not measured in a few cases. All the procedures were approved by the Local Ethical Committee in Łódź (Lokalna Komisja Etyczna do Spraw Doswiadczeń na Zwierzętach w Łodzi, Uniwersytet Medyczny w Łodzi – Zakład Farmakodynamiki, ul. Muszyńskiego 1, 90–151 Łódź).

### Statistical analyses

Since the dataset analysed in this study consists of data covering a 14-year period and there was considerable variation in the breeding phenology between years and between the study sites (see our previous research^49,71^), the laying dates were centred by subtracting the mean from the individual clutch values within each study year and study site separately before model fitting; the haemoglobin and glucose concentrations were also mean-centred. Since the blood haemoglobin and glucose levels of nestlings from the same brood are not independent, the effects of breeding phenology on the haemoglobin and glucose concentrations in broods of great tits were analysed using linear mixed models, with brood identity, year, and study site included as random effects to control for clustering; the degrees of freedom were estimated using the Satterthwaite method^72^. The effects of breeding phenology on haemoglobin and glucose concentrations were modelled by fitting two separate models, including centred haemoglobin and centred glucose levels as dependent variables and centred laying dates as covariates in each of them^73^. We also expected that blood haemoglobin and blood glucose levels might co-vary with body mass, and therefore, we added nestling body mass to each model. The mixed models were computed using IBM SPSS v.22 software^74^.

## Supplementary information

Supplementary Dataset 1

## References

[1] Drent RH, Daan S (1980). The prudent parent: energetic adjustments in avian breeding. Ardea..

[2] Newton, I. *Population Limitation in Birds*. (Academic Press, 1998).

[3] Pakanen Veli-Matti, Orell Markku, Vatka Emma, Rytkönen Seppo, Broggi Juli (2016). Different Ultimate Factors Define Timing of Breeding in Two Related Species. PLOS ONE.

[4] Post E, Forchhammer MC, Stenseth NC, Callaghan TV (2001). The timing of life history events in a changing climate. P. Roy. Soc. Lond. B.

[5] Crick HQP (2004). The impact of climate change on birds. Ibis..

[6] Goodenough AE, Hart AG, Stafford R, Elliot SL (2011). Contrasting temporal changes in lay-date distributions in co-occurring populations of Blue Tits Cyanistes caeruleus and Great Tits Parus major. Bird Study..

[7] Perrins CM (1970). The timing of birds’ breeding season. Ibis..

[8] Buse A, Dury SJ, Woodburn RJW, Perrins CM, Good JEG (1999). Effects of elevated temperature on multi-species interactions: the case of Pedunculate Oak, Winter Moth and Tits. Funct. Ecol..

[9] Lambrechts MM, Perret P (2000). A long photoperiod overrides non‐photoperiodic factors in blue tits’ timing of reproduction. P. Roy. Soc. Lond. B.

[10] Nilsson JA, H. Källander H (2006). Leafing phenology and timing of egg laying in great tits Parus major and blue tits P. Caeruleus. J. Avi. Biol..

[11] Schaper SV, Rueda C, Sharp PJ, Dawson A, Visser M (2011). Spring phenology does not affect timing of reproduction in the great tit (Parus major). J. Exp. Zool..

[12] Durant JM, Hjerman DØ, Ottersen G, Stenseth NC (2007). Climate and the match or mismatch between predator requirements and resource availability. Clim. Res..

[13] Dunn PO, Winkler DW, Whittingham LA, Hannon SJ, Robertson RJ (2011). A test of the mismatch hypothesis: how is timing of reproduction related to food abundance in aerial insectivore?. Ecology..

[14] Verboven N, Visser ME (1998). Seasonal variation in local recruitment of great tits: the importance of being early. Oikos..

[15] Verhulst S, Nilsson J-Å (2008). The timing of birds’ breeding seasons: a review of experiments that manipulated timing of breeding. Philos. T. Roy. Soc. B.

[16] Dhondt AA, Eyckerman R (1980). Competition and the regulation of numbers in Great and Blue tit. Ardea..

[17] Hochachka W (1990). Seasonal decline in reproductive performance of Song Sparrows. Ecology..

[18] Naef-Daenzer B, Widmer F, Nuber M (2001). Differential post-fledging survival of great and coal tits in relation to their condition and fledging date. J. Anim. Ecol..

[19] Verhulst S, van Balen JH, Tinbergen JM (1995). Seasonal decline in reproductive success of the great tit: variation in time or quality?. Ecology..

[20] Visser ME, Both C (2005). Shifts in phenology due to global climate change: the need for a yardstick. P. Roy. Soc. Lond. B.

[21] Parmesan C, Yohe G (2003). A globally coherent fingerprint of climate change impacts across natural systems. Nature..

[22] Wesołowski T, Cholewa M (2009). Climate variation and bird breeding seasons in a primeval temperate forest. Clim. Res..

[23] Goodenough AE (2015). Quantifying the robustness of first arrival dates as a measure of avian migratory phenology. Ibis..

[24] Lof ME, Reed TE, McNamara JM, Visser ME (2012). Timing in a fluctuating environment: environmental variability and asymetric fitness curves can lead to adaptively mismatched avian reproduction. P. Roy. Soc. Lond. B.

[25] Hinks AE (2015). Scale-dependent phenological synchrony between songbirds and their caterpillar food source. Am. Nat..

[26] Glądalski M (2016). Effects of extreme thermal conditions on plasticity in breeding phenology and double-broodedness of Great Tits and Blue Tits in central Poland in 2013 and 2014. Int. J. Biomet..

[27] Simmonds EG, Sheldon BC, Coulson T, Cole E (2017). Incubation behavior adjustments, driven by ambient temperature variation, improve synchrony between hatch dates and caterpillar peak in a wild bird population. Ecol. Evol..

[28] Visser ME, Holleman JM, Gienapp P (2006). Shifts in caterpillar biomass phenology due to climate change and its impact on the breeding biology of an insectivorous bird. Oecologia..

[29] McCleery RH, Perrins CM (1998). Temperature and egg laying trends. Nature..

[30] Matthysen E, Adriaensen F, Dhondt AA (2011). Multiple responses to increasing spring temperatures in the breeding cycle of Blue and Great Tits (Cyanistes caeruleus, Parus major). Global Change Biol..

[31] Gosler, A. *The Great Tit*. (Hamlyn, London 1993).

[32] Garnett MC (1981). Body size, its heritability and influence on juvenile survival among great tits, *Parus major*. Ibis..

[33] Barba E, Gil-Delgado JA, Monros JS (1995). Costs of being late: consequences of delaying great tit Parus major first clutches. J. Anim. Ecol..

[34] Dubiec A, Cichoń M (2001). Seasonal decline in health status of Great Tit (Parus major) nestlings. Can. J. Zool..

[35] O’Brien EL, Morrison BL, Johnson LS (2001). Assessing the effects of haematophagous ectoparasites on the health of nestling birds: haematocrit vs haemoglobin levels in House Wrens parasitized by blow fly larvae. J. Avi. Biol..

[36] Kaliński A (2015). Long-term variation in hemoglobin concentration in nestling great tits Parus major. Comp. Biochem. Phys. A.

[37] Minias P (2015). The use of haemoglobin concentrations to assess physiological condition in birds: a review. Conserv. Physiol..

[38] Jenni-Eiermann S, Jenni L (1994). Plasma metabolite levels predict individual body-mass changes in a small long-distance migrant, the Garden Warbler. Auk..

[39] Rodriguez P, Tortosa FS, Villafuerte R (2005). The effects of fasting and refeeding on biochemical parameters in the red-legged partridge (*Alectoris rufa*). Comp. Biochem. Phys. A Mol. Integr. Physiol..

[40] Montoya B, Briga M, Jimeno B, Moonen S, Verhulst T (2018). Baseline glucose level is an individual trait that is negatively associated with lifespan and increases due to adverse environmental conditions during development and adulthood. J. Comp. Physiol. B..

[41] Ruiz G, Rosenmann M, Novoa FF, Sabat P (2002). Hematological parameters and stress index in rufous-collared sparrows dwelling in urban environments. Condor..

[42] Kaliński A (2014). Landscape patterns of variation in blood glucose concentration of nestling blue tits (Cyanistes caeruleus). Landscape Ecol..

[43] Norte AC, Ramos JA, Araujo PM, Sousa JP, Sheldon BC (2008). Health-state variables and enzymatic biomarkers as survival predictors in nestling great tits (Parus major): effect of environmental conditions. Auk..

[44] Keller LF, van Noordwijk AJ (1993). A method to isolate environmental effects on nestling growth, illustrated with examples from the great tit (Parus major). Funct. Ecol..

[45] van Noordwijk AJ, McCleery RH, Perrins CM (1995). Selection for the timing of great tit breeding in relation to caterpillar growth and temperature. J. Anim. Ecol..

[46] Verboven N, Verhulst S (1996). Seasonal variation in the incidence of double broods: the date hypothesis fits better than the quality hypothesis. J. Anim. Ecol..

[47] Naef-Daenzer B, Keller L (1999). The foraging performance of great and blue tits (*Parus major* and *P*. *caeruleus*) in relation to caterpillar development, and its consequences for nestling growth and fledging weight. J. Anim. Ecol..

[48] Marciniak B, Nadolski J, Nowakowska M, Loga B, Bańbura J (2007). Habitat and annual variation in arthropod abundance affects Blue Tit *Cyanistes caeruleus* reproduction. Acta Ornithol..

[49] Kaliński A (2009). Haemoglobin concentration and body condition of nestling Great Tits *Parus major*: a comparison of first and second broods in two contrasting seasons. Ibis..

[50] Cornell A, Gibson KF, Williams TD (2017). Physiological maturity at a critical life-history transition and flight ability at fledging. Funct. Ecol..

[51] Whittow G. C. (eds) *Sturkie’s Avian Physiology*. San Diego: Academic Press (2000).

[52] Słomczyński R (2006). Effects of experimental reduction in nest micro-parasite and macro-parasite loads on nestling hemoglobin level in tits *Parus caeruleus*. Acta Oecol..

[53] Dubiec A, Cichoń M (2005). Seasonal decline in nestling cellular immunocompetence results from environmental factors—an experimental study. Can. J. Zool..

[54] Kaliński A (2011). Weak correlation between haemoglobin concentration and haematocrit of nestlings Great Tits *Parus major* and Blue tits *P*. *caeruleus*. Ornis Fennica.

[55] Cockrem JF (2007). Stress, corticosterone responses and avian personalities. J. Ornithol. 148 supplement.

[56] Sapolsky RM, Romero, Munck AU (2000). How do glucocorticoids influence stress responses? Integrating permissive, suppressive, stimulatory, and preparative actions. Endocr. Revs..

[57] Remage-Healey L, Romero ML (2001). Corticosterone and insulin interact to regulate glucose and triglyceride levels during stress in a bird. Am. J. Physiol. Regul. Integr. Comp. Physiol..

[58] Braun EJ, Sweazea K (2008). Glucose regulation in birds. Comp. Biochem. Physiol. B..

[59] Sandell M, Smith HG (1991). Dominance, prior occupancy, and winter residency in Great Tit (Parus major). Behav. Ecol. Sociobiol..

[60] Cornell A, Williams TD (2016). Individual quality and double-brooding in a highly synchronous songbird population. Auk..

[61] Brinkhof MWG, Cave F, Hage J, Verhulst S (1993). Timing of reproduction and fledging success in the Coot Fulica atra: evidence for a causal relationship. J. Anim. Ecol..

[62] Dhondt AA (1985). Do old great tits forego breeding?. Auk..

[63] Dhondt AA (1989). The effect of old age on the reproduction of Great and Blue Tit. Ibis..

[64] Rodriguez S, van Nordvijk AJ, Alvarez E, Barba E (2016). A recipe for postfledging survival in great tits *Parus major*: be large and be early (but not too much). Ecol. Evol..

[65] Lambrechts MM (2010). The design of artificial nestboxes for the study of secondary hole-nesting birds: a review of methodological inconsistencies and potential biases. Acta Ornithol..

[66] Kostelecka-Myrcha A, Pinowski J, Tomek T (1973). Changes in the hematological values during the nestling period of the Great Tit (Parus major L.). Bull. Pol. Acad. Sci..

[67] Simmons P, Lill A (2006). Development of parameters influencing blood oxygen carrying capacity in the Welcome Swallow and Fairy Martin. Comp. Biochem. Physiol. A..

[68] Harter, T. S., Reichert, M., Brauner, C. J. & Milsom, W. K. Validation of the i-STAT and HemoCue systems for the analysis of blood parameters in the bar-headed goose, *Anser indicus*. *Conserv*. *Physiol*. **3**, 10.1093/conphys/cov21 (2015). 10.1093/conphys/cov021 PMC4778437 27293706

[69] Burness GP, Ydenberg RC, Hochachka PW (2001). Physiological and biochemical correlates of brood size and energy expenditure in tree swallows. J. Exp. Biol..

[70] Minias P, Kaczmarek K (2013). Concentrations of plasma metabolites as predictors of nestling condition in the Great Cormorant Phalacrocorax carbo sinensis. Ornis Fennica..

[71] Wawrzyniak J (2015). Long-term variation in laying date and clutch size of the great tit Parus major in central Poland: a comparison between urban parkland and deciduous forest. Ardeola..

[72] Heck, R. H., S. L. Thomas & Tabata L. N. *Multilevel and Longitudinal Modeling with IBM SPSS*. (New York: Routledge, 2010).

[73] Crawley, M. J. Statistical computing: an introduction to data analysis using S-Plus. (Chichester: Wiley, 2002).

[74] IBM SPSS Statistics 22. SPSS for Windows, Release 22.0, IBM Corporation (2013).

