# Supplementary material for: Physiological condition of nestling great tits (Parus major) declines with the date of brood initiation: a long term study of first clutches
Source: Sci Rep. 2019 Jul 8;9:9843. doi: 10.1038/s41598-019-46263-z (PMC6614424; doi:10.1038/s41598-019-46263-z)
Supplement: Supplementary file 1 — Supplementary Dataset 1 [file 41598_2019_46263_MOESM1_ESM.pdf]

Physiological condition of nestling great tits (*Parus major*) declines with the date of brood initiation: a long term study of first clutches

Adam Kaliński[1*], Mirosława Bańbura[2], Michał Glądalski[1], Marcin Markowski[1], Joanna Skwarska[1], Jarosław Wawrzyniak[1], Piotr Zieliński[3], Jerzy Bańbura[1]

[1]Department of Experimental Zoology and Evolutionary Biology, Faculty of Biology and Environmental Protection, University of Łódź, Banacha 12/16, 90-237 Łódź, Poland
[2]Museum of Natural History, Faculty of Biology and Environmental Protection, University of Łódź, Kilińskiego 101, 90-011 Łódź, Poland
[3]Department of Ecology and Vertebrate Zoology, Faculty of Biology and Environmental Protection, University of Łódź, Banacha 12/16, 90-237 Łódź, Poland
* Corresponding author: adam.kalinski@biol.uni.lodz.pl

Year	Site	Brood_id	date_centered	hb_centered	glu_centered	Body_mass
**2003**	Forest	**3_03L1_**	-4.631578947	**-26.4526316**		17
**2003**	Forest	**3_03L1_**	-4.631578947	**-24.4526316**		17.5
**2003**	Forest	**3_03L1_**	-4.631578947	**-24.4526316**		17.5
**2003**	Forest	**3_03L1_**	-4.631578947	**-11.4526316**		17
**2003**	Forest	**3_03L1_**	-4.631578947	**-1.45263158**		17.5
**2003**	Forest	**7_03L1_**	1.368421053	**-43.4526316**		18.5
**2003**	Forest	**7_03L1_**	1.368421053	**-37.4526316**		17.5
**2003**	Forest	**7_03L1_**	1.368421053	**-36.4526316**		20
**2003**	Forest	**7_03L1_**	1.368421053	**-17.4526316**		19.5
**2003**	Forest	**7_03L1_**	1.368421053	**-10.4526316**		19.5
**2003**	Forest	**18_03L1_**	-3.631578947	**-16.4526316**		17.5
**2003**	Forest	**18_03L1_**	-3.631578947	**-1.45263158**		17.5
**2003**	Forest	**18_03L1_**	-3.631578947	**3.54736842**		18
**2003**	Forest	**18_03L1_**	-3.631578947	**6.54736842**		18
**2003**	Forest	**18_03L1_**	-3.631578947	**7.54736842**		18
**2003**	Forest	**27_03L1_**	-7.631578947	**2.54736842**		17.5
**2003**	Forest	**27_03L1_**	-7.631578947	**8.54736842**		19.5
**2003**	Forest	**27_03L1_**	-7.631578947	**13.5473684**		17.5
**2003**	Forest	**27_03L1_**	-7.631578947	**25.5473684**		18
**2003**	Forest	**27_03L1_**	-7.631578947	**28.5473684**		16.5
**2003**	Forest	**44_03L1_**	4.368421053	**-19.4526316**		18
**2003**	Forest	**44_03L1_**	4.368421053	**-14.4526316**		16.5
**2003**	Forest	**44_03L1_**	4.368421053	**-10.4526316**		18.5
**2003**	Forest	**44_03L1_**	4.368421053	**-0.45263158**		18.5
**2003**	Forest	**44_03L1_**	4.368421053	**1.54736842**		18.5
**2003**	Forest	**48_03L1_**	4.368421053	**-1.45263158**		17.5
**2003**	Forest	**48_03L1_**	4.368421053	**6.54736842**		18.5
**2003**	Forest	**48_03L1_**	4.368421053	**9.54736842**		17
**2003**	Forest	**48_03L1_**	4.368421053	**14.5473684**		18
**2003**	Forest	**48_03L1_**	4.368421053	**20.5473684**		17
**2003**	Forest	**68_03L1_**	7.368421053	**-0.45263158**		19.5
**2003**	Forest	**68_03L1_**	7.368421053	**3.54736842**		17
**2003**	Forest	**68_03L1_**	7.368421053	**3.54736842**		17.5
**2003**	Forest	**68_03L1_**	7.368421053	**9.54736842**		18
**2003**	Forest	**68_03L1_**	7.368421053	**22.5473684**		18.5
**2003**	Forest	**87_03L1_**	3.368421053	**-19.4526316**		20.5
**2003**	Forest	**87_03L1_**	3.368421053	**-10.4526316**		18
**2003**	Forest	**87_03L1_**	3.368421053	**-8.45263158**		19
**2003**	Forest	**87_03L1_**	3.368421053	**-7.45263158**		19
**2003**	Forest	**87_03L1_**	3.368421053	**-0.45263158**		19
**2003**	Forest	**91_03L1_**	-6.631578947	**7.54736842**		17
**2003**	Forest	**91_03L1_**	-6.631578947	**11.5473684**		18
**2003**	Forest	**91_03L1_**	-6.631578947	**11.5473684**		19.5
**2003**	Forest	**91_03L1_**	-6.631578947	**14.5473684**		19
**2003**	Forest	**91_03L1_**	-6.631578947	**22.5473684**		17
**2003**	Forest	**101_03L1_**	1.368421053	**-6.45263158**		18
**2003**	Forest	**101_03L1_**	1.368421053	**-5.45263158**		17.5
**2003**	Forest	**101_03L1_**	1.368421053	**0.54736842**		19
**2003**	Forest	**101_03L1_**	1.368421053	**0.54736842**		17
**2003**	Forest	**101_03L1_**	1.368421053	**0.54736842**		17
**2003**	Forest	**104_03L1_**	3.368421053	**-23.4526316**		17.5
**2003**	Forest	**104_03L1_**	3.368421053	**-14.4526316**		18
**2003**	Forest	**104_03L1_**	3.368421053	**-13.4526316**		17.5

**2003**	Forest	**104_03L1_**	3.368421053	**-8.45263158**	20
**2003**	Forest	**104_03L1_**	3.368421053	**-3.45263158**	17.5
**2003**	Forest	**105_03L1_**	-8.631578947	**-26.4526316**	17.5
**2003**	Forest	**105_03L1_**	-8.631578947	**-25.4526316**	18
**2003**	Forest	**105_03L1_**	-8.631578947	**-20.4526316**	19.5
**2003**	Forest	**105_03L1_**	-8.631578947	**-14.4526316**	17.5
**2003**	Forest	**105_03L1_**	-8.631578947	**-12.4526316**	17
**2003**	Forest	**110_03L1_**	-1.631578947	**-0.45263158**	18.5
**2003**	Forest	**110_03L1_**	-1.631578947	**1.54736842**	18
**2003**	Forest	**110_03L1_**	-1.631578947	**6.54736842**	19.5
**2003**	Forest	**110_03L1_**	-1.631578947	**9.54736842**	18.5
**2003**	Forest	**110_03L1_**	-1.631578947	**11.5473684**	19
**2003**	Forest	**128_03L1_**	1.368421053	**5.54736842**	17.5
**2003**	Forest	**128_03L1_**	1.368421053	**11.5473684**	19
**2003**	Forest	**128_03L1_**	1.368421053	**16.5473684**	18
**2003**	Forest	**128_03L1_**	1.368421053	**18.5473684**	18
**2003**	Forest	**128_03L1_**	1.368421053	**23.5473684**	17
**2003**	Forest	**158_03L1_**	8.368421053	**-17.4526316**	14
**2003**	Forest	**158_03L1_**	8.368421053	**-8.45263158**	13.5
**2003**	Forest	**158_03L1_**	8.368421053	**-0.45263158**	16
**2003**	Forest	**158_03L1_**	8.368421053	**0.54736842**	13.5
**2003**	Forest	**158_03L1_**	8.368421053	**2.54736842**	14
**2003**	Forest	**167_03L1_**	-5.631578947	**14.5473684**	18.5
**2003**	Forest	**167_03L1_**	-5.631578947	**21.5473684**	19
**2003**	Forest	**167_03L1_**	-5.631578947	**24.5473684**	19
**2003**	Forest	**167_03L1_**	-5.631578947	**31.5473684**	19
**2003**	Forest	**167_03L1_**	-5.631578947	**35.5473684**	19
**2003**	Forest	**168_03L1_**	-3.631578947	**-0.45263158**	20
**2003**	Forest	**168_03L1_**	-3.631578947	**1.54736842**	19
**2003**	Forest	**168_03L1_**	-3.631578947	**3.54736842**	18.5
**2003**	Forest	**168_03L1_**	-3.631578947	**4.54736842**	18.5
**2003**	Forest	**168_03L1_**	-3.631578947	**14.5473684**	19
**2003**	Forest	**176_03L1_**	7.368421053	**-6.45263158**	20.5
**2003**	Forest	**176_03L1_**	7.368421053	**-0.45263158**	19.5
**2003**	Forest	**176_03L1_**	7.368421053	**2.54736842**	18.5
**2003**	Forest	**176_03L1_**	7.368421053	**2.54736842**	19.5
**2003**	Forest	**176_03L1_**	7.368421053	**8.54736842**	17.5
**2003**	Forest	**193_03L1_**	-0.631578947	**-8.45263158**	18.5
**2003**	Forest	**193_03L1_**	-0.631578947	**-0.45263158**	18
**2003**	Forest	**193_03L1_**	-0.631578947	**3.54736842**	17
**2003**	Forest	**193_03L1_**	-0.631578947	**11.5473684**	18.5
**2003**	Forest	**193_03L1_**	-0.631578947	**15.5473684**	17.5
**2003**	Park	**2_03b_**	-2.958579882	**0.73372781**	14.5
**2003**	Park	**2_03b_**	-2.958579882	**2.73372781**	17
**2003**	Park	**2_03b_**	-2.958579882	**5.73372781**	15
**2003**	Park	**2_03b_**	-2.958579882	**5.73372781**	15
**2003**	Park	**2_03b_**	-2.958579882	**11.7337278**	16
**2003**	Park	**4_03b_**	-1.958579882	**-11.2662722**	15
**2003**	Park	**4_03b_**	-1.958579882	**-4.26627219**	15.5
**2003**	Park	**4_03b_**	-1.958579882	**-4.26627219**	16
**2003**	Park	**4_03b_**	-1.958579882	**6.73372781**	15.5
**2003**	Park	**4_03b_**	-1.958579882	**11.7337278**	16.5
**2003**	Park	**10_03b_**	-1.958579882	**-14.2662722**	17.5
**2003**	Park	**10_03b_**	-1.958579882	**-9.26627219**	17.5

**2003**	Park	**10_03b_**	-1.958579882	**-7.26627219**	16.5
**2003**	Park	**10_03b_**	-1.958579882	**10.7337278**	18
**2003**	Park	**13_03b_**	-0.958579882	**-18.2662722**	14
**2003**	Park	**13_03b_**	-0.958579882	**-17.2662722**	16
**2003**	Park	**13_03b_**	-0.958579882	**-10.2662722**	15
**2003**	Park	**13_03b_**	-0.958579882	**0.73372781**	16
**2003**	Park	**13_03b_**	-0.958579882	**5.73372781**	17
**2003**	Park	**17_03b_**	-3.958579882	**-1.26627219**	17
**2003**	Park	**17_03b_**	-3.958579882	**4.73372781**	16.5
**2003**	Park	**17_03b_**	-3.958579882	**8.73372781**	17.5
**2003**	Park	**17_03b_**	-3.958579882	**11.7337278**	18
**2003**	Park	**17_03b_**	-3.958579882	**23.7337278**	18
**2003**	Park	**25_03b_**	9.041420118	**-18.2662722**	14.5
**2003**	Park	**25_03b_**	9.041420118	**-15.2662722**	14
**2003**	Park	**25_03b_**	9.041420118	**-11.2662722**	14.5
**2003**	Park	**25_03b_**	9.041420118	**-9.26627219**	13.5
**2003**	Park	**25_03b_**	9.041420118	**-3.26627219**	14.5
**2003**	Park	**31_03b_**	7.041420118	**-2.26627219**	12
**2003**	Park	**31_03b_**	7.041420118	**3.73372781**	13.5
**2003**	Park	**31_03b_**	7.041420118	**5.73372781**	11
**2003**	Park	**31_03b_**	7.041420118	**11.7337278**	11.5
**2003**	Park	**31_03b_**	7.041420118	**14.7337278**	15
**2003**	Park	**46_03b_**	4.041420118	**-4.26627219**	17.5
**2003**	Park	**46_03b_**	4.041420118	**-3.26627219**	17
**2003**	Park	**46_03b_**	4.041420118	**-0.26627219**	18.5
**2003**	Park	**46_03b_**	4.041420118	**4.73372781**	16.5
**2003**	Park	**49_03b_**	2.041420118	**-3.26627219**	17.5
**2003**	Park	**49_03b_**	2.041420118	**7.73372781**	17.5
**2003**	Park	**49_03b_**	2.041420118	**11.7337278**	17
**2003**	Park	**49_03b_**	2.041420118	**14.7337278**	17.5
**2003**	Park	**49_03b_**	2.041420118	**18.7337278**	15.5
**2003**	Park	**57_03b_**	2.041420118	**-1.26627219**	17
**2003**	Park	**57_03b_**	2.041420118	**10.7337278**	17.5
**2003**	Park	**57_03b_**	2.041420118	**20.7337278**	19.5
**2003**	Park	**57_03b_**	2.041420118	**22.7337278**	16.5
**2003**	Park	**57_03b_**	2.041420118	**23.7337278**	18
**2003**	Park	**60_03b_**	5.041420118	**-33.2662722**	14.5
**2003**	Park	**60_03b_**	5.041420118	**-32.2662722**	15
**2003**	Park	**60_03b_**	5.041420118	**-31.2662722**	16
**2003**	Park	**60_03b_**	5.041420118	**-24.2662722**	15.5
**2003**	Park	**60_03b_**	5.041420118	**-15.2662722**	16
**2003**	Park	**62_03b_**	1.041420118	**-11.2662722**	16.5
**2003**	Park	**62_03b_**	1.041420118	**-6.26627219**	18.5
**2003**	Park	**62_03b_**	1.041420118	**-3.26627219**	17
**2003**	Park	**62_03b_**	1.041420118	**-1.26627219**	18
**2003**	Park	**62_03b_**	1.041420118	**16.7337278**	16.5
**2003**	Park	**63_03b_**	0.041420118	**13.7337278**	18
**2003**	Park	**63_03b_**	0.041420118	**13.7337278**	18.5
**2003**	Park	**63_03b_**	0.041420118	**17.7337278**	18
**2003**	Park	**63_03b_**	0.041420118	**22.7337278**	18.5
**2003**	Park	**63_03b_**	0.041420118	**31.7337278**	18
**2003**	Park	**67_03b_**	-0.958579882	**-3.26627219**	16
**2003**	Park	**67_03b_**	-0.958579882	**-1.26627219**	16
**2003**	Park	**67_03b_**	-0.958579882	**1.73372781**	17

**2003**	Park	**67_03b_**	-0.958579882	**6.73372781**	16.5
**2003**	Park	**67_03b_**	-0.958579882	**11.7337278**	17.5
**2003**	Park	**81_03b_**	1.041420118	**6.73372781**	17.5
**2003**	Park	**81_03b_**	1.041420118	**11.7337278**	17.5
**2003**	Park	**81_03b_**	1.041420118	**12.7337278**	18.5
**2003**	Park	**81_03b_**	1.041420118	**24.7337278**	17.5
**2003**	Park	**81_03b_**	1.041420118	**28.7337278**	18.5
**2003**	Park	**85_03b_**	-2.958579882	**-21.2662722**	17.5
**2003**	Park	**85_03b_**	-2.958579882	**2.73372781**	17
**2003**	Park	**85_03b_**	-2.958579882	**13.7337278**	18
**2003**	Park	**85_03b_**	-2.958579882	**24.7337278**	17.5
**2003**	Park	**85_03b_**	-2.958579882	**29.7337278**	17
**2003**	Park	**96_03b_**	3.041420118	**-21.2662722**	16
**2003**	Park	**96_03b_**	3.041420118	**-16.2662722**	17.5
**2003**	Park	**96_03b_**	3.041420118	**-7.26627219**	17.5
**2003**	Park	**96_03b_**	3.041420118	**-5.26627219**	15.5
**2003**	Park	**96_03b_**	3.041420118	**-1.26627219**	17
**2003**	Park	**102_03b_**	-0.958579882	**4.73372781**	17
**2003**	Park	**102_03b_**	-0.958579882	**14.7337278**	16
**2003**	Park	**102_03b_**	-0.958579882	**20.7337278**	19
**2003**	Park	**102_03b_**	-0.958579882	**20.7337278**	18.5
**2003**	Park	**110_03b_**	-2.958579882	**-11.2662722**	10
**2003**	Park	**110_03b_**	-2.958579882	**-9.26627219**	14.5
**2003**	Park	**110_03b_**	-2.958579882	**-3.26627219**	13.5
**2003**	Park	**110_03b_**	-2.958579882	**0.73372781**	13.5
**2003**	Park	**110_03b_**	-2.958579882	**8.73372781**	10
**2003**	Park	**110_03b_**	-2.958579882	**8.73372781**	14.5
**2003**	Park	**110_03b_**	-2.958579882	**9.73372781**	15.5
**2003**	Park	**110_03b_**	-2.958579882	**11.7337278**	12
**2003**	Park	**111_03b_**	-0.958579882	**-6.26627219**	17.5
**2003**	Park	**111_03b_**	-0.958579882	**-0.26627219**	18.5
**2003**	Park	**111_03b_**	-0.958579882	**-0.26627219**	18
**2003**	Park	**111_03b_**	-0.958579882	**4.73372781**	19
**2003**	Park	**111_03b_**	-0.958579882	**9.73372781**	17.5
**2003**	Park	**116_03b_**	0.041420118	**11.7337278**	16
**2003**	Park	**116_03b_**	0.041420118	**13.7337278**	16
**2003**	Park	**116_03b_**	0.041420118	**15.7337278**	15.5
**2003**	Park	**116_03b_**	0.041420118	**15.7337278**	17
**2003**	Park	**116_03b_**	0.041420118	**25.7337278**	16
**2003**	Park	**125_03b_**	-2.958579882	**5.73372781**	14.5
**2003**	Park	**125_03b_**	-2.958579882	**7.73372781**	16.5
**2003**	Park	**125_03b_**	-2.958579882	**9.73372781**	16.5
**2003**	Park	**125_03b_**	-2.958579882	**14.7337278**	16
**2003**	Park	**125_03b_**	-2.958579882	**16.7337278**	17
**2003**	Park	**128_03b_**	-0.958579882	**1.73372781**	16.5
**2003**	Park	**128_03b_**	-0.958579882	**6.73372781**	16.5
**2003**	Park	**128_03b_**	-0.958579882	**6.73372781**	16.5
**2003**	Park	**128_03b_**	-0.958579882	**7.73372781**	16.5
**2003**	Park	**128_03b_**	-0.958579882	**8.73372781**	17
**2003**	Park	**9_03z_**	-0.958579882	**-25.2662722**	16
**2003**	Park	**9_03z_**	-0.958579882	**-18.2662722**	15.5
**2003**	Park	**9_03z_**	-0.958579882	**-14.2662722**	17
**2003**	Park	**13_03z_**	2.041420118	**-87.2662722**	9.5
**2003**	Park	**13_03z_**	2.041420118	**-73.2662722**	9.5

**2003**	Park	**13_03z_**	2.041420118	**-68.2662722**	10.5
**2003**	Park	**13_03z_**	2.041420118	**-59.2662722**	10
**2003**	Park	**13_03z_**	2.041420118	**-47.2662722**	12.5
**2003**	Park	**16_03z_**	1.041420118	**-7.26627219**	16.5
**2003**	Park	**16_03z_**	1.041420118	**-7.26627219**	15.5
**2003**	Park	**16_03z_**	1.041420118	**-1.26627219**	15.5
**2003**	Park	**16_03z_**	1.041420118	**4.73372781**	14.5
**2003**	Park	**16_03z_**	1.041420118	**11.7337278**	17
**2003**	Park	**23_03z_**	3.041420118	**17.7337278**	18
**2003**	Park	**23_03z_**	3.041420118	**18.7337278**	17
**2003**	Park	**23_03z_**	3.041420118	**20.7337278**	18
**2003**	Park	**23_03z_**	3.041420118	**24.7337278**	17
**2003**	Park	**23_03z_**	3.041420118	**32.7337278**	17
**2003**	Park	**27_03z_**	0.041420118	**-26.2662722**	16.5
**2003**	Park	**27_03z_**	0.041420118	**-19.2662722**	17
**2003**	Park	**27_03z_**	0.041420118	**-18.2662722**	18
**2003**	Park	**27_03z_**	0.041420118	**-15.2662722**	18.5
**2003**	Park	**27_03z_**	0.041420118	**-10.2662722**	17
**2003**	Park	**41_03z_**	-0.958579882	**-33.2662722**	16
**2003**	Park	**41_03z_**	-0.958579882	**-32.2662722**	16
**2003**	Park	**41_03z_**	-0.958579882	**-29.2662722**	14
**2003**	Park	**41_03z_**	-0.958579882	**-18.2662722**	16
**2003**	Park	**41_03z_**	-0.958579882	**-0.26627219**	15.5
**2003**	Park	**42_03z_**	2.041420118	**-19.2662722**	8
**2003**	Park	**42_03z_**	2.041420118	**-9.26627219**	15
**2003**	Park	**42_03z_**	2.041420118	**-9.26627219**	14
**2003**	Park	**42_03z_**	2.041420118	**2.73372781**	17
**2003**	Park	**42_03z_**	2.041420118	**10.7337278**	14
**2003**	Park	**45_03z_**	6.041420118	**-22.2662722**	16
**2003**	Park	**45_03z_**	6.041420118	**-12.2662722**	16.5
**2003**	Park	**45_03z_**	6.041420118	**-7.26627219**	16.5
**2003**	Park	**45_03z_**	6.041420118	**-6.26627219**	16
**2003**	Park	**45_03z_**	6.041420118	**-1.26627219**	14
**2003**	Park	**47_03z_**	-8.958579882	**-11.2662722**	12
**2003**	Park	**47_03z_**	-8.958579882	**-2.26627219**	15
**2003**	Park	**47_03z_**	-8.958579882	**0.73372781**	16
**2003**	Park	**47_03z_**	-8.958579882	**9.73372781**	16
**2003**	Park	**47_03z_**	-8.958579882	**11.7337278**	17
**2003**	Park	**47_03z_**	-8.958579882	**11.7337278**	16
**2003**	Park	**47_03z_**	-8.958579882	**15.7337278**	16
**2003**	Park	**47_03z_**	-8.958579882	**15.7337278**	15.5
**2003**	Park	**47_03z_**	-8.958579882	**18.7337278**	14.5
**2003**	Park	**47_03z_**	-8.958579882	**20.7337278**	15.5
**2003**	Park	**47_03z_**	-8.958579882	**26.7337278**	16.5
**2003**	Park	**49_03z_**	-0.958579882	**-7.26627219**	17
**2003**	Park	**49_03z_**	-0.958579882	**-1.26627219**	17.5
**2003**	Park	**49_03z_**	-0.958579882	**0.73372781**	17.5
**2003**	Park	**49_03z_**	-0.958579882	**9.73372781**	18.5
**2003**	Park	**49_03z_**	-0.958579882	**10.7337278**	16.5
**2004**	Forest	**41_04L1_**	-3.222222222	**-11.7333333**	17.5
**2004**	Forest	**41_04L1_**	-3.222222222	**-9.73333333**	17.5
**2004**	Forest	**41_04L1_**	-3.222222222	**-4.73333333**	16.5
**2004**	Forest	**41_04L1_**	-3.222222222	**-1.73333333**	18
**2004**	Forest	**41_04L1_**	-3.222222222	**4.26666667**	17.5

**2004**	Forest	**54_04L1_**	-0.222222222	**-24.7333333**	16.5
**2004**	Forest	**54_04L1_**	-0.222222222	**-23.7333333**	15.5
**2004**	Forest	**54_04L1_**	-0.222222222	**-19.7333333**	17
**2004**	Forest	**54_04L1_**	-0.222222222	**-10.7333333**	17.5
**2004**	Forest	**54_04L1_**	-0.222222222	**-1.73333333**	18
**2004**	Forest	**56_04L1_**	-3.222222222	**1.26666667**	18
**2004**	Forest	**56_04L1_**	-3.222222222	**5.26666667**	17
**2004**	Forest	**56_04L1_**	-3.222222222	**17.2666667**	18
**2004**	Forest	**56_04L1_**	-3.222222222	**18.2666667**	18
**2004**	Forest	**56_04L1_**	-3.222222222	**35.2666667**	17.5
**2004**	Forest	**80_04L1_**	5.777777778	**-7.73333333**	16
**2004**	Forest	**80_04L1_**	5.777777778	**-7.73333333**	15.5
**2004**	Forest	**80_04L1_**	5.777777778	**-4.73333333**	17.5
**2004**	Forest	**80_04L1_**	5.777777778	**4.26666667**	16
**2004**	Forest	**80_04L1_**	5.777777778	**10.2666667**	15.5
**2004**	Forest	**86_04L1_**	-3.222222222	**-4.73333333**	19.5
**2004**	Forest	**86_04L1_**	-3.222222222	**0.26666667**	18.5
**2004**	Forest	**86_04L1_**	-3.222222222	**0.26666667**	18
**2004**	Forest	**86_04L1_**	-3.222222222	**5.26666667**	17.5
**2004**	Forest	**86_04L1_**	-3.222222222	**20.2666667**	17.5
**2004**	Forest	**145_04L1_**	-0.222222222	**-7.73333333**	18
**2004**	Forest	**145_04L1_**	-0.222222222	**7.26666667**	17.5
**2004**	Forest	**145_04L1_**	-0.222222222	**8.26666667**	17.5
**2004**	Forest	**145_04L1_**	-0.222222222	**11.2666667**	19
**2004**	Forest	**145_04L1_**	-0.222222222	**14.2666667**	17.5
**2004**	Forest	**152_04L1_**	5.777777778	**-16.7333333**	15.5
**2004**	Forest	**152_04L1_**	5.777777778	**-4.73333333**	14.5
**2004**	Forest	**152_04L1_**	5.777777778	**-3.73333333**	16
**2004**	Forest	**152_04L1_**	5.777777778	**6.26666667**	16.5
**2004**	Forest	**152_04L1_**	5.777777778	**8.26666667**	16.5
**2004**	Forest	**174_04L1_**	1.777777778	**-12.7333333**	17.5
**2004**	Forest	**174_04L1_**	1.777777778	**-1.73333333**	17.5
**2004**	Forest	**174_04L1_**	1.777777778	**-0.73333333**	18.5
**2004**	Forest	**174_04L1_**	1.777777778	**2.26666667**	20
**2004**	Forest	**174_04L1_**	1.777777778	**2.26666667**	19
**2004**	Forest	**179_04L1_**	-3.222222222	**-5.73333333**	18.5
**2004**	Forest	**179_04L1_**	-3.222222222	**-0.73333333**	18
**2004**	Forest	**179_04L1_**	-3.222222222	**-0.73333333**	18.5
**2004**	Forest	**179_04L1_**	-3.222222222	**2.26666667**	18.5
**2004**	Forest	**179_04L1_**	-3.222222222	**4.26666667**	17.5
**2004**	Park	**2_04b_**	-4.559322034	**-8.86440678**	14.9
**2004**	Park	**2_04b_**	-4.559322034	**2.13559322**	14.6
**2004**	Park	**2_04b_**	-4.559322034	**4.13559322**	15.1
**2004**	Park	**2_04b_**	-4.559322034	**5.13559322**	15.6
**2004**	Park	**2_04b_**	-4.559322034	**10.1355932**	15.7
**2004**	Park	**9_04b_**	3.440677966	**-15.8644068**	14.5
**2004**	Park	**9_04b_**	3.440677966	**-9.86440678**	14
**2004**	Park	**9_04b_**	3.440677966	**-4.86440678**	13.5
**2004**	Park	**9_04b_**	3.440677966	**1.13559322**	15.5
**2004**	Park	**9_04b_**	3.440677966	**7.13559322**	14.5
**2004**	Park	**20_04b_**	-5.559322034	**-8.86440678**	16.5
**2004**	Park	**20_04b_**	-5.559322034	**-2.86440678**	16
**2004**	Park	**20_04b_**	-5.559322034	**0.13559322**	15.5
**2004**	Park	**20_04b_**	-5.559322034	**7.13559322**	15.5

**2004**	Park	**20_04b_**	-5.559322034	**14.1355932**		17
**2004**	Park	**53_04b_**	5.440677966	**-3.86440678**		16
**2004**	Park	**53_04b_**	5.440677966	**-2.86440678**		16
**2004**	Park	**53_04b_**	5.440677966	**4.13559322**		17
**2004**	Park	**53_04b_**	5.440677966	**5.13559322**		16
**2004**	Park	**53_04b_**	5.440677966	**22.1355932**		15.5
**2004**	Park	**60_04b_**	4.440677966	**-23.8644068**		16
**2004**	Park	**60_04b_**	4.440677966	**-5.86440678**		16
**2004**	Park	**60_04b_**	4.440677966	**-5.86440678**		15
**2004**	Park	**60_04b_**	4.440677966	**-1.86440678**		15.5
**2004**	Park	**60_04b_**	4.440677966	**12.1355932**		15
**2004**	Park	**61_04b_**	1.440677966	**9.13559322**		14
**2004**	Park	**61_04b_**	1.440677966	**10.1355932**		13.5
**2004**	Park	**61_04b_**	1.440677966	**11.1355932**		15
**2004**	Park	**61_04b_**	1.440677966	**13.1355932**		13
**2004**	Park	**61_04b_**	1.440677966	**23.1355932**		15
**2004**	Park	**64_04b_**	0.440677966	**5.13559322**		15.5
**2004**	Park	**64_04b_**	0.440677966	**6.13559322**		15
**2004**	Park	**64_04b_**	0.440677966	**11.1355932**		15.5
**2004**	Park	**64_04b_**	0.440677966	**19.1355932**		15
**2004**	Park	**64_04b_**	0.440677966	**28.1355932**		14.5
**2004**	Park	**65_04b_**	1.440677966	**-11.8644068**		17
**2004**	Park	**65_04b_**	1.440677966	**-9.86440678**		15.5
**2004**	Park	**65_04b_**	1.440677966	**-4.86440678**		16
**2004**	Park	**65_04b_**	1.440677966	**1.13559322**		16.5
**2004**	Park	**67_04b_**	0.440677966	**-28.8644068**		13.5
**2004**	Park	**67_04b_**	0.440677966	**-15.8644068**		15.5
**2004**	Park	**67_04b_**	0.440677966	**-13.8644068**		14.5
**2004**	Park	**67_04b_**	0.440677966	**-10.8644068**		15.5
**2004**	Park	**67_04b_**	0.440677966	**-9.86440678**		14
**2004**	Park	**77_04b_**	1.440677966	**-11.8644068**		12
**2004**	Park	**77_04b_**	1.440677966	**0.13559322**		14.5
**2004**	Park	**77_04b_**	1.440677966	**5.13559322**		13
**2004**	Park	**77_04b_**	1.440677966	**8.13559322**		14.5
**2004**	Park	**77_04b_**	1.440677966	**12.1355932**		14
**2004**	Park	**92_04b_**	-2.559322034	**1.13559322**		17.5
**2004**	Park	**92_04b_**	-2.559322034	**2.13559322**		18.5
**2004**	Park	**92_04b_**	-2.559322034	**5.13559322**		16
**2004**	Park	**92_04b_**	-2.559322034	**9.13559322**		16.5
**2004**	Park	**92_04b_**	-2.559322034	**10.1355932**		15.5
**2004**	Park	**105_04b_**	-5.559322034	**-23.8644068**		13.7
**2004**	Park	**105_04b_**	-5.559322034	**-17.8644068**		15
**2004**	Park	**105_04b_**	-5.559322034	**-14.8644068**		15
**2004**	Park	**105_04b_**	-5.559322034	**-14.8644068**		14.4
**2004**	Park	**105_04b_**	-5.559322034	**-0.86440678**		16
**2005**	Forest	**35_05L1_**	-0.076923077	**9.06153846**	-6.0925926	17.9
**2005**	Forest	**35_05L1_**	-0.076923077	**23.0615385**	17.907407	18.4
**2005**	Forest	**35_05L1_**	-0.076923077	**26.0615385**	17.907407	17.5
**2005**	Forest	**35_05L1_**	-0.076923077	**-5.93846154**	38.907407	13.5
**2005**	Forest	**35_05L1_**	-0.076923077	**16.0615385**	63.907407	17.1
**2005**	Forest	**46_05L1_**	-4.076923077	**7.06153846**	63.907407	17.2
**2005**	Forest	**46_05L1_**	-4.076923077	**-7.93846154**	107.90741	16.8
**2005**	Forest	**46_05L1_**	-4.076923077	**-5.93846154**	107.90741	16.9
**2005**	Forest	**46_05L1_**	-4.076923077	**7.06153846**	107.90741	17.9

**2005**	Forest	**46_05L1_**	-4.076923077	**14.0615385**	107.90741	18.2
**2005**	Forest	**57_05L1_**	-2.076923077	**12.0615385**	0.9074074	17.3
**2005**	Forest	**57_05L1_**	-2.076923077	**21.0615385**	25.907407	16.9
**2005**	Forest	**57_05L1_**	-2.076923077	**7.06153846**	44.907407	15.9
**2005**	Forest	**57_05L1_**	-2.076923077	**10.0615385**	68.907407	16.7
**2005**	Forest	**57_05L1_**	-2.076923077	**19.0615385**	107.90741	16.7
**2005**	Forest	**62_05L1_**	-5.076923077	**4.06153846**	-78.092593	15.9
**2005**	Forest	**62_05L1_**	-5.076923077	**23.0615385**	-48.092593	16.5
**2005**	Forest	**62_05L1_**	-5.076923077	**22.0615385**	-23.092593	14.5
**2005**	Forest	**62_05L1_**	-5.076923077	**2.06153846**	-22.092593	16.8
**2005**	Forest	**62_05L1_**	-5.076923077	**10.0615385**		15.1
**2005**	Forest	**73_05L1_**	8.923076923	**-50.9384615**	-118.09259	14.2
**2005**	Forest	**73_05L1_**	8.923076923	**-21.9384615**	-78.092593	14.5
**2005**	Forest	**73_05L1_**	8.923076923	**-17.9384615**	-75.092593	15.1
**2005**	Forest	**73_05L1_**	8.923076923	**-40.9384615**	-55.092593	14.1
**2005**	Forest	**73_05L1_**	8.923076923	**-24.9384615**	-38.092593	15.5
**2005**	Forest	**78_05L1_**	-5.076923077	**-12.9384615**		14.8
**2005**	Forest	**78_05L1_**	-5.076923077	**12.0615385**		17.6
**2005**	Forest	**78_05L1_**	-5.076923077	**14.0615385**		18.5
**2005**	Forest	**78_05L1_**	-5.076923077	**17.0615385**		17
**2005**	Forest	**78_05L1_**	-5.076923077	**34.0615385**		18
**2005**	Forest	**83_05L1_**	-4.076923077	**-19.9384615**		15.5
**2005**	Forest	**83_05L1_**	-4.076923077	**-5.93846154**		16.6
**2005**	Forest	**83_05L1_**	-4.076923077	**5.06153846**		16.4
**2005**	Forest	**83_05L1_**	-4.076923077	**7.06153846**		12.9
**2005**	Forest	**83_05L1_**	-4.076923077	**9.06153846**		18.3
**2005**	Forest	**89_05L1_**	-6.076923077	**-5.93846154**	-44.092593	10.6
**2005**	Forest	**92_05L1_**	13.92307692	**-1.93846154**	-82.092593	15.2
**2005**	Forest	**92_05L1_**	13.92307692	**-21.9384615**	-57.092593	15.4
**2005**	Forest	**92_05L1_**	13.92307692	**-2.93846154**	-57.092593	15.8
**2005**	Forest	**92_05L1_**	13.92307692	**-6.93846154**	-44.092593	15.7
**2005**	Forest	**92_05L1_**	13.92307692	**-11.9384615**	45.907407	14.8
**2005**	Forest	**93_05L1_**	-1.076923077	**5.06153846**	31.907407	16
**2005**	Forest	**93_05L1_**	-1.076923077	**-2.93846154**	41.907407	15.5
**2005**	Forest	**93_05L1_**	-1.076923077	**3.06153846**	46.907407	18.3
**2005**	Forest	**93_05L1_**	-1.076923077	**14.0615385**	51.907407	17.2
**2005**	Forest	**93_05L1_**	-1.076923077	**12.0615385**	57.907407	17
**2005**	Forest	**105_05L1_**	8.923076923	**2.06153846**	-99.092593	18.1
**2005**	Forest	**105_05L1_**	8.923076923	**11.0615385**	-28.092593	18
**2005**	Forest	**105_05L1_**	8.923076923	**-21.9384615**	24.907407	17.7
**2005**	Forest	**105_05L1_**	8.923076923	**-9.93846154**	47.907407	17.7
**2005**	Forest	**105_05L1_**	8.923076923	**2.06153846**	72.907407	17.1
**2005**	Forest	**107_05L1_**	-1.076923077	**7.06153846**	-12.092593	18
**2005**	Forest	**107_05L1_**	-1.076923077	**20.0615385**	-0.0925926	16.9
**2005**	Forest	**107_05L1_**	-1.076923077	**20.0615385**	20.907407	17
**2005**	Forest	**107_05L1_**	-1.076923077	**10.0615385**	33.907407	18.9
**2005**	Forest	**107_05L1_**	-1.076923077	**13.0615385**	43.907407	17.8
**2005**	Forest	**125_05L1_**	-3.076923077	**-24.9384615**	-111.09259	11.3
**2005**	Forest	**125_05L1_**	-3.076923077	**-19.9384615**	-95.092593	13.4
**2005**	Forest	**125_05L1_**	-3.076923077	**-26.9384615**	-92.092593	12.9
**2005**	Forest	**125_05L1_**	-3.076923077	**-22.9384615**	-88.092593	12
**2005**	Forest	**125_05L1_**	-3.076923077	**-44.9384615**	-74.092593	12
**2005**	Park	**14_05b_**	-3	**-27.5333333**		12.1
**2005**	Park	**14_05b_**	-3	**-19.5333333**		12.8

**2005**	Park	**14_05b_**	-3	**-1.53333333**		14.5
**2005**	Park	**14_05b_**	-3	**1.46666667**		12.4
**2005**	Park	**14_05b_**	-3	**8.46666667**		14.5
**2005**	Park	**26_05b_**	-5	**-9.53333333**		16.3
**2005**	Park	**26_05b_**	-5	**2.46666667**		16.7
**2005**	Park	**26_05b_**	-5	**6.46666667**		17.1
**2005**	Park	**26_05b_**	-5	**9.46666667**		17
**2005**	Park	**26_05b_**	-5	**13.4666667**		17.9
**2005**	Park	**35_05b_**	-3	**-4.53333333**		15.1
**2005**	Park	**35_05b_**	-3	**-2.53333333**		15.5
**2005**	Park	**35_05b_**	-3	**-0.53333333**		15.6
**2005**	Park	**35_05b_**	-3	**2.46666667**		13.3
**2005**	Park	**35_05b_**	-3	**3.46666667**		12.1
**2005**	Park	**39_05b_**	14	**-15.5333333**	-79.6	15.7
**2005**	Park	**39_05b_**	14	**22.4666667**	-63.6	17.6
**2005**	Park	**39_05b_**	14	**1.46666667**	-44.6	17.7
**2005**	Park	**39_05b_**	14	**6.46666667**	-8.6	17.1
**2005**	Park	**39_05b_**	14	**21.4666667**	37.4	18
**2005**	Park	**44_05b_**	5	**-20.5333333**		17.6
**2005**	Park	**44_05b_**	5	**-16.5333333**		15.9
**2005**	Park	**44_05b_**	5	**-15.5333333**		16
**2005**	Park	**44_05b_**	5	**-14.5333333**		16.7
**2005**	Park	**44_05b_**	5	**-5.53333333**		15.7
**2005**	Park	**46_05b_**	-4	**-0.53333333**		16.3
**2005**	Park	**46_05b_**	-4	**14.4666667**		18.6
**2005**	Park	**46_05b_**	-4	**15.4666667**		14.3
**2005**	Park	**46_05b_**	-4	**20.4666667**		14.7
**2005**	Park	**46_05b_**	-4	**24.4666667**		18.7
**2005**	Park	**48_05b_**	-6	**10.4666667**		16.1
**2005**	Park	**48_05b_**	-6	**11.4666667**		14.9
**2005**	Park	**48_05b_**	-6	**15.4666667**		16
**2005**	Park	**48_05b_**	-6	**24.4666667**		16
**2005**	Park	**48_05b_**	-6	**25.4666667**		15.6
**2005**	Park	**55_05b_**	-2	**6.46666667**	-35.6	14.3
**2005**	Park	**55_05b_**	-2	**11.4666667**	25.4	12.9
**2005**	Park	**55_05b_**	-2	**-10.5333333**	28.4	11.5
**2005**	Park	**55_05b_**	-2	**3.46666667**	59.4	13.7
**2005**	Park	**55_05b_**	-2	**24.4666667**	78.4	12.7
**2005**	Park	**76_05b_**	-2	**2.46666667**	-33.6	15.1
**2005**	Park	**76_05b_**	-2	**23.4666667**	41.4	15.9
**2005**	Park	**76_05b_**	-2	**19.4666667**	84.4	
**2005**	Park	**76_05b_**	-2	**11.4666667**	95.4	15.9
**2005**	Park	**76_05b_**	-2	**7.46666667**	106.4	15.5
**2005**	Park	**110_05b_**	9	**2.46666667**	-43.6	18.2
**2005**	Park	**110_05b_**	9	**-37.5333333**	-9.6	17.4
**2005**	Park	**110_05b_**	9	**7.46666667**	4.4	17.6
**2005**	Park	**110_05b_**	9	**-8.53333333**	25.4	18.5
**2005**	Park	**110_05b_**	9	**-5.53333333**	53.4	17.8
**2005**	Park	**117_05b_**	-7	**18.4666667**	-84.6	16.9
**2005**	Park	**117_05b_**	-7	**9.46666667**	-82.6	15.1
**2005**	Park	**117_05b_**	-7	**16.4666667**	-55.6	15.9
**2005**	Park	**117_05b_**	-7	**5.46666667**	-48.6	15
**2005**	Park	**117_05b_**	-7	**11.4666667**	-42.6	13.6
**2005**	Park	**122_05b_**	4	**-39.5333333**	-71.6	12

**2005**	Park	**122_05b_**	4	**-35.5333333**	-56.6	12.7
**2005**	Park	**122_05b_**	4	**-33.5333333**	-1.6	12.7
**2005**	Park	**122_05b_**	4	**-61.5333333**	52.4	10.3
**2005**	Park	**122_05b_**	4	**-56.5333333**	70.4	10.1
**2006**	Forest	**13_06L1_**	-0.534883721	**-7.6627907**		18
**2006**	Forest	**13_06L1_**	-0.534883721	**-2.6627907**		16.4
**2006**	Forest	**13_06L1_**	-0.534883721	**-2.6627907**		17
**2006**	Forest	**13_06L1_**	-0.534883721	**-0.6627907**		15.8
**2006**	Forest	**13_06L1_**	-0.534883721	**1.3372093**		18.1
**2006**	Forest	**21_06L1_**	-0.534883721	**-8.6627907**	-70.487805	17.5
**2006**	Forest	**21_06L1_**	-0.534883721	**-8.6627907**	-30.487805	16.3
**2006**	Forest	**21_06L1_**	-0.534883721	**-15.6627907**	16.512195	16.6
**2006**	Forest	**21_06L1_**	-0.534883721	**-12.6627907**	48.512195	17.1
**2006**	Forest	**21_06L1_**	-0.534883721	**-11.6627907**	115.5122	16.5
**2006**	Forest	**28_06L1_**	-0.534883721	**-4.6627907**	-71.487805	16.5
**2006**	Forest	**28_06L1_**	-0.534883721	**25.3372093**	-63.487805	16
**2006**	Forest	**28_06L1_**	-0.534883721	**15.3372093**	-53.487805	14.4
**2006**	Forest	**28_06L1_**	-0.534883721	**38.3372093**	-24.487805	15.2
**2006**	Forest	**36_06L1_**	0.465116279	**9.3372093**	-80.487805	17.9
**2006**	Forest	**36_06L1_**	0.465116279	**13.3372093**	-70.487805	18.7
**2006**	Forest	**36_06L1_**	0.465116279	**4.3372093**	-49.487805	18
**2006**	Forest	**36_06L1_**	0.465116279	**3.3372093**	-25.487805	17.5
**2006**	Forest	**36_06L1_**	0.465116279	**8.3372093**	0.5121951	17.5
**2006**	Forest	**38_06L1_**	3.465116279	**-17.6627907**	-83.487805	16.7
**2006**	Forest	**38_06L1_**	3.465116279	**-16.6627907**	-61.487805	15.7
**2006**	Forest	**38_06L1_**	3.465116279	**-5.6627907**	-37.487805	16.5
**2006**	Forest	**38_06L1_**	3.465116279	**-1.6627907**	-6.4878049	16.4
**2006**	Forest	**38_06L1_**	3.465116279	**-6.6627907**	8.5121951	16.6
**2006**	Forest	**39_06L1_**	-0.534883721	**-16.6627907**	-44.487805	14.7
**2006**	Forest	**39_06L1_**	-0.534883721	**-7.6627907**	-28.487805	17.2
**2006**	Forest	**39_06L1_**	-0.534883721	**-5.6627907**	-22.487805	16.2
**2006**	Forest	**39_06L1_**	-0.534883721	**-8.6627907**	-1.4878049	16
**2006**	Forest	**39_06L1_**	-0.534883721	**-7.6627907**	0.5121951	14.6
**2006**	Forest	**58_06L1_**	0.465116279	**6.3372093**		17
**2006**	Forest	**58_06L1_**	0.465116279	**6.3372093**		16.7
**2006**	Forest	**58_06L1_**	0.465116279	**8.3372093**		16.9
**2006**	Forest	**58_06L1_**	0.465116279	**10.3372093**		16.6
**2006**	Forest	**58_06L1_**	0.465116279	**19.3372093**		17
**2006**	Forest	**70_06L1_**	1.465116279	**2.3372093**		18
**2006**	Forest	**70_06L1_**	1.465116279	**8.3372093**		17.6
**2006**	Forest	**70_06L1_**	1.465116279	**13.3372093**		16.8
**2006**	Forest	**70_06L1_**	1.465116279	**15.3372093**		17.4
**2006**	Forest	**70_06L1_**	1.465116279	**16.3372093**		18.4
**2006**	Forest	**83_06L1_**	1.465116279	**11.3372093**		16.1
**2006**	Forest	**83_06L1_**	1.465116279	**13.3372093**		17.2
**2006**	Forest	**83_06L1_**	1.465116279	**13.3372093**		17.6
**2006**	Forest	**83_06L1_**	1.465116279	**16.3372093**		16.5
**2006**	Forest	**83_06L1_**	1.465116279	**19.3372093**		16.1
**2006**	Forest	**96_06L1_**	-1.534883721	**-15.6627907**		17.8
**2006**	Forest	**96_06L1_**	-1.534883721	**-8.6627907**		17.4
**2006**	Forest	**96_06L1_**	-1.534883721	**-4.6627907**		16.3
**2006**	Forest	**96_06L1_**	-1.534883721	**-3.6627907**		16.5
**2006**	Forest	**96_06L1_**	-1.534883721	**1.3372093**		16.8
**2006**	Forest	**102_06L1_**	-4.534883721	**5.3372093**		16.9

**2006**	Forest	**102_06L1_**	-4.534883721	**11.3372093**		16.2
**2006**	Forest	**102_06L1_**	-4.534883721	**20.3372093**		18.2
**2006**	Forest	**102_06L1_**	-4.534883721	**26.3372093**		16.5
**2006**	Forest	**102_06L1_**	-4.534883721	**27.3372093**		17.9
**2006**	Forest	**110_06L1_**	-2.534883721	**-13.6627907**	23.512195	18.3
**2006**	Forest	**110_06L1_**	-2.534883721	**-9.6627907**	36.512195	17
**2006**	Forest	**110_06L1_**	-2.534883721	**-6.6627907**	40.512195	18.5
**2006**	Forest	**110_06L1_**	-2.534883721	**-9.6627907**	50.512195	17
**2006**	Forest	**110_06L1_**	-2.534883721	**-4.6627907**	111.5122	18.5
**2006**	Forest	**165_06L1_**	-0.534883721	**-7.6627907**		17.8
**2006**	Forest	**165_06L1_**	-0.534883721	**3.3372093**		18.3
**2006**	Forest	**165_06L1_**	-0.534883721	**4.3372093**		18
**2006**	Forest	**165_06L1_**	-0.534883721	**6.3372093**		17.4
**2006**	Forest	**165_06L1_**	-0.534883721	**11.3372093**		18.6
**2006**	Forest	**177_06L1_**	0.465116279	**11.3372093**		18.3
**2006**	Forest	**177_06L1_**	0.465116279	**13.3372093**		19.8
**2006**	Forest	**177_06L1_**	0.465116279	**17.3372093**		17.3
**2006**	Forest	**177_06L1_**	0.465116279	**21.3372093**		19
**2006**	Forest	**177_06L1_**	0.465116279	**24.3372093**		18.7
**2006**	Forest	**8_06L2_**	-2.534883721	**-1.6627907**	-79.487805	18.3
**2006**	Forest	**8_06L2_**	-2.534883721	**-14.6627907**	-60.487805	16.9
**2006**	Forest	**8_06L2_**	-2.534883721	**-12.6627907**	-51.487805	15.9
**2006**	Forest	**8_06L2_**	-2.534883721	**-12.6627907**	-33.487805	17.4
**2006**	Forest	**8_06L2_**	-2.534883721	**-4.6627907**	6.5121951	18.7
**2006**	Forest	**13_06L2_**	-3.534883721	**-23.6627907**	39.512195	17
**2006**	Forest	**13_06L2_**	-3.534883721	**-27.6627907**	68.512195	17.4
**2006**	Forest	**13_06L2_**	-3.534883721	**-40.6627907**	104.5122	16.8
**2006**	Forest	**13_06L2_**	-3.534883721	**-39.6627907**	113.5122	15.6
**2006**	Forest	**13_06L2_**	-3.534883721	**-32.6627907**	122.5122	16.3
**2006**	Forest	**15_06L2_**	-2.534883721	**-7.6627907**	61.512195	20.5
**2006**	Forest	**15_06L2_**	-2.534883721	**-2.6627907**	81.512195	19.1
**2006**	Forest	**33_06L2_**	10.46511628	**-12.6627907**		17.2
**2006**	Forest	**33_06L2_**	10.46511628	**-6.6627907**		14.7
**2006**	Forest	**33_06L2_**	10.46511628	**-4.6627907**		15.2
**2006**	Forest	**33_06L2_**	10.46511628	**-3.6627907**		15.5
**2006**	Forest	**33_06L2_**	10.46511628	**-0.6627907**		15.6
**2006**	Park	**2_06b_**	14.82417582	**0.89010989**		15.1
**2006**	Park	**2_06b_**	14.82417582	**3.89010989**		14.2
**2006**	Park	**2_06b_**	14.82417582	**3.89010989**		15.3
**2006**	Park	**2_06b_**	14.82417582	**16.8901099**		16.2
**2006**	Park	**2_06b_**	14.82417582	**25.8901099**		15.5
**2006**	Park	**13_06b_**	-3.175824176	**-1.10989011**	-51.804196	14.1
**2006**	Park	**13_06b_**	-3.175824176	**-7.10989011**	-41.804196	16
**2006**	Park	**13_06b_**	-3.175824176	**-6.10989011**	-36.804196	16.3
**2006**	Park	**13_06b_**	-3.175824176	**5.89010989**	-23.804196	14.7
**2006**	Park	**13_06b_**	-3.175824176	**-2.10989011**	40.195804	13.3
**2006**	Park	**17_06b_**	-3.175824176	**-14.1098901**	-49.804196	14.6
**2006**	Park	**17_06b_**	-3.175824176	**-8.10989011**	-17.804196	15.6
**2006**	Park	**17_06b_**	-3.175824176	**-18.1098901**	28.195804	13.5
**2006**	Park	**17_06b_**	-3.175824176	**-8.10989011**	44.195804	14.3
**2006**	Park	**17_06b_**	-3.175824176	**6.89010989**		13.7
**2006**	Park	**22_06b_**	3.824175824	**-7.10989011**	5.1958042	15.3
**2006**	Park	**22_06b_**	3.824175824	**-2.10989011**	12.195804	15.4
**2006**	Park	**22_06b_**	3.824175824	**-7.10989011**	23.195804	16.9

**2006**	Park	**22_06b_**	3.824175824	**-5.10989011**	29.195804	16.4
**2006**	Park	**22_06b_**	3.824175824	**-2.10989011**	35.195804	17.5
**2006**	Park	**23_06b_**	-0.175824176	**-1.10989011**	-54.804196	15.1
**2006**	Park	**23_06b_**	-0.175824176	**-17.1098901**	-51.804196	15.1
**2006**	Park	**23_06b_**	-0.175824176	**-5.10989011**	-42.804196	14.8
**2006**	Park	**23_06b_**	-0.175824176	**-10.1098901**	9.1958042	15.6
**2006**	Park	**23_06b_**	-0.175824176	**-10.1098901**	19.195804	16.1
**2006**	Park	**29_06b_**	0.824175824	**-27.1098901**	16.195804	10.4
**2006**	Park	**29_06b_**	0.824175824	**-10.1098901**	57.195804	12.1
**2006**	Park	**29_06b_**	0.824175824	**-4.10989011**	66.195804	12.6
**2006**	Park	**29_06b_**	0.824175824	**-21.1098901**	86.195804	11.6
**2006**	Park	**35_06b_**	-7.175824176	**-8.10989011**	-35.804196	15.6
**2006**	Park	**35_06b_**	-7.175824176	**-1.10989011**	25.195804	15.4
**2006**	Park	**35_06b_**	-7.175824176	**11.8901099**	57.195804	15.1
**2006**	Park	**35_06b_**	-7.175824176	**-7.10989011**	66.195804	16.1
**2006**	Park	**35_06b_**	-7.175824176	**-3.10989011**	69.195804	16.4
**2006**	Park	**43_06b_**	0.824175824	**-2.10989011**	-71.804196	13.4
**2006**	Park	**43_06b_**	0.824175824	**12.8901099**	-68.804196	13.1
**2006**	Park	**43_06b_**	0.824175824	**-2.10989011**	-44.804196	14.2
**2006**	Park	**43_06b_**	0.824175824	**10.8901099**	-17.804196	13.8
**2006**	Park	**43_06b_**	0.824175824	**-11.1098901**	8.1958042	13.4
**2006**	Park	**46_06b_**	0.824175824	**10.8901099**	-42.804196	16
**2006**	Park	**46_06b_**	0.824175824	**-13.1098901**	-39.804196	16.8
**2006**	Park	**46_06b_**	0.824175824	**-8.10989011**	-39.804196	16.3
**2006**	Park	**56_06b_**	2.824175824	**1.89010989**	5.1958042	17.1
**2006**	Park	**56_06b_**	2.824175824	**12.8901099**	6.1958042	17.2
**2006**	Park	**56_06b_**	2.824175824	**11.8901099**	7.1958042	17.7
**2006**	Park	**56_06b_**	2.824175824	**4.89010989**	13.195804	16.3
**2006**	Park	**56_06b_**	2.824175824	**9.89010989**	44.195804	16.7
**2006**	Park	**57_06b_**	-2.175824176	**9.89010989**	-33.804196	13.5
**2006**	Park	**57_06b_**	-2.175824176	**-5.10989011**	-21.804196	13.1
**2006**	Park	**57_06b_**	-2.175824176	**14.8901099**	-6.8041958	15.2
**2006**	Park	**57_06b_**	-2.175824176	**-0.10989011**	35.195804	14.4
**2006**	Park	**57_06b_**	-2.175824176	**-1.10989011**	53.195804	12.6
**2006**	Park	**60_06b_**	2.824175824	**33.8901099**	-32.804196	13.8
**2006**	Park	**60_06b_**	2.824175824	**25.8901099**	-7.8041958	15.8
**2006**	Park	**60_06b_**	2.824175824	**30.8901099**	3.1958042	15.1
**2006**	Park	**60_06b_**	2.824175824	**31.8901099**	7.1958042	16.3
**2006**	Park	**60_06b_**	2.824175824	**23.8901099**		16.9
**2006**	Park	**62_06b_**	0.824175824	**19.8901099**	-49.804196	17.4
**2006**	Park	**62_06b_**	0.824175824	**25.8901099**	-27.804196	17.9
**2006**	Park	**62_06b_**	0.824175824	**12.8901099**	-6.8041958	16.9
**2006**	Park	**62_06b_**	0.824175824	**11.8901099**	-1.8041958	16.6
**2006**	Park	**62_06b_**	0.824175824	**20.8901099**	62.195804	18.4
**2006**	Park	**63_06b_**	-2.175824176	**16.8901099**	-2.8041958	16.5
**2006**	Park	**63_06b_**	-2.175824176	**19.8901099**	-0.8041958	15.9
**2006**	Park	**63_06b_**	-2.175824176	**11.8901099**	3.1958042	15.7
**2006**	Park	**63_06b_**	-2.175824176	**19.8901099**	4.1958042	16.1
**2006**	Park	**63_06b_**	-2.175824176	**16.8901099**	30.195804	17.1
**2006**	Park	**64_06b_**	-1.175824176	**5.89010989**	-29.804196	15.6
**2006**	Park	**64_06b_**	-1.175824176	**1.89010989**	-24.804196	15
**2006**	Park	**64_06b_**	-1.175824176	**10.8901099**	-22.804196	15.9
**2006**	Park	**64_06b_**	-1.175824176	**7.89010989**	-16.804196	14
**2006**	Park	**64_06b_**	-1.175824176	**8.89010989**	-9.8041958	14.7

**2006**	Park	**69_06b_**	10.82417582	**-8.10989011**		13.4
**2006**	Park	**69_06b_**	10.82417582	**-7.10989011**		14.1
**2006**	Park	**69_06b_**	10.82417582	**-5.10989011**		12
**2006**	Park	**69_06b_**	10.82417582	**2.89010989**		13.8
**2006**	Park	**69_06b_**	10.82417582	**3.89010989**		13
**2006**	Park	**70_06b_**	0.824175824	**9.89010989**	-58.804196	16.8
**2006**	Park	**70_06b_**	0.824175824	**5.89010989**	-42.804196	17.1
**2006**	Park	**70_06b_**	0.824175824	**3.89010989**	-3.8041958	16.7
**2006**	Park	**70_06b_**	0.824175824	**-13.1098901**	11.195804	16.7
**2006**	Park	**70_06b_**	0.824175824	**-8.10989011**		17.2
**2006**	Park	**73_06b_**	1.824175824	**-3.10989011**	-5.8041958	16.9
**2006**	Park	**73_06b_**	1.824175824	**7.89010989**	8.1958042	18.6
**2006**	Park	**73_06b_**	1.824175824	**16.8901099**	10.195804	14.2
**2006**	Park	**73_06b_**	1.824175824	**12.8901099**	21.195804	19.7
**2006**	Park	**73_06b_**	1.824175824	**7.89010989**	44.195804	18.5
**2006**	Park	**75_06b_**	-1.175824176	**-8.10989011**	-33.804196	15.6
**2006**	Park	**75_06b_**	-1.175824176	**-4.10989011**	-33.804196	17.2
**2006**	Park	**75_06b_**	-1.175824176	**-5.10989011**	-29.804196	16.5
**2006**	Park	**75_06b_**	-1.175824176	**-15.1098901**	15.195804	16.3
**2006**	Park	**75_06b_**	-1.175824176	**-11.1098901**	34.195804	16.5
**2006**	Park	**81_06b_**	-2.175824176	**4.89010989**	-15.804196	16.2
**2006**	Park	**81_06b_**	-2.175824176	**6.89010989**	-11.804196	15.9
**2006**	Park	**81_06b_**	-2.175824176	**0.89010989**	-2.8041958	17.6
**2006**	Park	**81_06b_**	-2.175824176	**-5.10989011**	5.1958042	15.7
**2006**	Park	**81_06b_**	-2.175824176	**7.89010989**	30.195804	15.5
**2006**	Park	**90_06b_**	-1.175824176	**-22.1098901**	12.195804	16.4
**2006**	Park	**90_06b_**	-1.175824176	**-22.1098901**	27.195804	15
**2006**	Park	**90_06b_**	-1.175824176	**-25.1098901**	29.195804	14.9
**2006**	Park	**90_06b_**	-1.175824176	**-31.1098901**	47.195804	14.6
**2006**	Park	**90_06b_**	-1.175824176	**-24.1098901**	58.195804	15.3
**2006**	Park	**91_06b_**	-2.175824176	**-16.1098901**	-41.804196	13.3
**2006**	Park	**91_06b_**	-2.175824176	**-9.10989011**	-39.804196	12.3
**2006**	Park	**91_06b_**	-2.175824176	**-9.10989011**	25.195804	12.1
**2006**	Park	**91_06b_**	-2.175824176	**-18.1098901**	87.195804	13.5
**2006**	Park	**91_06b_**	-2.175824176	**-17.1098901**		13
**2006**	Park	**92_06b_**	-2.175824176	**9.89010989**		15
**2006**	Park	**92_06b_**	-2.175824176	**25.8901099**		16.8
**2006**	Park	**92_06b_**	-2.175824176	**27.8901099**		16.4
**2006**	Park	**92_06b_**	-2.175824176	**29.8901099**		16.2
**2006**	Park	**92_06b_**	-2.175824176	**30.8901099**		17.9
**2006**	Park	**93_06b_**	10.82417582	**-35.1098901**		12.3
**2006**	Park	**93_06b_**	10.82417582	**-31.1098901**		12.6
**2006**	Park	**93_06b_**	10.82417582	**-17.1098901**		14
**2006**	Park	**93_06b_**	10.82417582	**-14.1098901**		14.7
**2006**	Park	**93_06b_**	10.82417582	**-3.10989011**		14.2
**2006**	Park	**98_06b_**	-3.175824176	**-0.10989011**		16.7
**2006**	Park	**98_06b_**	-3.175824176	**0.89010989**		15.8
**2006**	Park	**98_06b_**	-3.175824176	**10.8901099**		15.6
**2006**	Park	**98_06b_**	-3.175824176	**11.8901099**		16.7
**2006**	Park	**98_06b_**	-3.175824176	**26.8901099**		15.3
**2006**	Park	**100_06b_**	-0.175824176	**-3.10989011**		15.7
**2006**	Park	**100_06b_**	-0.175824176	**0.89010989**		15
**2006**	Park	**100_06b_**	-0.175824176	**3.89010989**		15.3
**2006**	Park	**100_06b_**	-0.175824176	**8.89010989**		16.8

**2006**	Park	**100_06b_**	-0.175824176	**8.89010989**		16.3
**2006**	Park	**117_06b_**	-4.175824176	**14.8901099**	4.1958042	17.9
**2006**	Park	**117_06b_**	-4.175824176	**7.89010989**	20.195804	17.8
**2006**	Park	**117_06b_**	-4.175824176	**35.8901099**	23.195804	18.4
**2006**	Park	**117_06b_**	-4.175824176	**35.8901099**	64.195804	18.6
**2006**	Park	**117_06b_**	-4.175824176	**12.8901099**	109.1958	18.2
**2006**	Park	**125_06b_**	-4.175824176	**6.89010989**	-36.804196	17.3
**2006**	Park	**125_06b_**	-4.175824176	**0.89010989**	-6.8041958	17.3
**2006**	Park	**125_06b_**	-4.175824176	**5.89010989**	-5.8041958	16.7
**2006**	Park	**125_06b_**	-4.175824176	**-1.10989011**	-0.8041958	15.4
**2006**	Park	**125_06b_**	-4.175824176	**-3.10989011**	113.1958	15.5
**2006**	Park	**65a_06b_**	0.824175824	**15.8901099**	-37.804196	15.3
**2006**	Park	**65a_06b_**	0.824175824	**11.8901099**	-33.804196	14.8
**2006**	Park	**65a_06b_**	0.824175824	**10.8901099**	-31.804196	14.7
**2006**	Park	**65a_06b_**	0.824175824	**13.8901099**	-11.804196	15
**2006**	Park	**65a_06b_**	0.824175824	**5.89010989**	28.195804	16
**2006**	Park	**1_06z_**	-7.175824176	**-6.10989011**	26.195804	15.7
**2006**	Park	**1_06z_**	-7.175824176	**-16.1098901**	35.195804	16.7
**2006**	Park	**1_06z_**	-7.175824176	**-15.1098901**	45.195804	15.1
**2006**	Park	**1_06z_**	-7.175824176	**-4.10989011**	55.195804	13.6
**2006**	Park	**1_06z_**	-7.175824176	**-11.1098901**	155.1958	16.1
**2006**	Park	**2_06z_**	-3.175824176	**-31.1098901**	-36.804196	14
**2006**	Park	**2_06z_**	-3.175824176	**-69.1098901**	-10.804196	15.6
**2006**	Park	**2_06z_**	-3.175824176	**-31.1098901**	18.195804	16
**2006**	Park	**2_06z_**	-3.175824176	**-16.1098901**	50.195804	13.4
**2006**	Park	**2_06z_**	-3.175824176	**-42.1098901**	74.195804	17.3
**2006**	Park	**8_06z_**	-5.175824176	**-0.10989011**	-38.804196	15.8
**2006**	Park	**8_06z_**	-5.175824176	**-22.1098901**	-32.804196	16
**2006**	Park	**8_06z_**	-5.175824176	**-16.1098901**	0.1958042	14.7
**2006**	Park	**8_06z_**	-5.175824176	**-4.10989011**	3.1958042	18.4
**2006**	Park	**8_06z_**	-5.175824176	**-9.10989011**	33.195804	15.1
**2006**	Park	**14_06z_**	-0.175824176	**-18.1098901**	-65.804196	14.6
**2006**	Park	**14_06z_**	-0.175824176	**-5.10989011**	-62.804196	11.9
**2006**	Park	**14_06z_**	-0.175824176	**7.89010989**	-54.804196	13.6
**2006**	Park	**14_06z_**	-0.175824176	**-18.1098901**	-39.804196	13.5
**2006**	Park	**14_06z_**	-0.175824176	**-16.1098901**	7.1958042	14.2
**2006**	Park	**42_06z_**	-6.175824176	**-10.1098901**	-107.8042	17.1
**2006**	Park	**42_06z_**	-6.175824176	**-0.10989011**	-48.804196	17.3
**2006**	Park	**42_06z_**	-6.175824176	**5.89010989**	-11.804196	17.4
**2006**	Park	**42_06z_**	-6.175824176	**-8.10989011**	8.1958042	16.5
**2006**	Park	**42_06z_**	-6.175824176	**10.8901099**	12.195804	16.1
**2006**	Park	**43_06z_**	9.824175824	**-13.1098901**		13.9
**2006**	Park	**43_06z_**	9.824175824	**-0.10989011**		13
**2006**	Park	**43_06z_**	9.824175824	**9.89010989**		13.7
**2006**	Park	**43_06z_**	9.824175824	**12.8901099**		13.7
**2006**	Park	**43_06z_**	9.824175824	**16.8901099**		13
**2006**	Park	**45_06z_**	-0.175824176	**-0.10989011**	-84.804196	16.1
**2006**	Park	**45_06z_**	-0.175824176	**2.89010989**	-34.804196	16.6
**2006**	Park	**45_06z_**	-0.175824176	**21.8901099**	-33.804196	15.5
**2006**	Park	**45_06z_**	-0.175824176	**-4.10989011**	-6.8041958	16.6
**2006**	Park	**45_06z_**	-0.175824176	**7.89010989**	0.1958042	16.6
**2006**	Park	**51_06z_**	-0.175824176	**-15.1098901**	-90.804196	13.8
**2006**	Park	**51_06z_**	-0.175824176	**0.89010989**	-27.804196	15
**2006**	Park	**51_06z_**	-0.175824176	**-8.10989011**	-9.8041958	14.2

**2006**	Park	**51_06z_**	-0.175824176	**-19.1098901**	-8.8041958	14.4
**2006**	Park	**51_06z_**	-0.175824176	**-22.1098901**	-0.8041958	14.5
**2007**	Forest	**10_07L1_**	3.606741573	**-6.58426966**	-27.202247	16.3
**2007**	Forest	**10_07L1_**	3.606741573	**13.4157303**	16.797753	15.8
**2007**	Forest	**10_07L1_**	3.606741573	**-3.58426966**	48.797753	15.8
**2007**	Forest	**20_07L1_**	-1.393258427	**8.41573034**	-44.202247	15.1
**2007**	Forest	**20_07L1_**	-1.393258427	**-6.58426966**	-19.202247	14.8
**2007**	Forest	**20_07L1_**	-1.393258427	**2.41573034**	79.797753	15.3
**2007**	Forest	**31_07L1_**	-0.393258427	**5.41573034**	-39.202247	17.1
**2007**	Forest	**31_07L1_**	-0.393258427	**17.4157303**	-4.2022472	15.6
**2007**	Forest	**31_07L1_**	-0.393258427	**6.41573034**	16.797753	16.2
**2007**	Forest	**39_07L1_**	-3.393258427	**-0.58426966**	-32.202247	16.8
**2007**	Forest	**39_07L1_**	-3.393258427	**18.4157303**	-28.202247	17.3
**2007**	Forest	**39_07L1_**	-3.393258427	**12.4157303**	15.797753	17.4
**2007**	Forest	**40_07L1_**	-0.393258427	**-19.5842697**	-19.202247	15.7
**2007**	Forest	**40_07L1_**	-0.393258427	**-11.5842697**	-9.2022472	15.6
**2007**	Forest	**40_07L1_**	-0.393258427	**-10.5842697**	1.7977528	15.8
**2007**	Forest	**57_07L1_**	0.606741573	**-5.58426966**	-23.202247	14
**2007**	Forest	**57_07L1_**	0.606741573	**-27.5842697**	32.797753	13.5
**2007**	Forest	**57_07L1_**	0.606741573	**-8.58426966**	56.797753	14.7
**2007**	Forest	**58_07L1_**	2.606741573	**8.41573034**	-93.202247	13.1
**2007**	Forest	**58_07L1_**	2.606741573	**-0.58426966**	-17.202247	13.4
**2007**	Forest	**58_07L1_**	2.606741573	**-16.5842697**	-8.2022472	11.5
**2007**	Forest	**62_07L1_**	-0.393258427	**-9.58426966**	5.7977528	18.5
**2007**	Forest	**62_07L1_**	-0.393258427	**-12.5842697**	75.797753	17.4
**2007**	Forest	**62_07L1_**	-0.393258427	**9.41573034**	81.797753	16.9
**2007**	Forest	**64_07L1_**	1.606741573	**1.41573034**	-16.202247	16.7
**2007**	Forest	**64_07L1_**	1.606741573	**-20.5842697**	-2.2022472	17
**2007**	Forest	**64_07L1_**	1.606741573	**-6.58426966**	83.797753	16
**2007**	Forest	**67_07L1_**	-2.393258427	**-0.58426966**	18.797753	17
**2007**	Forest	**67_07L1_**	-2.393258427	**7.41573034**	21.797753	17.3
**2007**	Forest	**67_07L1_**	-2.393258427	**-2.58426966**	91.797753	16.3
**2007**	Forest	**80_07L1_**	-3.393258427	**2.41573034**	-37.202247	16.8
**2007**	Forest	**80_07L1_**	-3.393258427	**7.41573034**	-34.202247	16.6
**2007**	Forest	**80_07L1_**	-3.393258427	**10.4157303**	54.797753	16.1
**2007**	Forest	**84_07L1_**	1.606741573	**4.41573034**	-61.202247	15.9
**2007**	Forest	**84_07L1_**	1.606741573	**20.4157303**	-55.202247	15.4
**2007**	Forest	**84_07L1_**	1.606741573	**17.4157303**	-49.202247	16.3
**2007**	Forest	**92_07L1_**	-0.393258427	**8.41573034**	-24.202247	16.1
**2007**	Forest	**92_07L1_**	-0.393258427	**24.4157303**	-14.202247	16.3
**2007**	Forest	**92_07L1_**	-0.393258427	**17.4157303**	27.797753	15.9
**2007**	Forest	**96_07L1_**	1.606741573	**6.41573034**	-13.202247	15
**2007**	Forest	**96_07L1_**	1.606741573	**4.41573034**	0.7977528	15
**2007**	Forest	**96_07L1_**	1.606741573	**-8.58426966**	56.797753	16
**2007**	Forest	**107_07L1_**	-1.393258427	**-7.58426966**	-12.202247	16.3
**2007**	Forest	**107_07L1_**	-1.393258427	**-6.58426966**	-9.2022472	16
**2007**	Forest	**107_07L1_**	-1.393258427	**-10.5842697**	89.797753	17.5
**2007**	Forest	**108_07L1_**	0.606741573	**-10.5842697**	-18.202247	16
**2007**	Forest	**108_07L1_**	0.606741573	**-5.58426966**	72.797753	15.5
**2007**	Forest	**108_07L1_**	0.606741573	**-30.5842697**	90.797753	14.1
**2007**	Forest	**109_07L1_**	-0.393258427	**6.41573034**	-25.202247	14.5
**2007**	Forest	**109_07L1_**	-0.393258427	**-0.58426966**	-21.202247	15.5
**2007**	Forest	**109_07L1_**	-0.393258427	**-16.5842697**	-16.202247	14.5
**2007**	Forest	**150_07L1_**	19.60674157	**12.4157303**	-33.202247	17

**2007**	Forest	**150_07L1_**	19.60674157	**15.4157303**	-7.2022472	16
**2007**	Forest	**150_07L1_**	19.60674157	**14.4157303**	43.797753	16.8
**2007**	Forest	**163_07L1_**	-4.393258427	**15.4157303**	-94.202247	17.7
**2007**	Forest	**163_07L1_**	-4.393258427	**13.4157303**	-81.202247	18
**2007**	Forest	**163_07L1_**	-4.393258427	**6.41573034**	-79.202247	17.5
**2007**	Forest	**165_07L1_**	-3.393258427	**-12.5842697**	-19.202247	16.7
**2007**	Forest	**165_07L1_**	-3.393258427	**-16.5842697**	-11.202247	16
**2007**	Forest	**165_07L1_**	-3.393258427	**-1.58426966**	-10.202247	16.1
**2007**	Forest	**170_07L1_**	-2.393258427	**-34.5842697**	-42.202247	14
**2007**	Forest	**170_07L1_**	-2.393258427	**-4.58426966**	-22.202247	14.5
**2007**	Forest	**170_07L1_**	-2.393258427	**-26.5842697**	12.797753	14
**2007**	Forest	**174_07L1_**	4.606741573	**8.41573034**	-12.202247	18.3
**2007**	Forest	**174_07L1_**	4.606741573	**30.4157303**	0.7977528	19.4
**2007**	Forest	**174_07L1_**	4.606741573	**12.4157303**	13.797753	18.2
**2007**	Forest	**176_07L1_**	1.606741573	**5.41573034**	-39.202247	18.2
**2007**	Forest	**176_07L1_**	1.606741573	**10.4157303**	32.797753	17.4
**2007**	Forest	**176_07L1_**	1.606741573	**-2.58426966**	74.797753	17.6
**2007**	Forest	**8_07L2_**	1.606741573	**-0.58426966**	-31.202247	17.5
**2007**	Forest	**8_07L2_**	1.606741573	**-11.5842697**	0.7977528	18
**2007**	Forest	**8_07L2_**	1.606741573	**-13.5842697**	59.797753	17
**2007**	Forest	**14_07L2_**	3.606741573	**-0.58426966**	0.7977528	18
**2007**	Forest	**14_07L2_**	3.606741573	**-0.58426966**	24.797753	17.5
**2007**	Forest	**27_07L2_**	-1.393258427	**0.41573034**	-37.202247	12.8
**2007**	Forest	**27_07L2_**	-1.393258427	**3.41573034**	29.797753	13.8
**2007**	Forest	**27_07L2_**	-1.393258427	**11.4157303**	31.797753	15
**2007**	Forest	**28_07L2_**	-5.393258427	**-24.5842697**	-39.202247	11.1
**2007**	Forest	**28_07L2_**	-5.393258427	**-17.5842697**	50.797753	12.3
**2007**	Forest	**28_07L2_**	-5.393258427	**-23.5842697**	74.797753	10.2
**2007**	Forest	**33_07L2_**	-3.393258427	**16.4157303**	-73.202247	16.6
**2007**	Forest	**33_07L2_**	-3.393258427	**4.41573034**	-69.202247	15.7
**2007**	Forest	**33_07L2_**	-3.393258427	**3.41573034**	-56.202247	15.6
**2007**	Forest	**39_07L2_**	-3.393258427	**3.41573034**	-11.202247	15.5
**2007**	Forest	**39_07L2_**	-3.393258427	**9.41573034**	-7.2022472	16.8
**2007**	Forest	**39_07L2_**	-3.393258427	**14.4157303**	34.797753	15.9
**2007**	Forest	**48_07L2_**	-4.393258427	**-2.58426966**	-51.202247	17.2
**2007**	Forest	**48_07L2_**	-4.393258427	**2.41573034**	-14.202247	17.5
**2007**	Forest	**48_07L2_**	-4.393258427	**7.41573034**	85.797753	17.3
**2007**	Park	**10_07b_**	-4.978723404	**17.287234**	-83.678161	13.8
**2007**	Park	**10_07b_**	-4.978723404	**38.287234**	-59.678161	12.6
**2007**	Park	**10_07b_**	-4.978723404	**34.287234**	-29.678161	13.6
**2007**	Park	**13_07b_**	-6.978723404	**-9.71276596**	-63.678161	15
**2007**	Park	**13_07b_**	-6.978723404	**-11.712766**	15.321839	15.2
**2007**	Park	**13_07b_**	-6.978723404	**16.287234**	39.321839	16
**2007**	Park	**13_07b_**	-6.978723404	**-15.712766**	46.321839	13.8
**2007**	Park	**13_07b_**	-6.978723404	**-1.71276596**		15.1
**2007**	Park	**20_07b_**	-4.978723404	**-19.712766**	-49.678161	12.7
**2007**	Park	**20_07b_**	-4.978723404	**-3.71276596**	-16.678161	13.4
**2007**	Park	**20_07b_**	-4.978723404	**12.287234**	-6.6781609	15.1
**2007**	Park	**20_07b_**	-4.978723404	**-4.71276596**	41.321839	15.6
**2007**	Park	**20_07b_**	-4.978723404	**-14.712766**		13.4
**2007**	Park	**22_07b_**	2.021276596	**7.28723404**	-26.678161	15.2
**2007**	Park	**22_07b_**	2.021276596	**12.287234**	-9.6781609	15.1
**2007**	Park	**22_07b_**	2.021276596	**13.287234**	-1.6781609	14.2
**2007**	Park	**26_07b_**	2.021276596	**16.287234**	42.321839	16.5

**2007**	Park	**26_07b_**	2.021276596	**16.287234**	47.321839	14.5
**2007**	Park	**26_07b_**	2.021276596	**9.28723404**	64.321839	17
**2007**	Park	**32_07b_**	-0.978723404	**15.287234**	11.321839	17.1
**2007**	Park	**32_07b_**	-0.978723404	**13.287234**	12.321839	17.8
**2007**	Park	**32_07b_**	-0.978723404	**8.28723404**	48.321839	18.1
**2007**	Park	**39_07b_**	5.021276596	**2.28723404**	-22.678161	15.8
**2007**	Park	**39_07b_**	5.021276596	**7.28723404**	9.3218391	16.3
**2007**	Park	**39_07b_**	5.021276596	**-0.71276596**	82.321839	16.3
**2007**	Park	**44_07b_**	-0.978723404	**13.287234**	-67.678161	13.1
**2007**	Park	**44_07b_**	-0.978723404	**0.28723404**	-62.678161	13.1
**2007**	Park	**44_07b_**	-0.978723404	**0.28723404**	-25.678161	14
**2007**	Park	**46_07b_**	2.021276596	**-8.71276596**	-66.678161	13.7
**2007**	Park	**46_07b_**	2.021276596	**-2.71276596**	-43.678161	15.9
**2007**	Park	**46_07b_**	2.021276596	**-25.712766**	-37.678161	13.3
**2007**	Park	**57_07b_**	1.021276596	**1.28723404**	-42.678161	15
**2007**	Park	**57_07b_**	1.021276596	**22.287234**	-13.678161	12.3
**2007**	Park	**57_07b_**	1.021276596	**7.28723404**	119.32184	10.5
**2007**	Park	**58_07b_**	7.021276596	**-19.712766**	69.321839	15.3
**2007**	Park	**58_07b_**	7.021276596	**-33.712766**	139.32184	15.1
**2007**	Park	**58_07b_**	7.021276596	**-24.712766**	139.32184	14.1
**2007**	Park	**63_07b_**	1.021276596	**34.287234**	-82.678161	18.3
**2007**	Park	**63_07b_**	1.021276596	**24.287234**	-79.678161	17
**2007**	Park	**63_07b_**	1.021276596	**23.287234**	-35.678161	18
**2007**	Park	**64_07b_**	5.021276596	**2.28723404**	-16.678161	17.5
**2007**	Park	**64_07b_**	5.021276596	**12.287234**	-14.678161	18
**2007**	Park	**64_07b_**	5.021276596	**-8.71276596**	-1.6781609	18.3
**2007**	Park	**67_07b_**	6.021276596	**5.28723404**	-32.678161	16
**2007**	Park	**67_07b_**	6.021276596	**19.287234**	-31.678161	16
**2007**	Park	**67_07b_**	6.021276596	**6.28723404**	3.3218391	15
**2007**	Park	**71_07b_**	3.021276596	**6.28723404**	-45.678161	16.2
**2007**	Park	**71_07b_**	3.021276596	**11.287234**	32.321839	16.8
**2007**	Park	**71_07b_**	3.021276596	**14.287234**	43.321839	16.2
**2007**	Park	**73_07b_**	2.021276596	**-32.712766**	21.321839	15.6
**2007**	Park	**73_07b_**	2.021276596	**1.28723404**	46.321839	15.2
**2007**	Park	**73_07b_**	2.021276596	**2.28723404**	56.321839	16
**2007**	Park	**76_07b_**	3.021276596	**-4.71276596**	-15.678161	15.3
**2007**	Park	**76_07b_**	3.021276596	**-18.712766**	23.321839	14.5
**2007**	Park	**76_07b_**	3.021276596	**-14.712766**	39.321839	14.5
**2007**	Park	**76_07b_**	3.021276596	**-0.71276596**		15
**2007**	Park	**91_07b_**	6.021276596	**-26.712766**	-17.678161	17
**2007**	Park	**91_07b_**	6.021276596	**-46.712766**	35.321839	14.8
**2007**	Park	**91_07b_**	6.021276596	**-24.712766**	108.32184	15.8
**2007**	Park	**97_07b_**	-2.978723404	**-11.712766**	9.3218391	17.2
**2007**	Park	**97_07b_**	-2.978723404	**-17.712766**	14.321839	16.3
**2007**	Park	**97_07b_**	-2.978723404	**-9.71276596**	99.321839	17
**2007**	Park	**100_07b_**	0.021276596	**4.28723404**	-46.678161	13.8
**2007**	Park	**100_07b_**	0.021276596	**-5.71276596**	-35.678161	14.5
**2007**	Park	**100_07b_**	0.021276596	**-9.71276596**	-1.6781609	15.7
**2007**	Park	**106_07b_**	2.021276596	**-10.712766**	-56.678161	15.4
**2007**	Park	**106_07b_**	2.021276596	**-10.712766**	4.3218391	14.1
**2007**	Park	**106_07b_**	2.021276596	**8.28723404**	31.321839	14.3
**2007**	Park	**107_07b_**	0.021276596	**-9.71276596**		16.2
**2007**	Park	**107_07b_**	0.021276596	**-4.71276596**		14
**2007**	Park	**107_07b_**	0.021276596	**0.28723404**		13.8

**2007**	Park	**117_07b_**	-9.978723404	**-4.71276596**	-28.678161	15.4
**2007**	Park	**117_07b_**	-9.978723404	**-30.712766**	1.3218391	13.6
**2007**	Park	**117_07b_**	-9.978723404	**8.28723404**	8.3218391	15.7
**2007**	Park	**117_07b_**	-9.978723404	**-17.712766**	58.321839	14.4
**2007**	Park	**117_07b_**	-9.978723404	**-12.712766**		13.4
**2007**	Park	**127_07b_**	1.021276596	**-5.71276596**	-100.67816	15.4
**2007**	Park	**127_07b_**	1.021276596	**-9.71276596**	-65.678161	16.3
**2007**	Park	**127_07b_**	1.021276596	**-17.712766**	-44.678161	17.2
**2007**	Park	**92a_07b_**	2.021276596	**25.287234**	-82.678161	16
**2007**	Park	**92a_07b_**	2.021276596	**11.287234**	3.3218391	15.3
**2007**	Park	**92a_07b_**	2.021276596	**19.287234**	60.321839	15
**2007**	Park	**41_07z_**	3.021276596	**9.28723404**	-3.6781609	16
**2007**	Park	**41_07z_**	3.021276596	**19.287234**	9.3218391	16.2
**2007**	Park	**41_07z_**	3.021276596	**4.28723404**	23.321839	14.5
**2007**	Park	**46_07z_**	-2.978723404	**-2.71276596**	-62.678161	12.9
**2007**	Park	**46_07z_**	-2.978723404	**23.287234**	-22.678161	15.9
**2007**	Park	**46_07z_**	-2.978723404	**21.287234**	-7.6781609	16.4
**2007**	Park	**51_07z_**	-1.978723404	**-5.71276596**	-30.678161	14.7
**2007**	Park	**51_07z_**	-1.978723404	**-20.712766**	-25.678161	14.1
**2007**	Park	**51_07z_**	-1.978723404	**-34.712766**	2.3218391	14.3
**2007**	Park	**54_07z_**	-2.978723404	**5.28723404**	-14.678161	14
**2007**	Park	**54_07z_**	-2.978723404	**17.287234**	9.3218391	13.2
**2007**	Park	**54_07z_**	-2.978723404	**5.28723404**	65.321839	13.4
**2008**	Forest	**2_08L1_**	-0.90625	**-23.5**		13.6
**2008**	Forest	**2_08L1_**	-0.90625	**-16.5**		14
**2008**	Forest	**2_08L1_**	-0.90625	**-7.5**		14.4
**2008**	Forest	**17_08L1_**	-4.90625	**-56.5**		9.6
**2008**	Forest	**17_08L1_**	-4.90625	**-41.5**		10.8
**2008**	Forest	**17_08L1_**	-4.90625	**-39.5**		10
**2008**	Forest	**31_08L1_**	6.09375	**6.5**	-56.114286	18.4
**2008**	Forest	**31_08L1_**	6.09375	**-4.5**	-0.1142857	16.9
**2008**	Forest	**31_08L1_**	6.09375	**2.5**	22.885714	18
**2008**	Forest	**33_08L1_**	3.09375	**0.5**		17
**2008**	Forest	**33_08L1_**	3.09375	**5.5**		18.3
**2008**	Forest	**33_08L1_**	3.09375	**15.5**		16.3
**2008**	Forest	**43_08L1_**	-0.90625	**-19.5**		18.1
**2008**	Forest	**43_08L1_**	-0.90625	**-4.5**		17.8
**2008**	Forest	**43_08L1_**	-0.90625	**10.5**		16.3
**2008**	Forest	**54_08L1_**	6.09375	**-12.5**	-27.114286	15.6
**2008**	Forest	**54_08L1_**	6.09375	**-4.5**	-21.114286	15.2
**2008**	Forest	**54_08L1_**	6.09375	**-0.5**	36.885714	16.8
**2008**	Forest	**59_08L1_**	-2.90625	**20.5**	-34.114286	16.1
**2008**	Forest	**59_08L1_**	-2.90625	**14.5**	38.885714	15.5
**2008**	Forest	**59_08L1_**	-2.90625	**-3.5**	54.885714	16
**2008**	Forest	**60_08L1_**	2.09375	**-18.5**		13.6
**2008**	Forest	**60_08L1_**	2.09375	**-10.5**		14.2
**2008**	Forest	**60_08L1_**	2.09375	**-0.5**		13.1
**2008**	Forest	**62_08L1_**	6.09375	**-3.5**	-72.114286	15.4
**2008**	Forest	**62_08L1_**	6.09375	**-17.5**	-66.114286	16.4
**2008**	Forest	**62_08L1_**	6.09375	**-8.5**	43.885714	16.7
**2008**	Forest	**81_08L1_**	2.09375	**16.5**	-10.114286	15.6
**2008**	Forest	**81_08L1_**	2.09375	**8.5**	51.885714	16.2
**2008**	Forest	**81_08L1_**	2.09375	**20.5**	91.885714	16.2
**2008**	Forest	**86_08L1_**	2.09375	**10.5**		16.3

**2008**	Forest	**86_08L1_**	2.09375	**16.5**		17
**2008**	Forest	**86_08L1_**	2.09375	**21.5**		16
**2008**	Forest	**91_08L1_**	-2.90625	**20.5**	-53.114286	12.8
**2008**	Forest	**91_08L1_**	-2.90625	**7.5**	-22.114286	13.1
**2008**	Forest	**91_08L1_**	-2.90625	**4.5**	1.8857143	12.1
**2008**	Forest	**92_08L1_**	0.09375	**4.5**	-42.114286	15.5
**2008**	Forest	**92_08L1_**	0.09375	**-3.5**	41.885714	15.9
**2008**	Forest	**92_08L1_**	0.09375	**7.5**	46.885714	16.6
**2008**	Forest	**93_08L1_**	0.09375	**-16.5**	46.885714	8.8
**2008**	Forest	**93_08L1_**	0.09375	**-70.5**	131.88571	11.3
**2008**	Forest	**93_08L1_**	0.09375	**-32.5**		8
**2008**	Forest	**95_08L1_**	-1.90625	**4.5**	-86.114286	12.9
**2008**	Forest	**95_08L1_**	-1.90625	**-14.5**	-20.114286	11.4
**2008**	Forest	**95_08L1_**	-1.90625	**-3.5**	-18.114286	12.4
**2008**	Forest	**109_08L1_**	0.09375	**-8.5**		16.2
**2008**	Forest	**109_08L1_**	0.09375	**2.5**		15.9
**2008**	Forest	**109_08L1_**	0.09375	**12.5**		15.2
**2008**	Forest	**110_08L1_**	-1.90625	**-9.5**	-0.1142857	13.8
**2008**	Forest	**110_08L1_**	-1.90625	**-12.5**	51.885714	13.8
**2008**	Forest	**110_08L1_**	-1.90625	**-30.5**		13
**2008**	Forest	**138_08L1_**	-1.90625	**-5.5**	-27.114286	17
**2008**	Forest	**138_08L1_**	-1.90625	**-0.5**	-9.1142857	16.4
**2008**	Forest	**138_08L1_**	-1.90625	**-1.5**	61.885714	15.1
**2008**	Forest	**140_08L1_**	6.09375	**31.5**	-83.114286	17
**2008**	Forest	**140_08L1_**	6.09375	**36.5**	38.885714	17.7
**2008**	Forest	**140_08L1_**	6.09375	**31.5**	50.885714	17.1
**2008**	Forest	**150_08L1_**	1.09375	**-6.5**	-41.114286	11.8
**2008**	Forest	**150_08L1_**	1.09375	**8.5**	-40.114286	11.5
**2008**	Forest	**150_08L1_**	1.09375	**12.5**	-29.114286	12.8
**2008**	Forest	**154_08L1_**	5.09375	**-9.5**	4.8857143	14.2
**2008**	Forest	**154_08L1_**	5.09375	**-14.5**	23.885714	14
**2008**	Forest	**154_08L1_**	5.09375	**-18.5**	43.885714	14.4
**2008**	Forest	**166_08L1_**	-4.90625	**16.5**	-29.114286	15
**2008**	Forest	**166_08L1_**	-4.90625	**9.5**	6.8857143	15.6
**2008**	Forest	**166_08L1_**	-4.90625	**16.5**	17.885714	14.8
**2008**	Forest	**169_08L1_**	-1.90625	**11.5**	-26.114286	16.6
**2008**	Forest	**169_08L1_**	-1.90625	**-5.5**	-2.1142857	15.7
**2008**	Forest	**169_08L1_**	-1.90625	**-1.5**	3.8857143	15.9
**2008**	Forest	**184_08L1_**	8.09375	**11.5**	-42.114286	17.1
**2008**	Forest	**184_08L1_**	8.09375	**3.5**	-16.114286	16.7
**2008**	Forest	**184_08L1_**	8.09375	**3.5**	-0.1142857	18.5
**2008**	Forest	**3_08L2_**	0.09375	**5.5**	-8.1142857	16.1
**2008**	Forest	**3_08L2_**	0.09375	**11.5**	2.8857143	16.9
**2008**	Forest	**3_08L2_**	0.09375	**-1.5**	19.885714	14.7
**2008**	Forest	**4_08L2_**	-8.90625	**-1.5**	-64.114286	15.6
**2008**	Forest	**4_08L2_**	-8.90625	**10.5**	-34.114286	15.1
**2008**	Forest	**4_08L2_**	-8.90625	**18.5**	21.885714	15.4
**2008**	Forest	**31_08L2_**	7.09375	**23.5**	-12.114286	18
**2008**	Forest	**31_08L2_**	7.09375	**3.5**	7.8857143	18.4
**2008**	Forest	**31_08L2_**	7.09375	**12.5**	56.885714	17.8
**2008**	Forest	**32_08L2_**	-6.90625	**-3.5**	-51.114286	16
**2008**	Forest	**32_08L2_**	-6.90625	**-6.5**	-24.114286	16.9
**2008**	Forest	**32_08L2_**	-6.90625	**8.5**	-6.1142857	17.1
**2008**	Forest	**35_08L2_**	-3.90625	**14.5**	-60.114286	15.2

**2008**	Forest	**35_08L2_**	-3.90625	**3.5**	-30.114286	16.7
**2008**	Forest	**35_08L2_**	-3.90625	**22.5**	-20.114286	15.1
**2008**	Forest	**38_08L2_**	-5.90625	**0.5**	-44.114286	14.4
**2008**	Forest	**38_08L2_**	-5.90625	**-17.5**	-5.1142857	14.2
**2008**	Forest	**38_08L2_**	-5.90625	**-9.5**	49.885714	14.8
**2008**	Forest	**44_08L2_**	-2.90625	**13.5**	16.885714	16
**2008**	Forest	**44_08L2_**	-2.90625	**-5.5**	69.885714	16.8
**2008**	Forest	**44_08L2_**	-2.90625	**-5.5**	71.885714	15.9
**2008**	Forest	**49_08L2_**	-1.90625	**-9.5**		12.1
**2008**	Forest	**49_08L2_**	-1.90625	**19.5**		14.8
**2008**	Forest	**49_08L2_**	-1.90625	**24.5**		16.1
**2008**	Park	**2_08b_**	-0.215686275	**2.18954248**	1.0359712	17
**2008**	Park	**2_08b_**	-0.215686275	**4.18954248**	15.035971	17.4
**2008**	Park	**2_08b_**	-0.215686275	**11.1895425**	52.035971	18.3
**2008**	Park	**9_08b_**	-3.215686275	**7.18954248**	-12.964029	17.6
**2008**	Park	**9_08b_**	-3.215686275	**-0.81045752**	-7.9640288	19.1
**2008**	Park	**9_08b_**	-3.215686275	**5.18954248**	6.0359712	17.1
**2008**	Park	**16_08b_**	-4.215686275	**2.18954248**	-37.964029	16.2
**2008**	Park	**16_08b_**	-4.215686275	**5.18954248**	-4.9640288	16.2
**2008**	Park	**16_08b_**	-4.215686275	**5.18954248**	52.035971	15.8
**2008**	Park	**20_08b_**	0.784313725	**24.1895425**	-137.96403	13.1
**2008**	Park	**20_08b_**	0.784313725	**29.1895425**	-88.964029	13.4
**2008**	Park	**20_08b_**	0.784313725	**19.1895425**	-78.964029	13
**2008**	Park	**22_08b_**	2.784313725	**-10.8104575**	2.0359712	15.5
**2008**	Park	**22_08b_**	2.784313725	**-6.81045752**	5.0359712	15.1
**2008**	Park	**22_08b_**	2.784313725	**-2.81045752**	17.035971	16.3
**2008**	Park	**26_08b_**	1.784313725	**20.1895425**	-19.964029	15.3
**2008**	Park	**26_08b_**	1.784313725	**27.1895425**	20.035971	15.1
**2008**	Park	**26_08b_**	1.784313725	**17.1895425**	28.035971	14.7
**2008**	Park	**32_08b_**	4.784313725	**1.18954248**	1.0359712	14.3
**2008**	Park	**32_08b_**	4.784313725	**-19.8104575**	8.0359712	16
**2008**	Park	**32_08b_**	4.784313725	**-20.8104575**	51.035971	14.4
**2008**	Park	**37_08b_**	-2.215686275	**5.18954248**	-39.964029	17.2
**2008**	Park	**37_08b_**	-2.215686275	**7.18954248**	-9.9640288	16
**2008**	Park	**37_08b_**	-2.215686275	**-9.81045752**	1.0359712	15.5
**2008**	Park	**39_08b_**	2.784313725	**-34.8104575**	-3.9640288	12.4
**2008**	Park	**39_08b_**	2.784313725	**-35.8104575**		13.3
**2008**	Park	**39_08b_**	2.784313725	**-8.81045752**		14.4
**2008**	Park	**44_08b_**	2.784313725	**-12.8104575**	-59.964029	13.1
**2008**	Park	**44_08b_**	2.784313725	**-16.8104575**	3.0359712	15.1
**2008**	Park	**44_08b_**	2.784313725	**-20.8104575**	6.0359712	14.3
**2008**	Park	**27_08b_**	2.784313725	**-5.81045752**	-27.964029	15.2
**2008**	Park	**27_08b_**	2.784313725	**5.18954248**	6.0359712	15
**2008**	Park	**27_08b_**	2.784313725	**0.18954248**	31.035971	15.1
**2008**	Park	**48_08b_**	1.784313725	**-17.8104575**	-63.964029	15.7
**2008**	Park	**48_08b_**	1.784313725	**-8.81045752**	17.035971	17.1
**2008**	Park	**48_08b_**	1.784313725	**-3.81045752**		14.8
**2008**	Park	**49_08b_**	-1.215686275	**-4.81045752**	6.0359712	13.4
**2008**	Park	**49_08b_**	-1.215686275	**-1.81045752**	78.035971	12.2
**2008**	Park	**49_08b_**	-1.215686275	**7.18954248**	104.03597	12.5
**2008**	Park	**59_08b_**	0.784313725	**-5.81045752**	-23.964029	16.4
**2008**	Park	**59_08b_**	0.784313725	**2.18954248**	14.035971	15.8
**2008**	Park	**59_08b_**	0.784313725	**0.18954248**	15.035971	16.1
**2008**	Park	**61_08b_**	2.784313725	**3.18954248**	24.035971	15.3

**2008**	Park	**61_08b_**	2.784313725	**-3.81045752**	41.035971		15.5
**2008**	Park	**61_08b_**	2.784313725	**-0.81045752**	101.03597		13.5
**2008**	Park	**62_08b_**	3.784313725	**13.1895425**	17.035971		17.1
**2008**	Park	**62_08b_**	3.784313725	**11.1895425**	20.035971		16.4
**2008**	Park	**62_08b_**	3.784313725	**12.1895425**	34.035971		16.9
**2008**	Park	**64_08b_**	-6.215686275	**-7.81045752**	-0.9640288		16.4
**2008**	Park	**64_08b_**	-6.215686275	**16.1895425**	4.0359712		16.2
**2008**	Park	**64_08b_**	-6.215686275	**4.18954248**	15.035971		15.3
**2008**	Park	**67_08b_**	4.784313725	**-12.8104575**	-17.964029		16.4
**2008**	Park	**67_08b_**	4.784313725	**-4.81045752**	15.035971		16.6
**2008**	Park	**67_08b_**	4.784313725	**2.18954248**	72.035971		15.4
**2008**	Park	**69_08b_**	3.784313725	**-10.8104575**	-39.964029		12.3
**2008**	Park	**69_08b_**	3.784313725	**-8.81045752**	-38.964029		12.6
**2008**	Park	**69_08b_**	3.784313725	**-21.8104575**	25.035971		12.5
**2008**	Park	**72_08b_**	1.784313725	**7.18954248**	-59.964029		16.4
**2008**	Park	**72_08b_**	1.784313725	**2.18954248**	-54.964029		16.2
**2008**	Park	**72_08b_**	1.784313725	**6.18954248**	-32.964029		15.8
**2008**	Park	**74_08b_**	0.784313725	**7.18954248**	-51.964029		16.3
**2008**	Park	**74_08b_**	0.784313725	**17.1895425**	-8.9640288		16.4
**2008**	Park	**74_08b_**	0.784313725	**11.1895425**	28.035971		15.5
**2008**	Park	**81_08b_**	2.784313725	**19.1895425**	-40.964029		14.3
**2008**	Park	**81_08b_**	2.784313725	**24.1895425**	-11.964029		14.9
**2008**	Park	**81_08b_**	2.784313725	**5.18954248**	21.035971		16.3
**2008**	Park	**84_08b_**	-2.215686275	**13.1895425**	10.035971		16
**2008**	Park	**84_08b_**	-2.215686275	**9.18954248**	40.035971		16.1
**2008**	Park	**84_08b_**	-2.215686275	**-5.81045752**	59.035971		16.5
**2008**	Park	**87_08b_**	8.784313725	**-23.8104575**	0.0359712		14.6
**2008**	Park	**87_08b_**	8.784313725	**-17.8104575**	48.035971		15
**2008**	Park	**87_08b_**	8.784313725	**-24.8104575**	70.035971		14.6
**2008**	Park	**88_08b_**	-3.215686275	**-7.81045752**	19.035971		14.8
**2008**	Park	**88_08b_**	-3.215686275	**-10.8104575**	37.035971		15.4
**2008**	Park	**88_08b_**	-3.215686275	**-1.81045752**	50.035971		15.2
**2008**	Park	**91_08b_**	2.784313725	**-18.8104575**	-28.964029		13.5
**2008**	Park	**91_08b_**	2.784313725	**-29.8104575**	-5.9640288		13.8
**2008**	Park	**91_08b_**	2.784313725	**-25.8104575**	89.035971		14.5
**2008**	Park	**93_08b_**	1.784313725	**-34.8104575**	28.035971		16.6
**2008**	Park	**93_08b_**	1.784313725	**-39.8104575**	29.035971		16.1
**2008**	Park	**93_08b_**	1.784313725	**-26.8104575**	61.035971		15.4
**2008**	Park	**99_08b_**	-1.215686275	**-2.81045752**	-48.964029		14.8
**2008**	Park	**99_08b_**	-1.215686275	**9.18954248**	-31.964029		15.1
**2008**	Park	**99_08b_**	-1.215686275	**17.1895425**	16.035971		15.6
**2008**	Park	**100_08b_**	3.784313725	**-3.81045752**	-6.9640288		13.6
**2008**	Park	**100_08b_**	3.784313725	**-31.8104575**	25.035971		11.9
**2008**	Park	**100_08b_**	3.784313725	**-5.81045752**	75.035971		14.4
**2008**	Park	**108_08b_**	0.784313725	**-9.81045752**			17.3
**2008**	Park	**108_08b_**	0.784313725	**5.18954248**			18.6
**2008**	Park	**108_08b_**	0.784313725	**17.1895425**			15.9
**2008**	Park	**112_08b_**	-2.215686275	**28.1895425**	-70.964029		15.4
**2008**	Park	**112_08b_**	-2.215686275	**26.1895425**	-55.964029		14.9
**2008**	Park	**112_08b_**	-2.215686275	**24.1895425**	-53.964029		16.2
**2008**	Park	**116_08b_**	-3.215686275	**-14.8104575**	24.035971		14.6
**2008**	Park	**116_08b_**	-3.215686275	**-21.8104575**	42.035971		14.7
**2008**	Park	**116_08b_**	-3.215686275	**-30.8104575**	72.035971		14.1
**2008**	Park	**124_08b_**	-2.215686275	**-4.81045752**	-26.964029		16.2

**2008**	Park	**124_08b_**	-2.215686275	**-13.8104575**	30.035971	15.6
**2008**	Park	**124_08b_**	-2.215686275	**-3.81045752**	66.035971	15
**2008**	Park	**125_08b_**	-1.215686275	**2.18954248**	-38.964029	16
**2008**	Park	**125_08b_**	-1.215686275	**7.18954248**	-33.964029	16
**2008**	Park	**125_08b_**	-1.215686275	**5.18954248**	-32.964029	16.7
**2008**	Park	**127_08b_**	-4.215686275	**47.1895425**	-97.964029	16
**2008**	Park	**127_08b_**	-4.215686275	**27.1895425**	-68.964029	15.4
**2008**	Park	**127_08b_**	-4.215686275	**25.1895425**	-62.964029	15.7
**2008**	Park	**56a_08b_**	0.784313725	**16.1895425**	35.035971	14.9
**2008**	Park	**56a_08b_**	0.784313725	**17.1895425**	46.035971	15.1
**2008**	Park	**56a_08b_**	0.784313725	**24.1895425**	82.035971	15.1
**2008**	Park	**62a_08b_**	-2.215686275	**0.18954248**	-10.964029	17.7
**2008**	Park	**62a_08b_**	-2.215686275	**12.1895425**	-4.9640288	16.1
**2008**	Park	**62a_08b_**	-2.215686275	**-3.81045752**	61.035971	16.8
**2008**	Park	**9_08z_**	-6.215686275	**-21.8104575**	-12.964029	15.6
**2008**	Park	**9_08z_**	-6.215686275	**-18.8104575**	-0.9640288	15.4
**2008**	Park	**9_08z_**	-6.215686275	**-19.8104575**	24.035971	16.2
**2008**	Park	**11_08z_**	-4.215686275	**-35.8104575**		14
**2008**	Park	**11_08z_**	-4.215686275	**-27.8104575**		13.5
**2008**	Park	**11_08z_**	-4.215686275	**-10.8104575**		14.4
**2008**	Park	**14_08z_**	-3.215686275	**-36.8104575**	2.0359712	15.2
**2008**	Park	**14_08z_**	-3.215686275	**-31.8104575**	6.0359712	13.9
**2008**	Park	**14_08z_**	-3.215686275	**-26.8104575**		15.5
**2008**	Park	**18_08z_**	5.784313725	**-11.8104575**	-75.964029	14.2
**2008**	Park	**18_08z_**	5.784313725	**-9.81045752**	-50.964029	14.4
**2008**	Park	**18_08z_**	5.784313725	**-0.81045752**	-44.964029	14.9
**2008**	Park	**22_08z_**	-4.215686275	**-3.81045752**	-54.964029	15.8
**2008**	Park	**22_08z_**	-4.215686275	**-14.8104575**	9.0359712	17.1
**2008**	Park	**22_08z_**	-4.215686275	**-10.8104575**	31.035971	15
**2008**	Park	**26_08z_**	2.784313725	**25.1895425**	-70.964029	16.5
**2008**	Park	**26_08z_**	2.784313725	**21.1895425**	-33.964029	16.6
**2008**	Park	**26_08z_**	2.784313725	**6.18954248**	13.035971	16.3
**2008**	Park	**40_08z_**	-2.215686275	**-19.8104575**	46.035971	14.8
**2008**	Park	**40_08z_**	-2.215686275	**-4.81045752**	89.035971	13.7
**2008**	Park	**40_08z_**	-2.215686275	**-10.8104575**		13.6
**2008**	Park	**41_08z_**	0.784313725	**25.1895425**	6.0359712	14.9
**2008**	Park	**41_08z_**	0.784313725	**25.1895425**	7.0359712	15.8
**2008**	Park	**41_08z_**	0.784313725	**40.1895425**	20.035971	15.9
**2008**	Park	**45_08z_**	-6.215686275	**-14.8104575**	7.0359712	15.2
**2008**	Park	**45_08z_**	-6.215686275	**-13.8104575**	18.035971	13.2
**2008**	Park	**45_08z_**	-6.215686275	**2.18954248**		15.2
**2008**	Park	**46_08z_**	-3.215686275	**23.1895425**	-52.964029	16.9
**2008**	Park	**46_08z_**	-3.215686275	**17.1895425**	-50.964029	16.9
**2008**	Park	**46_08z_**	-3.215686275	**15.1895425**	7.0359712	16.6
**2008**	Park	**47_08z_**	1.784313725	**56.1895425**	-23.964029	17
**2008**	Park	**47_08z_**	1.784313725	**33.1895425**	-7.9640288	17.9
**2008**	Park	**47_08z_**	1.784313725	**-3.81045752**	22.035971	17.2
**2008**	Park	**50_08z_**	-0.215686275	**4.18954248**	-78.964029	17.9
**2008**	Park	**50_08z_**	-0.215686275	**12.1895425**	31.035971	16.5
**2008**	Park	**50_08z_**	-0.215686275	**-2.81045752**		17
**2008**	Park	**60_08z_**	-4.215686275	**6.18954248**	-83.964029	14
**2008**	Park	**60_08z_**	-4.215686275	**-8.81045752**	7.0359712	16.7
**2008**	Park	**60_08z_**	-4.215686275	**3.18954248**	16.035971	16.5
**2008**	Park	**29a_08z_**	1.784313725	**26.1895425**	-80.964029	16.3

**2008**	Park	**29a_08z_**	1.784313725	**22.1895425**	-46.964029	16.9
**2008**	Park	**29a_08z_**	1.784313725	**31.1895425**		18
**2009**	Forest	**15_09L1_**	-5.670731707	**-19.463**		14.9
**2009**	Forest	**15_09L1_**	-5.670731707	**-18.463**		14.5
**2009**	Forest	**15_09L1_**	-3.670731707	**-1.463**		17.2
**2009**	Forest	**16_09L1_**	-3.670731707	**1.537**		17.6
**2009**	Forest	**16_09L1_**	-3.670731707	**7.537**		17.7
**2009**	Forest	**18_09L1_**	-0.670731707	**0.537**	-39.457	17.7
**2009**	Forest	**18_09L1_**	-0.670731707	**-7.463**	-27.457	17.1
**2009**	Forest	**18_09L1_**	-0.670731707	**-2.463**	44.543	18.4
**2009**	Forest	**19_09L1_**	1.329268293	**-2.463**	6.543	18.5
**2009**	Forest	**19_09L1_**	1.329268293	**-0.463**	15.543	17.4
**2009**	Forest	**19_09L1_**	1.329268293	**-9.463**		16.8
**2009**	Forest	**24_09L1_**	-3.670731707	**7.537**		15.1
**2009**	Forest	**24_09L1_**	-3.670731707	**7.537**		17.5
**2009**	Forest	**24_09L1_**	-3.670731707	**12.537**		16.1
**2009**	Forest	**32_09L1_**	0.329268293	**13.537**	-6.457	15.7
**2009**	Forest	**32_09L1_**	0.329268293	**11.537**	57.543	17.5
**2009**	Forest	**32_09L1_**	0.329268293	**13.537**	73.543	16.7
**2009**	Forest	**42_09L1_**	0.329268293	**12.537**	-74.457	16.9
**2009**	Forest	**42_09L1_**	0.329268293	**7.537**	-46.457	16.7
**2009**	Forest	**49_09L1_**	-0.670731707	**-2.463**	-26.457	16.3
**2009**	Forest	**49_09L1_**	-0.670731707	**12.537**	8.543	17.7
**2009**	Forest	**49_09L1_**	-0.670731707	**9.537**	35.543	16.5
**2009**	Forest	**63_09L1_**	-0.670731707	**1.537**		17.5
**2009**	Forest	**63_09L1_**	-0.670731707	**18.537**		17.4
**2009**	Forest	**63_09L1_**	-0.670731707	**22.537**		18.5
**2009**	Forest	**69_09L1_**	-2.670731707	**0.537**	24.543	
**2009**	Forest	**69_09L1_**	-2.670731707	**8.537**	47.543	17
**2009**	Forest	**69_09L1_**	-2.670731707	**27.537**	100.543	15.2
**2009**	Forest	**86_09L1_**	4.329268293	**-14.463**		15.9
**2009**	Forest	**86_09L1_**	4.329268293	**-10.463**		16.7
**2009**	Forest	**86_09L1_**	4.329268293	**1.537**		16.6
**2009**	Forest	**96_09L1_**	7.329268293	**-24.463**	-23.457	15.7
**2009**	Forest	**96_09L1_**	7.329268293	**-31.463**		17.2
**2009**	Forest	**96_09L1_**	7.329268293	**-27.463**		16.5
**2009**	Forest	**107_09L1_**	-0.670731707	**-2.463**		16.9
**2009**	Forest	**107_09L1_**	-0.670731707	**-0.463**		17
**2009**	Forest	**107_09L1_**	-0.670731707	**16.537**		15.2
**2009**	Forest	**109_09L1_**	-1.670731707	**7.537**	-9.457	16.4
**2009**	Forest	**109_09L1_**	-1.670731707	**12.537**	22.543	17.8
**2009**	Forest	**109_09L1_**	-1.670731707	**2.537**	39.543	17
**2009**	Forest	**141_09L1_**	-0.670731707	**16.537**	-4.457	16.4
**2009**	Forest	**141_09L1_**	-0.670731707	**12.537**	32.543	17.7
**2009**	Forest	**141_09L1_**	-0.670731707	**-2.463**	71.543	18.2
**2009**	Forest	**147_09L1_**	1.329268293	**-6.463**		15.1
**2009**	Forest	**147_09L1_**	1.329268293	**-3.463**		15.7
**2009**	Forest	**147_09L1_**	1.329268293	**5.537**		15.8
**2009**	Forest	**148_09L1_**	0.329268293	**11.537**	-36.457	18.9
**2009**	Forest	**148_09L1_**	0.329268293	**-9.463**	7.543	19.1
**2009**	Forest	**148_09L1_**	0.329268293	**13.537**	-53.457	18.1
**2009**	Forest	**167_09L1_**	1.329268293	**25.537**	-38.457	17.2
**2009**	Forest	**167_09L1_**	1.329268293	**4.537**	-10.457	16.9
**2009**	Forest	**167_09L1_**	1.329268293	**4.537**	57.543	16.5

**2009**	Forest	**10_09L2_**	-5.670731707	**-25.463**		20
**2009**	Forest	**10_09L2_**	-5.670731707	**-17.463**		19.4
**2009**	Forest	**10_09L2_**	-5.670731707	**-14.463**		20
**2009**	Forest	**13_09L2_**	4.329268293	**-9.463**	-97.457	17.2
**2009**	Forest	**13_09L2_**	4.329268293	**9.537**	-41.457	17.5
**2009**	Forest	**13_09L2_**	4.329268293	**-6.463**	37.543	15.9
**2009**	Forest	**14_09L2_**	4.329268293	**-3.463**		16.5
**2009**	Forest	**14_09L2_**	4.329268293	**7.537**		18.2
**2009**	Forest	**14_09L2_**	4.329268293	**11.537**		17.6
**2009**	Forest	**19_09L2_**	7.329268293	**-21.463**	-3.457	15.6
**2009**	Forest	**19_09L2_**	7.329268293	**-27.463**	7.543	15.7
**2009**	Forest	**19_09L2_**	7.329268293	**-20.463**	23.543	15.7
**2009**	Forest	**31_09L2_**	-2.670731707	**-1.463**	2.543	15.2
**2009**	Forest	**31_09L2_**	-2.670731707	**-8.463**	12.543	15.3
**2009**	Forest	**31_09L2_**	-2.670731707	**-9.463**	55.543	15.7
**2009**	Forest	**37_09L2_**	4.329268293	**0.537**	-28.457	16.5
**2009**	Forest	**37_09L2_**	4.329268293	**14.537**	55.543	18
**2009**	Forest	**37_09L2_**	4.329268293	**8.537**		18.7
**2009**	Forest	**38_09L2_**	-1.670731707	**-10.463**		16.3
**2009**	Forest	**38_09L2_**	-1.670731707	**-6.463**		17.9
**2009**	Forest	**38_09L2_**	-1.670731707	**3.537**		16.8
**2009**	Forest	**41_09L2_**	1.329268293	**0.537**	-81.457	18.5
**2009**	Forest	**41_09L2_**	1.329268293	**-11.463**	-67.457	17.6
**2009**	Forest	**41_09L2_**	1.329268293	**1.537**	-24.457	17.6
**2009**	Forest	**44_09L2_**	-7.670731707	**-19.463**		17.2
**2009**	Forest	**44_09L2_**	-7.670731707	**-11.463**		17.9
**2009**	Forest	**44_09L2_**	-7.670731707	**-7.463**		16.3
**2009**	Forest	**48_09L2_**	-1.670731707	**8.537**	-59.457	17.4
**2009**	Forest	**48_09L2_**	-1.670731707	**20.537**	-27.457	18.3
**2009**	Forest	**48_09L2_**	-1.670731707	**10.537**	-12.457	18.3
**2009**	Park	**4_09b_**	-2.858208955	**4.41044776**	-34.992	16.7
**2009**	Park	**4_09b_**	-2.858208955	**25.4104478**	-8.992	17.4
**2009**	Park	**4_09b_**	-2.858208955	**24.4104478**	0.008	16.2
**2009**	Park	**10_09b_**	-5.858208955	**-7.58955224**	-27.992	15.1
**2009**	Park	**10_09b_**	-5.858208955	**-13.5895522**	81.008	15
**2009**	Park	**10_09b_**	-5.858208955	**-13.5895522**	143.008	13
**2009**	Park	**16_09b_**	-3.858208955	**3.41044776**	-36.992	16.7
**2009**	Park	**16_09b_**	-3.858208955	**-9.58955224**	-18.992	16.4
**2009**	Park	**16_09b_**	-3.858208955	**-13.5895522**	-1.992	15.6
**2009**	Park	**23_09b_**	5.141791045	**1.41044776**	-38.992	13.5
**2009**	Park	**23_09b_**	5.141791045	**-9.58955224**	3.008	14.8
**2009**	Park	**23_09b_**	5.141791045	**-19.5895522**	40.008	15.5
**2009**	Park	**30_09b_**	1.141791045	**14.4104478**	-30.992	14.4
**2009**	Park	**30_09b_**	1.141791045	**-0.58955224**	-28.992	15.1
**2009**	Park	**30_09b_**	1.141791045	**33.4104478**	-25.992	14.1
**2009**	Park	**34_09b_**	3.141791045	**-26.5895522**	34.008	15.6
**2009**	Park	**34_09b_**	3.141791045	**-19.5895522**	42.008	16
**2009**	Park	**34_09b_**	3.141791045	**20.4104478**	56.008	16.2
**2009**	Park	**40_09b_**	1.141791045	**43.4104478**	-61.992	14.8
**2009**	Park	**40_09b_**	1.141791045	**16.4104478**	-45.992	15.6
**2009**	Park	**40_09b_**	1.141791045	**0.41044776**	-20.992	16.5
**2009**	Park	**44_09b_**	-0.858208955	**3.41044776**	-15.992	16
**2009**	Park	**44_09b_**	-0.858208955	**-0.58955224**	13.008	17
**2009**	Park	**44_09b_**	-0.858208955	**5.41044776**	49.008	17.8

**2009**	Park	**50_09b_**	6.141791045	**-9.58955224**	-74.992	13.9
**2009**	Park	**50_09b_**	6.141791045	**-6.58955224**	-65.992	12.7
**2009**	Park	**50_09b_**	6.141791045	**-14.5895522**	-58.992	13
**2009**	Park	**53_09b_**	-0.858208955	**5.41044776**	-52.992	16.6
**2009**	Park	**53_09b_**	-0.858208955	**9.41044776**	52.008	17.1
**2009**	Park	**53_09b_**	-0.858208955	**17.4104478**	104.008	17.6
**2009**	Park	**59_09b_**	2.141791045	**17.4104478**	-81.992	15.7
**2009**	Park	**59_09b_**	2.141791045	**11.4104478**	4.008	15.8
**2009**	Park	**59_09b_**	2.141791045	**-8.58955224**	11.008	16.5
**2009**	Park	**60_09b_**	2.141791045	**2.41044776**	22.008	15.5
**2009**	Park	**60_09b_**	2.141791045	**-14.5895522**	51.008	15.7
**2009**	Park	**60_09b_**	2.141791045	**-6.58955224**	88.008	15.1
**2009**	Park	**64_09b_**	9.141791045	**-7.58955224**	12.008	14.8
**2009**	Park	**64_09b_**	9.141791045	**-23.5895522**	15.008	15.1
**2009**	Park	**64_09b_**	9.141791045	**-1.58955224**	99.008	15.5
**2009**	Park	**69_09b_**	1.141791045	**-1.58955224**	-8.992	16.1
**2009**	Park	**69_09b_**	1.141791045	**-6.58955224**	77.008	15.8
**2009**	Park	**69_09b_**	1.141791045	**-19.5895522**	122.008	16.7
**2009**	Park	**72_09b_**	3.141791045	**8.41044776**	-83.992	12.6
**2009**	Park	**72_09b_**	3.141791045	**15.4104478**	-52.992	12.5
**2009**	Park	**72_09b_**	3.141791045	**12.4104478**	48.008	12.5
**2009**	Park	**73_09b_**	-2.858208955	**10.4104478**	-30.992	15.5
**2009**	Park	**73_09b_**	-2.858208955	**10.4104478**	6.008	14.7
**2009**	Park	**73_09b_**	-2.858208955	**24.4104478**	15.008	14
**2009**	Park	**81_09b_**	-1.858208955	**-22.5895522**	-51.992	12.3
**2009**	Park	**81_09b_**	-1.858208955	**-19.5895522**	-0.992	12.7
**2009**	Park	**81_09b_**	-1.858208955	**-23.5895522**	6.008	13.8
**2009**	Park	**84_09b_**	-3.858208955	**-5.58955224**	-38.992	14.1
**2009**	Park	**84_09b_**	-3.858208955	**11.4104478**	59.008	15.5
**2009**	Park	**84_09b_**	-3.858208955	**-12.5895522**	130.008	14.3
**2009**	Park	**88_09b_**	1.141791045	**1.41044776**	2.008	16.1
**2009**	Park	**88_09b_**	1.141791045	**-0.58955224**	3.008	16.5
**2009**	Park	**88_09b_**	1.141791045	**14.4104478**	47.008	15.9
**2009**	Park	**93_09b_**	-4.858208955	**-20.5895522**	-49.992	16
**2009**	Park	**93_09b_**	-4.858208955	**-2.58955224**	-21.992	17.9
**2009**	Park	**93_09b_**	-4.858208955	**-2.58955224**	-10.992	15.4
**2009**	Park	**94_09b_**	2.141791045	**6.41044776**	-16.992	16.4
**2009**	Park	**94_09b_**	2.141791045	**7.41044776**	-8.992	16.5
**2009**	Park	**94_09b_**	2.141791045	**-2.58955224**	17.008	15.7
**2009**	Park	**99_09b_**	-1.858208955	**2.41044776**	-49.992	14.7
**2009**	Park	**99_09b_**	-1.858208955	**-0.58955224**	15.008	14.3
**2009**	Park	**99_09b_**	-1.858208955	**-18.5895522**	24.008	14.9
**2009**	Park	**100_09b_**	0.141791045	**-11.5895522**	-54.992	15.7
**2009**	Park	**100_09b_**	0.141791045	**-2.58955224**	-35.992	14.8
**2009**	Park	**100_09b_**	0.141791045	**-21.5895522**	92.008	16
**2009**	Park	**103_09b_**	0.141791045	**11.4104478**	-3.992	14.6
**2009**	Park	**103_09b_**	0.141791045	**2.41044776**	37.008	14.1
**2009**	Park	**103_09b_**	0.141791045	**-2.58955224**	83.008	15.8
**2009**	Park	**121_09b_**	-1.858208955	**1.41044776**	-56.992	14.4
**2009**	Park	**121_09b_**	-1.858208955	**2.41044776**	-36.992	14.4
**2009**	Park	**121_09b_**	-1.858208955	**2.41044776**	21.008	14.9
**2009**	Park	**123_09b_**	-3.858208955	**9.41044776**	-24.992	15.3
**2009**	Park	**123_09b_**	-3.858208955	**16.4104478**	-16.992	16.1
**2009**	Park	**123_09b_**	-3.858208955	**20.4104478**	-2.992	14.4

**2009**	Park	**126_09b_**	-4.858208955	**2.41044776**	-13.992	15.2
**2009**	Park	**126_09b_**	-4.858208955	**2.41044776**	32.008	13.9
**2009**	Park	**126_09b_**	-4.858208955	**2.41044776**		14.6
**2009**	Park	**128_09b_**	-3.858208955	**12.4104478**	-25.992	16.9
**2009**	Park	**128_09b_**	-3.858208955	**7.41044776**	-20.992	16.7
**2009**	Park	**128_09b_**	-3.858208955	**-2.58955224**	1.008	15.3
**2009**	Park	**56a_09b_**	-1.858208955	**16.4104478**	-26.992	18.7
**2009**	Park	**56a_09b_**	-1.858208955	**20.4104478**	53.008	18.4
**2009**	Park	**62a_09b_**	6.141791045	**-38.5895522**	-8.992	16.4
**2009**	Park	**62a_09b_**	6.141791045	**-31.5895522**	16.008	16.1
**2009**	Park	**62a_09b_**	6.141791045	**-31.5895522**	34.008	16.1
**2009**	Park	**92a_09b_**	4.141791045	**14.4104478**	-7.992	17.4
**2009**	Park	**92a_09b_**	4.141791045	**1.41044776**	33.008	16.3
**2009**	Park	**92a_09b_**	4.141791045	**-4.58955224**	54.008	18.1
**2009**	Park	**5_09z_**	3.141791045	**-13.5895522**	-44.992	12.9
**2009**	Park	**1_09z_**	3.141791045	**-18.5895522**	-19.992	14
**2009**	Park	**5_09z_**	3.141791045	**-22.5895522**	19.008	11.5
**2009**	Park	**11_09z_**	-1.858208955	**7.41044776**	-76.992	17.3
**2009**	Park	**11_09z_**	-1.858208955	**-7.58955224**	-30.992	17.8
**2009**	Park	**11_09z_**	-1.858208955	**4.41044776**	-27.992	18.2
**2009**	Park	**13_09z_**	-0.858208955	**-6.58955224**		14.4
**2009**	Park	**13_09z_**	-0.858208955	**-5.58955224**		14.2
**2009**	Park	**13_09z_**	-0.858208955	**-5.58955224**		14
**2009**	Park	**17_09z_**	3.141791045	**-15.5895522**	-65.992	16.8
**2009**	Park	**17_09z_**	3.141791045	**18.4104478**	-62.992	17.6
**2009**	Park	**17_09z_**	3.141791045	**-15.5895522**	-4.992	16.2
**2009**	Park	**22_09z_**	-2.858208955	**7.41044776**	-44.992	17.7
**2009**	Park	**22_09z_**	-2.858208955	**2.41044776**	52.008	17.2
**2009**	Park	**22_09z_**	-2.858208955	**15.4104478**	102.008	17.7
**2009**	Park	**36_09z_**	-3.858208955	**-16.5895522**	-20.992	15.9
**2009**	Park	**36_09z_**	-3.858208955	**2.41044776**	-15.992	16
**2009**	Park	**36_09z_**	-3.858208955	**-0.58955224**		15.6
**2009**	Park	**39_09z_**	-1.858208955	**1.41044776**	-38.992	16.1
**2009**	Park	**39_09z_**	-1.858208955	**9.41044776**	-22.992	18.3
**2009**	Park	**39_09z_**	-1.858208955	**14.4104478**	47.008	16.9
**2009**	Park	**45_09z_**	-0.858208955	**7.41044776**	2.008	15.6
**2009**	Park	**45_09z_**	-0.858208955	**11.4104478**	3.008	17.7
**2009**	Park	**45_09z_**	-0.858208955	**12.4104478**	17.008	16.3
**2009**	Park	**46_09z_**	-3.858208955	**6.41044776**	-42.992	17.8
**2009**	Park	**46_09z_**	-3.858208955	**6.41044776**	-8.992	17.8
**2009**	Park	**46_09z_**	-3.858208955	**-16.5895522**	18.008	17.8
**2009**	Park	**48_09z_**	-3.858208955	**-3.58955224**	55.008	17.9
**2009**	Park	**48_09z_**	-3.858208955	**-2.58955224**	92.008	17.3
**2009**	Park	**48_09z_**	-3.858208955	**-10.5895522**		17.2
**2009**	Park	**55_09z_**	2.141791045	**-2.58955224**	-23.992	14.7
**2009**	Park	**55_09z_**	2.141791045	**-7.58955224**	66.008	13.8
**2009**	Park	**55_09z_**	2.141791045	**-4.58955224**		14.8
**2009**	Park	**58_09z_**	11.14179104	**13.4104478**	-80.992	16.2
**2009**	Park	**58_09z_**	11.14179104	**0.41044776**	-70.992	15.1
**2009**	Park	**58_09z_**	11.14179104	**25.4104478**	-14.992	15.7
**2009**	Park	**60_09z_**	-4.858208955	**-19.5895522**	-26.992	13.9
**2009**	Park	**60_09z_**	-4.858208955	**-24.5895522**		15.2
**2009**	Park	**60_09z_**	-4.858208955	**2.41044776**		15.3
**2009**	Park	**29a_09z_**	2.141791045	**17.4104478**	-69.992	17.2

**2009**	Park	**29a_09z_**	2.141791045	**19.4104478**	-49.992	15.2
**2009**	Park	**29a_09z_**	2.141791045	**19.4104478**	-48.992	15.1
**2010**	Forest	**36_10L1_**	1.714285714	**16.2619048**	-51	13.5
**2010**	Forest	**36_10L1_**	1.714285714	**6.26190476**	-14	13.6
**2010**	Forest	**36_10L1_**	1.714285714	**17.2619048**	43	14.2
**2010**	Forest	**68_10L1_**	4.714285714	**1.26190476**	12	12
**2010**	Forest	**68_10L1_**	4.714285714	**-10.7380952**	51	11.6
**2010**	Forest	**68_10L1_**	4.714285714	**-3.73809524**	65	11
**2010**	Forest	**82_10L1_**	0.714285714	**17.2619048**	-52	16.8
**2010**	Forest	**82_10L1_**	0.714285714	**12.2619048**	-15	16.8
**2010**	Forest	**82_10L1_**	0.714285714	**4.26190476**	62	17.3
**2010**	Forest	**108_10L1_**	-4.285714286	**-8.73809524**	-59	15.6
**2010**	Forest	**108_10L1_**	-4.285714286	**-3.73809524**	11	14.2
**2010**	Forest	**108_10L1_**	-4.285714286	**-6.73809524**	29	14.9
**2010**	Forest	**109_10L1_**	-5.285714286	**-1.73809524**	-16	17.2
**2010**	Forest	**109_10L1_**	-5.285714286	**-1.73809524**	5	15.8
**2010**	Forest	**109_10L1_**	-5.285714286	**5.26190476**	7	15.8
**2010**	Forest	**110_10L1_**	-0.285714286	**14.2619048**	-85	12.2
**2010**	Forest	**110_10L1_**	-0.285714286	**-2.73809524**	-47	14.7
**2010**	Forest	**110_10L1_**	-0.285714286	**5.26190476**	-47	15.2
**2010**	Forest	**117_10L1_**	-3.285714286	**-32.7380952**	-37	13.5
**2010**	Forest	**117_10L1_**	-3.285714286	**-14.7380952**	16	13.7
**2010**	Forest	**117_10L1_**	-3.285714286	**-18.7380952**	18	14.8
**2010**	Forest	**149_10L1_**	2.714285714	**-1.73809524**	-44	14.9
**2010**	Forest	**149_10L1_**	2.714285714	**7.26190476**	-21	14.8
**2010**	Forest	**149_10L1_**	2.714285714	**4.26190476**	14	15.1
**2010**	Forest	**171_10L1_**	0.714285714	**-3.73809524**	10	13.8
**2010**	Forest	**171_10L1_**	0.714285714	**9.26190476**	26	12.5
**2010**	Forest	**171_10L1_**	0.714285714	**0.26190476**	29	13.1
**2010**	Forest	**308_10L1_**	0.714285714	**10.2619048**	-1	15.7
**2010**	Forest	**308_10L1_**	0.714285714	**15.2619048**	14	15.5
**2010**	Forest	**308_10L1_**	0.714285714	**22.2619048**	40	15.5
**2010**	Forest	**9_10L2_**	-2.285714286	**-3.73809524**	-46	11.7
**2010**	Forest	**9_10L2_**	-2.285714286	**-1.73809524**	-34	13.7
**2010**	Forest	**9_10L2_**	-2.285714286	**-4.73809524**	-14	11.4
**2010**	Forest	**14_10L2_**	3.714285714	**-17.7380952**	-27	12.7
**2010**	Forest	**14_10L2_**	3.714285714	**-17.7380952**	-18	11.4
**2010**	Forest	**14_10L2_**	3.714285714	**-18.7380952**	-12	12.9
**2010**	Forest	**38_10L2_**	-0.285714286	**7.26190476**	-65	14.7
**2010**	Forest	**38_10L2_**	-0.285714286	**14.2619048**	8	16.5
**2010**	Forest	**38_10L2_**	-0.285714286	**6.26190476**	62	16.5
**2010**	Forest	**44_10L2_**	0.714285714	**-3.73809524**	13	14.8
**2010**	Forest	**44_10L2_**	0.714285714	**-20.7380952**	77	13.8
**2010**	Forest	**44_10L2_**	0.714285714	**4.26190476**	93	14.9
**2010**	Park	**3_10b_**	-4.132231405	**-18.2231405**	31.134454	16.5
**2010**	Park	**3_10b_**	-4.132231405	**-19.2231405**		18.1
**2010**	Park	**4_10b_**	-0.132231405	**-10.2231405**	-91.865546	13.4
**2010**	Park	**3_10b_**	-0.132231405	**4.7768595**	-85.865546	15.9
**2010**	Park	**4_10b_**	-0.132231405	**-32.2231405**	-80.865546	9.4
**2010**	Park	**10_10b_**	-1.132231405	**25.7768595**	-82.865546	11.6
**2010**	Park	**10_10b_**	-1.132231405	**30.7768595**	2.1344538	14.2
**2010**	Park	**10_10b_**	-1.132231405	**-2.2231405**	31.134454	11.5
**2010**	Park	**13_10b_**	-4.132231405	**20.7768595**	-46.865546	12.7
**2010**	Park	**13_10b_**	-4.132231405	**27.7768595**	-19.865546	15.3

**2010**	Park	**13_10b_**	-4.132231405	**15.7768595**	1.1344538	14.9
**2010**	Park	**14_10b_**	3.867768595	**19.7768595**	-71.865546	13.4
**2010**	Park	**14_10b_**	3.867768595	**14.7768595**	5.1344538	16.6
**2010**	Park	**14_10b_**	3.867768595	**-1.2231405**	77.134454	16.1
**2010**	Park	**17_10b_**	-7.132231405	**-40.2231405**	-46.865546	14.8
**2010**	Park	**17_10b_**	-7.132231405	**-2.2231405**	-46.865546	14.7
**2010**	Park	**17_10b_**	-7.132231405	**-12.2231405**		13.9
**2010**	Park	**33_10b_**	14.8677686	**-9.2231405**	17.134454	15.4
**2010**	Park	**33_10b_**	14.8677686	**-4.2231405**	59.134454	15.3
**2010**	Park	**33_10b_**	14.8677686	**-1.2231405**	92.134454	14.5
**2010**	Park	**39_10b_**	1.867768595	**-13.2231405**	-50.865546	13.2
**2010**	Park	**39_10b_**	1.867768595	**-13.2231405**	17.134454	14.4
**2010**	Park	**39_10b_**	1.867768595	**-2.2231405**	49.134454	13
**2010**	Park	**44_10b_**	5.867768595	**-17.2231405**	-22.865546	16
**2010**	Park	**44_10b_**	5.867768595	**-19.2231405**	60.134454	18
**2010**	Park	**44_10b_**	5.867768595	**-29.2231405**	63.134454	17.5
**2010**	Park	**46_10b_**	-1.132231405	**-4.2231405**	-24.865546	18.4
**2010**	Park	**46_10b_**	-1.132231405	**15.7768595**	49.134454	17.2
**2010**	Park	**46_10b_**	-1.132231405	**-15.2231405**	80.134454	15.7
**2010**	Park	**46_10b_**	-1.132231405	**1.7768595**	89.134454	16.8
**2010**	Park	**54_10b_**	2.867768595	**-14.2231405**	-19.865546	17.4
**2010**	Park	**54_10b_**	2.867768595	**-21.2231405**	-17.865546	15.8
**2010**	Park	**54_10b_**	2.867768595	**4.7768595**	0.1344538	15.9
**2010**	Park	**57_10b_**	0.867768595	**20.7768595**	-75.865546	15.7
**2010**	Park	**57_10b_**	0.867768595	**15.7768595**	70.134454	14.5
**2010**	Park	**58_10b_**	0.867768595	**-5.2231405**	-43.865546	17.1
**2010**	Park	**58_10b_**	0.867768595	**7.7768595**	-24.865546	17.6
**2010**	Park	**58_10b_**	0.867768595	**11.7768595**	-0.8655462	17.1
**2010**	Park	**63_10b_**	2.867768595	**22.7768595**	15.134454	11.7
**2010**	Park	**63_10b_**	2.867768595	**-10.2231405**	29.134454	14.1
**2010**	Park	**63_10b_**	2.867768595	**22.7768595**	108.13445	15.6
**2010**	Park	**73_10b_**	14.8677686	**2.7768595**	-75.865546	16.9
**2010**	Park	**73_10b_**	14.8677686	**-27.2231405**	126.13445	15
**2010**	Park	**73_10b_**	14.8677686	**-2.2231405**	126.13445	16.5
**2010**	Park	**76_10b_**	-4.132231405	**1.7768595**	-76.865546	18
**2010**	Park	**76_10b_**	-4.132231405	**20.7768595**	-44.865546	16.6
**2010**	Park	**76_10b_**	-4.132231405	**0.7768595**	32.134454	16
**2010**	Park	**87_10b_**	-4.132231405	**-4.2231405**	-42.865546	16.9
**2010**	Park	**87_10b_**	-4.132231405	**-30.2231405**	-14.865546	15.6
**2010**	Park	**87_10b_**	-4.132231405	**-20.2231405**	5.1344538	16.6
**2010**	Park	**93_10b_**	-3.132231405	**-19.2231405**	-55.865546	17.3
**2010**	Park	**93_10b_**	-3.132231405	**12.7768595**	12.134454	15.5
**2010**	Park	**93_10b_**	-3.132231405	**-3.2231405**	18.134454	16.4
**2010**	Park	**95_10b_**	2.867768595	**-21.2231405**	18.134454	16.4
**2010**	Park	**95_10b_**	2.867768595	**-13.2231405**	25.134454	15.6
**2010**	Park	**95_10b_**	2.867768595	**-17.2231405**	26.134454	15.9
**2010**	Park	**96_10b_**	1.867768595	**16.7768595**	-12.865546	16.3
**2010**	Park	**96_10b_**	1.867768595	**8.7768595**	33.134454	15.3
**2010**	Park	**96_10b_**	1.867768595	**2.7768595**	51.134454	15.9
**2010**	Park	**99_10b_**	-4.132231405	**37.7768595**	-54.865546	14.1
**2010**	Park	**99_10b_**	-4.132231405	**-12.2231405**	-45.865546	13.9
**2010**	Park	**99_10b_**	-4.132231405	**3.7768595**	-4.8655462	12.9
**2010**	Park	**105_10b_**	1.867768595	**-9.2231405**	126.13445	11.7
**2010**	Park	**105_10b_**	1.867768595	**-8.2231405**	126.13445	14.6

**2010**	Park	**105_10b_**	1.867768595	**-4.2231405**	126.13445	15.5
**2010**	Park	**107_10b_**	-5.132231405	**-5.2231405**	54.134454	15.1
**2010**	Park	**107_10b_**	-5.132231405	**-14.2231405**	61.134454	15.2
**2010**	Park	**107_10b_**	-5.132231405	**-6.2231405**	81.134454	15.8
**2010**	Park	**113_10b_**	-6.132231405	**-17.2231405**	-8.8655462	14.5
**2010**	Park	**113_10b_**	-6.132231405	**-29.2231405**	19.134454	14.9
**2010**	Park	**113_10b_**	-6.132231405	**-3.2231405**	25.134454	14
**2010**	Park	**117_10b_**	2.867768595	**18.7768595**	-28.865546	18.3
**2010**	Park	**117_10b_**	2.867768595	**23.7768595**	6.1344538	16.9
**2010**	Park	**117_10b_**	2.867768595	**13.7768595**	32.134454	17.2
**2010**	Park	**120_10b_**	3.867768595	**18.7768595**	-64.865546	14.7
**2010**	Park	**120_10b_**	3.867768595	**-5.2231405**	11.134454	14.7
**2010**	Park	**120_10b_**	3.867768595	**-5.2231405**	32.134454	15.2
**2010**	Park	**125_10b_**	-4.132231405	**10.7768595**	-84.865546	16.6
**2010**	Park	**125_10b_**	-4.132231405	**6.7768595**	-52.865546	17.3
**2010**	Park	**125_10b_**	-4.132231405	**-11.2231405**	-5.8655462	16.8
**2010**	Park	**128_10b_**	-5.132231405	**23.7768595**	-67.865546	15.7
**2010**	Park	**128_10b_**	-5.132231405	**9.7768595**	-42.865546	18.1
**2010**	Park	**128_10b_**	-5.132231405	**1.7768595**	12.134454	14.1
**2010**	Park	**65a_10b_**	-4.132231405	**16.7768595**	45.134454	16.1
**2010**	Park	**65a_10b_**	-4.132231405	**16.7768595**	48.134454	17.7
**2010**	Park	**65a_10b_**	-4.132231405	**4.7768595**	61.134454	16.6
**2010**	Park	**92a_10b_**	-2.132231405	**13.7768595**	-78.865546	19.7
**2010**	Park	**92a_10b_**	-2.132231405	**4.7768595**	-69.865546	17
**2010**	Park	**92a_10b_**	-2.132231405	**17.7768595**	-26.865546	18
**2010**	Park	**8_10z_**	-4.132231405	**1.7768595**	-98.865546	15.3
**2010**	Park	**8_10z_**	-4.132231405	**5.7768595**	-45.865546	16.7
**2010**	Park	**8_10z_**	-4.132231405	**7.7768595**	-40.865546	15.8
**2010**	Park	**9_10z_**	-2.132231405	**21.7768595**	-61.865546	17
**2010**	Park	**9_10z_**	-2.132231405	**16.7768595**	5.1344538	15.7
**2010**	Park	**9_10z_**	-2.132231405	**22.7768595**	17.134454	15.5
**2010**	Park	**11_10z_**	-8.132231405	**-20.2231405**	-13.865546	15.7
**2010**	Park	**11_10z_**	-8.132231405	**-19.2231405**	8.1344538	14.3
**2010**	Park	**11_10z_**	-8.132231405	**3.7768595**	32.134454	16.9
**2010**	Park	**22_10z_**	3.867768595	**-1.2231405**	-19.865546	15.5
**2010**	Park	**22_10z_**	3.867768595	**9.7768595**	-18.865546	15.6
**2010**	Park	**22_10z_**	3.867768595	**-5.2231405**	-2.8655462	15.9
**2010**	Park	**23_10z_**	-0.132231405	**15.7768595**	-76.865546	18.1
**2010**	Park	**23_10z_**	-0.132231405	**37.7768595**	-61.865546	18.4
**2010**	Park	**23_10z_**	-0.132231405	**9.7768595**	-35.865546	17.1
**2010**	Park	**41_10z_**	-0.132231405	**-14.2231405**	-19.865546	15.5
**2010**	Park	**41_10z_**	-0.132231405	**-10.2231405**	-19.865546	14.7
**2010**	Park	**41_10z_**	-0.132231405	**-42.2231405**	52.134454	14.9
**2010**	Park	**44_10z_**	-3.132231405	**11.7768595**	-66.865546	12.2
**2010**	Park	**44_10z_**	-3.132231405	**13.7768595**	-18.865546	13
**2010**	Park	**44_10z_**	-3.132231405	**22.7768595**	-4.8655462	13.8
**2010**	Park	**47_10z_**	-1.132231405	**-5.2231405**	27.134454	13
**2010**	Park	**47_10z_**	-1.132231405	**13.7768595**	42.134454	16.9
**2010**	Park	**47_10z_**	-1.132231405	**-2.2231405**	68.134454	17.6
**2010**	Park	**53_10z_**	18.8677686	**-20.2231405**	-37.865546	16.7
**2010**	Park	**53_10z_**	18.8677686	**-15.2231405**	7.1344538	16.6
**2010**	Park	**58_10z_**	2.867768595	**-3.2231405**	-51.865546	15.8
**2010**	Park	**58_10z_**	2.867768595	**-1.2231405**	-47.865546	16.7
**2010**	Park	**58_10z_**	2.867768595	**-16.2231405**	-6.8655462	16.9

**2010**	Park	**60_10z_**	-3.132231405	**-1.2231405**	-24.865546	14.2
**2010**	Park	**60_10z_**	-3.132231405	**-8.2231405**	-12.865546	14.7
**2010**	Park	**60_10z_**	-3.132231405	**-19.2231405**	61.134454	13.3
**2011**	Forest	**6_11L1_**	5.708860759	**-15.1428571**	-53.333333	15.7
**2011**	Forest	**6_11L1_**	5.708860759	**-15.1428571**	-21.333333	14.5
**2011**	Forest	**6_11L1_**	5.708860759	**-22.1428571**	-12.333333	15.2
**2011**	Forest	**37_11L1_**	1.708860759	**-4.14285714**	-48.333333	16.7
**2011**	Forest	**37_11L1_**	1.708860759	**-9.14285714**	-41.333333	16.8
**2011**	Forest	**37_11L1_**	1.708860759	**2.85714286**	2.6666667	17.8
**2011**	Forest	**62_11L1_**	0.708860759	**16.8571429**	-49.333333	17
**2011**	Forest	**62_11L1_**	0.708860759	**8.85714286**	-33.333333	16.1
**2011**	Forest	**62_11L1_**	0.708860759	**11.8571429**	-25.333333	18.5
**2011**	Forest	**81_11L1_**	0.708860759	**-1.14285714**	-73.333333	9.4
**2011**	Forest	**81_11L1_**	0.708860759	**-39.1428571**	-32.333333	9.1
**2011**	Forest	**81_11L1_**	0.708860759	**-4.14285714**	27.666667	10.7
**2011**	Forest	**93_11L1_**	-1.291139241	**-8.14285714**	-35.333333	16.8
**2011**	Forest	**93_11L1_**	-1.291139241	**0.85714286**	15.666667	17.1
**2011**	Forest	**93_11L1_**	-1.291139241	**1.85714286**	42.666667	17.3
**2011**	Forest	**105_11L1_**	-7.291139241	**9.85714286**	-43.333333	16.3
**2011**	Forest	**105_11L1_**	-7.291139241		-28.333333	18.1
**2011**	Forest	**105_11L1_**	-7.291139241	**1.85714286**	-12.333333	16.8
**2011**	Forest	**105_11L1_**	-7.291139241	**-12.1428571**		17.4
**2011**	Forest	**107_11L1_**	7.708860759	**-11.1428571**	-0.3333333	17
**2011**	Forest	**107_11L1_**	7.708860759	**3.85714286**	22.666667	16.3
**2011**	Forest	**107_11L1_**	7.708860759	**0.85714286**	36.666667	18.3
**2011**	Forest	**119_11L1_**	2.708860759	**16.8571429**	-7.3333333	16.4
**2011**	Forest	**119_11L1_**	2.708860759	**9.85714286**	13.666667	17
**2011**	Forest	**119_11L1_**	2.708860759	**13.8571429**	45.666667	18.5
**2011**	Forest	**160_11L1_**	-2.291139241	**-15.1428571**	-13.333333	16.6
**2011**	Forest	**160_11L1_**	-2.291139241	**1.85714286**	50.666667	17
**2011**	Forest	**160_11L1_**	-2.291139241	**-7.14285714**	86.666667	16.9
**2011**	Forest	**169_11L1_**	2.708860759	**8.85714286**	-77.333333	15.3
**2011**	Forest	**169_11L1_**	2.708860759	**6.85714286**	3.6666667	15.7
**2011**	Forest	**169_11L1_**	2.708860759	**-1.14285714**	6.6666667	16
**2011**	Forest	**182_11L1_**	3.708860759	**-11.1428571**	72.666667	17.3
**2011**	Forest	**182_11L1_**	3.708860759	**-4.14285714**	93.666667	18.1
**2011**	Forest	**182_11L1_**	3.708860759	**-10.1428571**	112.66667	18.7
**2011**	Forest	**184_11L1_**	-4.291139241	**-39.1428571**	28.666667	16
**2011**	Forest	**184_11L1_**	-4.291139241		55.666667	14.9
**2011**	Forest	**184_11L1_**	-4.291139241	**-23.1428571**	77.666667	15.1
**2011**	Forest	**193_11L1_**	-1.291139241	**-2.14285714**	-2.3333333	16.2
**2011**	Forest	**193_11L1_**	-1.291139241	**3.85714286**	13.666667	16.8
**2011**	Forest	**193_11L1_**	-1.291139241	**2.85714286**	38.666667	17.1
**2011**	Forest	**304_11L1_**	0.708860759	**11.8571429**	-49.333333	16.6
**2011**	Forest	**304_11L1_**	0.708860759	**4.85714286**	-27.333333	15.9
**2011**	Forest	**304_11L1_**	0.708860759	**18.8571429**	52.666667	17
**2011**	Forest	**306_11L1_**	3.708860759	**7.85714286**	-34.333333	15.4
**2011**	Forest	**306_11L1_**	3.708860759	**18.8571429**	-8.3333333	16.5
**2011**	Forest	**306_11L1_**	3.708860759	**2.85714286**	22.666667	15.4
**2011**	Forest	**309_11L1_**	-1.291139241	**4.85714286**	-58.333333	17.2
**2011**	Forest	**309_11L1_**	-1.291139241	**-4.14285714**	-33.333333	17.4
**2011**	Forest	**309_11L1_**	-1.291139241	**-9.14285714**	-21.333333	16.7
**2011**	Forest	**326_11L1_**	-3.291139241	**-12.1428571**	-43.333333	17.2
**2011**	Forest	**326_11L1_**	-3.291139241	**-8.14285714**	-9.3333333	16.3

**2011**	Forest	**326_11L1_**	-3.291139241	**-0.14285714**	25.666667	17.3
**2011**	Forest	**328_11L1_**	0.708860759	**14.8571429**	-51.333333	16.1
**2011**	Forest	**328_11L1_**	0.708860759	**4.85714286**	-43.333333	15.7
**2011**	Forest	**328_11L1_**	0.708860759	**-2.14285714**	3.6666667	15.4
**2011**	Forest	**330_11L1_**	3.708860759	**17.8571429**	-51.333333	16.7
**2011**	Forest	**330_11L1_**	3.708860759	**8.85714286**	-47.333333	17.2
**2011**	Forest	**330_11L1_**	3.708860759	**35.8571429**	-36.333333	16.9
**2011**	Forest	**335_11L1_**	0.708860759	**10.8571429**	-31.333333	15.9
**2011**	Forest	**335_11L1_**	0.708860759	**2.85714286**	13.666667	16.5
**2011**	Forest	**335_11L1_**	0.708860759	**5.85714286**	56.666667	16.9
**2011**	Forest	**341_11L1_**	-0.291139241	**-9.14285714**	-5.3333333	18
**2011**	Forest	**341_11L1_**	-0.291139241	**-26.1428571**	6.6666667	16.1
**2011**	Forest	**341_11L1_**	-0.291139241	**-7.14285714**	30.666667	15.2
**2011**	Forest	**350_11L1_**	-1.291139241	**12.8571429**	-27.333333	14.4
**2011**	Forest	**350_11L1_**	-1.291139241	**5.85714286**	-16.333333	15.8
**2011**	Forest	**350_11L1_**	-1.291139241	**14.8571429**	14.666667	16.3
**2011**	Forest	**14_11L2_**	-6.291139241	**-5.14285714**	-66.333333	16.8
**2011**	Forest	**14_11L2_**	-6.291139241	**-16.1428571**	-34.333333	17.1
**2011**	Forest	**14_11L2_**	-6.291139241	**-10.1428571**	68.666667	17.4
**2011**	Forest	**33_11L2_**	0.708860759	**5.85714286**	-16.333333	19.4
**2011**	Forest	**33_11L2_**	0.708860759	**4.85714286**	11.666667	19
**2011**	Forest	**33_11L2_**	0.708860759	**-4.14285714**	50.666667	16.9
**2011**	Forest	**39_11L2_**	-6.291139241	**-36.1428571**	50.666667	14.2
**2011**	Forest	**39_11L2_**	-6.291139241	**-0.14285714**	91.666667	14.3
**2011**	Forest	**39_11L2_**	-6.291139241	**-1.14285714**	120.66667	13.5
**2011**	Forest	**48_11L2_**	1.708860759	**9.85714286**	-73.333333	17.4
**2011**	Forest	**48_11L2_**	1.708860759	**34.8571429**	-38.333333	18.3
**2011**	Forest	**48_11L2_**	1.708860759	**20.8571429**	-35.333333	17.4
**2011**	Park	**10_11b_**	0.451612903	**8.26829268**	-22.121951	12.3
**2011**	Park	**10_11b_**	0.451612903	**-6.73170732**	3.8780488	12.6
**2011**	Park	**10_11b_**	0.451612903	**3.26829268**	101.87805	13.9
**2011**	Park	**20_11b_**	-0.548387097	**11.2682927**	-80.121951	15.9
**2011**	Park	**20_11b_**	-0.548387097	**9.26829268**	-0.1219512	17
**2011**	Park	**20_11b_**	-0.548387097	**24.2682927**	1.8780488	17.2
**2011**	Park	**25_11b_**	-3.548387097	**-0.73170732**	-68.121951	16.7
**2011**	Park	**25_11b_**	-3.548387097	**15.2682927**	-38.121951	15.6
**2011**	Park	**25_11b_**	-3.548387097	**16.2682927**	-21.121951	16.8
**2011**	Park	**28_11b_**	1.451612903	**-6.73170732**	-34.121951	15.4
**2011**	Park	**28_11b_**	1.451612903	**-9.73170732**	45.878049	16.4
**2011**	Park	**32_11b_**	1.451612903	**-21.7317073**	76.878049	15.7
**2011**	Park	**32_11b_**	1.451612903	**-28.7317073**	81.878049	17.2
**2011**	Park	**32_11b_**	1.451612903	**-27.7317073**	93.878049	15.4
**2011**	Park	**35_11b_**	7.451612903	**3.26829268**	-52.121951	16.6
**2011**	Park	**35_11b_**	7.451612903	**-11.7317073**	-30.121951	16.2
**2011**	Park	**35_11b_**	7.451612903	**16.2682927**	-27.121951	18.2
**2011**	Park	**40_11b_**	7.451612903	**-23.7317073**	26.878049	13.5
**2011**	Park	**40_11b_**	7.451612903	**-33.7317073**	68.878049	14.7
**2011**	Park	**40_11b_**	7.451612903	**-19.7317073**	98.878049	14.1
**2011**	Park	**42_11b_**	1.451612903	**-0.73170732**	-37.121951	18.6
**2011**	Park	**42_11b_**	1.451612903	**-6.73170732**	28.878049	18.4
**2011**	Park	**42_11b_**	1.451612903	**3.26829268**	66.878049	19.4
**2011**	Park	**43_11b_**	1.451612903	**6.26829268**	-6.1219512	17
**2011**	Park	**43_11b_**	1.451612903	**11.2682927**	7.8780488	18
**2011**	Park	**43_11b_**	1.451612903	**-5.73170732**	8.8780488	17

**2011**	Park	**44_11b_**	4.451612903	**-3.73170732**	-25.121951	15.6
**2011**	Park	**44_11b_**	4.451612903	**-9.73170732**	-20.121951	15.3
**2011**	Park	**44_11b_**	4.451612903	**-9.73170732**	11.878049	15.8
**2011**	Park	**46_11b_**	-4.548387097	**-2.73170732**	-55.121951	15.7
**2011**	Park	**46_11b_**	-4.548387097	**0.26829268**	-9.1219512	16.6
**2011**	Park	**46_11b_**	-4.548387097	**4.26829268**	68.878049	16.1
**2011**	Park	**48_11b_**	1.451612903	**-1.73170732**	-60.121951	16.2
**2011**	Park	**48_11b_**	1.451612903	**2.26829268**	-26.121951	16.5
**2011**	Park	**48_11b_**	1.451612903	**15.2682927**	-24.121951	16.1
**2011**	Park	**59_11b_**	-2.548387097	**4.26829268**	9.8780488	18
**2011**	Park	**59_11b_**	-2.548387097	**20.2682927**	15.878049	17.5
**2011**	Park	**59_11b_**	-2.548387097	**25.2682927**	41.878049	19
**2011**	Park	**60_11b_**	3.451612903	**11.2682927**	-42.121951	19.7
**2011**	Park	**60_11b_**	3.451612903	**-17.7317073**	112.87805	19.5
**2011**	Park	**60_11b_**	3.451612903	**0.26829268**	113.87805	19.9
**2011**	Park	**64_11b_**	2.451612903	**15.2682927**	-62.121951	18.1
**2011**	Park	**64_11b_**	2.451612903	**11.2682927**	-15.121951	17.5
**2011**	Park	**64_11b_**	2.451612903	**13.2682927**	8.8780488	18
**2011**	Park	**68_11b_**	8.451612903	**3.26829268**	-96.121951	16.4
**2011**	Park	**68_11b_**	8.451612903	**-29.7317073**	-8.1219512	13.4
**2011**	Park	**79_11b_**	2.451612903	**-17.7317073**	64.878049	11.8
**2011**	Park	**79_11b_**	2.451612903	**-0.73170732**	113.87805	16.1
**2011**	Park	**79_11b_**	2.451612903	**-11.7317073**	128.87805	10.7
**2011**	Park	**81_11b_**	-0.548387097	**19.2682927**	-85.121951	16.1
**2011**	Park	**81_11b_**	-0.548387097	**2.26829268**	-28.121951	16.7
**2011**	Park	**81_11b_**	-0.548387097	**4.26829268**	-14.121951	15.9
**2011**	Park	**91_11b_**	-0.548387097	**-6.73170732**	8.8780488	16.2
**2011**	Park	**91_11b_**	-0.548387097	**-12.7317073**	23.878049	18.4
**2011**	Park	**91_11b_**	-0.548387097	**-16.7317073**	49.878049	16.3
**2011**	Park	**92_11b_**	4.451612903	**19.2682927**	-72.121951	16.2
**2011**	Park	**92_11b_**	4.451612903	**29.2682927**	-6.1219512	16.7
**2011**	Park	**92_11b_**	4.451612903	**27.2682927**	1.8780488	17.4
**2011**	Park	**93_11b_**	4.451612903	**-5.73170732**	-24.121951	15.9
**2011**	Park	**93_11b_**	4.451612903	**-22.7317073**	-8.1219512	16.4
**2011**	Park	**93_11b_**	4.451612903	**-10.7317073**	59.878049	14.8
**2011**	Park	**95_11b_**	-0.548387097	**11.2682927**	-59.121951	18.1
**2011**	Park	**95_11b_**	-0.548387097	**9.26829268**	24.878049	18.5
**2011**	Park	**95_11b_**	-0.548387097	**12.2682927**	44.878049	15.9
**2011**	Park	**96_11b_**	2.451612903	**-6.73170732**	-38.121951	13.7
**2011**	Park	**96_11b_**	2.451612903	**-2.73170732**	5.8780488	15.1
**2011**	Park	**96_11b_**	2.451612903	**13.2682927**	6.8780488	15
**2011**	Park	**99_11b_**	-4.548387097	**6.26829268**	-68.121951	12.9
**2011**	Park	**99_11b_**	-4.548387097	**-9.73170732**	-6.1219512	12.1
**2011**	Park	**99_11b_**	-4.548387097	**-11.7317073**	38.878049	12.5
**2011**	Park	**100_11b_**	-4.548387097	**-5.73170732**	-68.121951	15.5
**2011**	Park	**100_11b_**	-4.548387097	**1.26829268**	-0.1219512	15.1
**2011**	Park	**100_11b_**	-4.548387097	**-0.73170732**	80.878049	16.1
**2011**	Park	**106_11b_**	-1.548387097	**10.2682927**	-40.121951	16.5
**2011**	Park	**106_11b_**	-1.548387097	**11.2682927**	79.878049	17.4
**2011**	Park	**106_11b_**	-1.548387097	**1.26829268**	128.87805	16.1
**2011**	Park	**119_11b_**	0.451612903	**28.2682927**	-54.121951	15.2
**2011**	Park	**119_11b_**	0.451612903	**5.26829268**	-53.121951	15.3
**2011**	Park	**119_11b_**	0.451612903	**9.26829268**	-14.121951	15.3
**2011**	Park	**123_11b_**	-5.548387097	**-0.73170732**	4.8780488	14.9

**2011**	Park	**123_11b_**	-5.548387097	**-11.7317073**	6.8780488	14.3
**2011**	Park	**123_11b_**	-5.548387097	**-9.73170732**	83.878049	15.2
**2011**	Park	**125_11b_**	-5.548387097	**-11.7317073**	-49.121951	16
**2011**	Park	**125_11b_**	-5.548387097	**3.26829268**	-27.121951	16.9
**2011**	Park	**125_11b_**	-5.548387097	**-6.73170732**	-12.121951	16
**2011**	Park	**56a_11b_**	0.451612903	**-5.73170732**	-18.121951	16.6
**2011**	Park	**56a_11b_**	0.451612903	**0.26829268**	-6.1219512	17.3
**2011**	Park	**56a_11b_**	0.451612903	**4.26829268**	10.878049	17.3
**2011**	Park	**62a_11b_**	2.451612903	**-1.73170732**	-67.121951	16.1
**2011**	Park	**62a_11b_**	2.451612903	**15.2682927**	-66.121951	15.4
**2011**	Park	**62a_11b_**	2.451612903	**10.2682927**	3.8780488	15.7
**2011**	Park	**65a_11b_**	-1.548387097	**18.2682927**	-87.121951	18.2
**2011**	Park	**65a_11b_**	-1.548387097	**24.2682927**	-52.121951	16.8
**2011**	Park	**65a_11b_**	-1.548387097	**14.2682927**	-37.121951	18.2
**2011**	Park	**92a_11b_**	0.451612903	**13.2682927**	-50.121951	18.2
**2011**	Park	**92a_11b_**	0.451612903	**16.2682927**	-41.121951	18.7
**2011**	Park	**92a_11b_**	0.451612903	**10.2682927**	-21.121951	17.7
**2011**	Park	**5_11z_**	0.451612903	**4.26829268**	-17.121951	15.9
**2011**	Park	**5_11z_**	0.451612903	**4.26829268**	-15.121951	15.6
**2011**	Park	**5_11z_**	0.451612903	**-4.73170732**	128.87805	15.6
**2011**	Park	**6_11z_**	17.4516129	**-31.7317073**	-54.121951	14.4
**2011**	Park	**6_11z_**	17.4516129	**-26.7317073**	43.878049	14.2
**2011**	Park	**6_11z_**	17.4516129	**-12.7317073**	49.878049	14.9
**2011**	Park	**8_11z_**	-5.548387097	**-22.7317073**	0.8780488	11.4
**2011**	Park	**8_11z_**	-5.548387097	**-17.7317073**	38.878049	13
**2011**	Park	**8_11z_**	-5.548387097		98.878049	14.4
**2011**	Park	**8_11z_**	-5.548387097	**-3.73170732**		13.3
**2011**	Park	**14_11z_**	-5.548387097	**-27.7317073**	-48.121951	15
**2011**	Park	**14_11z_**	-5.548387097	**-10.7317073**	-23.121951	13.7
**2011**	Park	**14_11z_**	-5.548387097	**-15.7317073**	87.878049	14.3
**2011**	Park	**34_11z_**	-8.548387097	**12.2682927**	-21.121951	18.3
**2011**	Park	**34_11z_**	-8.548387097	**-6.73170732**	-5.1219512	16.8
**2011**	Park	**34_11z_**	-8.548387097	**3.26829268**	37.878049	17.7
**2011**	Park	**36_11z_**	-6.548387097	**3.26829268**	-61.121951	13.7
**2011**	Park	**36_11z_**	-6.548387097	**-15.7317073**	-54.121951	15.4
**2011**	Park	**41_11z_**	-5.548387097	**0.26829268**	-51.121951	16.1
**2011**	Park	**41_11z_**	-5.548387097	**-8.73170732**	-38.121951	16.5
**2011**	Park	**41_11z_**	-5.548387097	**1.26829268**	-25.121951	16.2
**2011**	Park	**45_11z_**	-3.548387097	**15.2682927**	-8.1219512	17.4
**2011**	Park	**45_11z_**	-3.548387097	**1.26829268**	8.8780488	17.2
**2011**	Park	**45_11z_**	-3.548387097	**2.26829268**	29.878049	18.8
**2011**	Park	**48_11z_**	-2.548387097	**8.26829268**	-77.121951	16
**2011**	Park	**48_11z_**	-2.548387097	**6.26829268**	-42.121951	16.7
**2011**	Park	**48_11z_**	-2.548387097	**8.26829268**	-22.121951	15.7
**2012**	Forest	**5_12L1_**	1.851851852	**20.496063**	-63.810606	17.3
**2012**	Forest	**5_12L1_**	1.851851852	**17.496063**	15.189394	19.5
**2012**	Forest	**5_12L1_**	1.851851852		59.189394	18.5
**2012**	Forest	**5_12L1_**	1.851851852	**23.496063**		17.7
**2012**	Forest	**9_12L1_**	1.851851852	**-0.50393701**	18.189394	18
**2012**	Forest	**9_12L1_**	1.851851852	**12.496063**	46.189394	15.9
**2012**	Forest	**9_12L1_**	1.851851852	**6.49606299**	135.18939	17.9
**2012**	Forest	**9_12L1_**	1.851851852	**6.49606299**		17.4
**2012**	Forest	**12_12L1_**	-1.148148148	**-17.503937**	-37.810606	15.9
**2012**	Forest	**12_12L1_**	-1.148148148	**10.496063**	-30.810606	15.8

**2012**	Forest	**12_12L1_**	-1.148148148	**-3.50393701**	39.189394	18.4
**2012**	Forest	**20_12L1_**	0.851851852	**22.496063**	-102.81061	17.7
**2012**	Forest	**20_12L1_**	0.851851852	**22.496063**	-76.810606	18.2
**2012**	Forest	**20_12L1_**	0.851851852	**23.496063**	-42.810606	17.7
**2012**	Forest	**24_12L1_**	2.851851852	**-0.50393701**	54.189394	17.7
**2012**	Forest	**24_12L1_**	2.851851852	**-0.50393701**	62.189394	16.4
**2012**	Forest	**24_12L1_**	2.851851852	**4.49606299**	145.18939	17.3
**2012**	Forest	**38_12L1_**	1.851851852	**-1.50393701**	9.1893939	19
**2012**	Forest	**38_12L1_**	1.851851852	**9.49606299**	11.189394	16.6
**2012**	Forest	**38_12L1_**	1.851851852	**1.49606299**	66.189394	17
**2012**	Forest	**56_12L1_**	2.851851852	**-4.50393701**	-25.810606	18.4
**2012**	Forest	**56_12L1_**	2.851851852	**5.49606299**	-18.810606	17.6
**2012**	Forest	**56_12L1_**	2.851851852	**8.49606299**	10.189394	16.6
**2012**	Forest	**65_12L1_**	2.851851852	**-3.50393701**	-34.810606	17.8
**2012**	Forest	**65_12L1_**	2.851851852	**2.49606299**	-29.810606	16.8
**2012**	Forest	**65_12L1_**	2.851851852	**-7.50393701**	-15.810606	18.4
**2012**	Forest	**68_12L1_**	1.851851852	**18.496063**	-46.810606	17.2
**2012**	Forest	**68_12L1_**	1.851851852	**26.496063**	60.189394	19.6
**2012**	Forest	**68_12L1_**	1.851851852	**12.496063**	63.189394	17.9
**2012**	Forest	**71_12L1_**	-4.148148148	**-20.503937**	3.1893939	15.5
**2012**	Forest	**71_12L1_**	-4.148148148	**-34.503937**	128.18939	15.2
**2012**	Forest	**71_12L1_**	-4.148148148	**-24.503937**	145.18939	16.5
**2012**	Forest	**82_12L1_**	2.851851852	**11.496063**	36.189394	17.4
**2012**	Forest	**82_12L1_**	2.851851852	**-2.50393701**	73.189394	16.5
**2012**	Forest	**82_12L1_**	2.851851852	**1.49606299**	76.189394	16.3
**2012**	Forest	**83_12L1_**	-2.148148148		-37.810606	17.9
**2012**	Forest	**83_12L1_**	-2.148148148	**10.496063**	-7.8106061	17.5
**2012**	Forest	**83_12L1_**	-2.148148148	**10.496063**	5.1893939	17.2
**2012**	Forest	**83_12L1_**	-2.148148148	**29.496063**		17.7
**2012**	Forest	**102_12L1_**	0.851851852	**-8.50393701**	-19.810606	17.6
**2012**	Forest	**102_12L1_**	0.851851852	**13.496063**	4.1893939	18.2
**2012**	Forest	**102_12L1_**	0.851851852	**5.49606299**	34.189394	18
**2012**	Forest	**103_12L1_**	-1.148148148	**-6.50393701**	-23.810606	18.1
**2012**	Forest	**103_12L1_**	-1.148148148	**-0.50393701**	13.189394	19.1
**2012**	Forest	**103_12L1_**	-1.148148148	**-11.503937**	20.189394	17.3
**2012**	Forest	**107_12L1_**	-4.148148148	**-11.503937**	-58.810606	17.4
**2012**	Forest	**107_12L1_**	-4.148148148	**-15.503937**	11.189394	17
**2012**	Forest	**107_12L1_**	-4.148148148	**-17.503937**	33.189394	18.2
**2012**	Forest	**109_12L1_**	-2.148148148	**-17.503937**	-45.810606	16.3
**2012**	Forest	**109_12L1_**	-2.148148148	**-8.50393701**	-10.810606	17.2
**2012**	Forest	**109_12L1_**	-2.148148148	**-20.503937**	17.189394	17.4
**2012**	Forest	**117_12L1_**	0.851851852	**12.496063**	0.1893939	15.9
**2012**	Forest	**117_12L1_**	0.851851852	**-6.50393701**	47.189394	16
**2012**	Forest	**117_12L1_**	0.851851852	**-15.503937**	78.189394	16.5
**2012**	Forest	**128_12L1_**	-4.148148148	**3.49606299**	-35.810606	19.5
**2012**	Forest	**128_12L1_**	-4.148148148	**2.49606299**	9.1893939	19.3
**2012**	Forest	**128_12L1_**	-4.148148148	**-7.50393701**	66.189394	18.3
**2012**	Forest	**156_12L1_**	-0.148148148	**0.49606299**	-18.810606	17.5
**2012**	Forest	**156_12L1_**	-0.148148148	**7.49606299**	-17.810606	17.3
**2012**	Forest	**156_12L1_**	-0.148148148	**3.49606299**	18.189394	16.3
**2012**	Forest	**161_12L1_**	-5.148148148	**6.49606299**	-76.810606	19.2
**2012**	Forest	**161_12L1_**	-5.148148148	**18.496063**	-27.810606	17.4
**2012**	Forest	**161_12L1_**	-5.148148148	**1.49606299**	13.189394	18.5
**2012**	Forest	**163_12L1_**	-0.148148148		-67.810606	17.4

**2012**	Forest	**163_12L1_**	-0.148148148		-67.810606	18.9
**2012**	Forest	**163_12L1_**	-0.148148148		2.1893939	18.8
**2012**	Forest	**166_12L1_**	-3.148148148		-69.810606	16.9
**2012**	Forest	**166_12L1_**	-3.148148148		-25.810606	17.8
**2012**	Forest	**166_12L1_**	-3.148148148		-3.8106061	18.4
**2012**	Forest	**174_12L1_**	-0.148148148	**7.49606299**	-16.810606	18.2
**2012**	Forest	**174_12L1_**	-0.148148148	**-11.503937**	1.1893939	17.3
**2012**	Forest	**174_12L1_**	-0.148148148	**6.49606299**	6.1893939	16.9
**2012**	Forest	**182_12L1_**	3.851851852	**-36.503937**	-37.810606	18.9
**2012**	Forest	**182_12L1_**	3.851851852	**-14.503937**	24.189394	16.7
**2012**	Forest	**182_12L1_**	3.851851852	**-32.503937**	42.189394	17.7
**2012**	Forest	**184_12L1_**	-3.148148148	**21.496063**	-19.810606	16.8
**2012**	Forest	**184_12L1_**	-3.148148148	**12.496063**	43.189394	19.3
**2012**	Forest	**184_12L1_**	-3.148148148	**23.496063**	59.189394	17.9
**2012**	Forest	**304_12L1_**	3.851851852	**-7.50393701**	-29.810606	15.5
**2012**	Forest	**304_12L1_**	3.851851852	**-12.503937**	-28.810606	15.7
**2012**	Forest	**304_12L1_**	3.851851852	**-23.503937**	-11.810606	15.2
**2012**	Forest	**314_12L1_**	-5.148148148	**0.49606299**	-63.810606	17.4
**2012**	Forest	**314_12L1_**	-5.148148148	**-2.50393701**	24.189394	19.8
**2012**	Forest	**314_12L1_**	-5.148148148	**2.49606299**	61.189394	19.4
**2012**	Forest	**319_12L1_**	2.851851852	**14.496063**	-34.810606	16.8
**2012**	Forest	**319_12L1_**	2.851851852	**19.496063**	1.1893939	17.2
**2012**	Forest	**319_12L1_**	2.851851852	**15.496063**	19.189394	17.7
**2012**	Forest	**321_12L1_**	2.851851852	**-4.50393701**	-40.810606	17.7
**2012**	Forest	**321_12L1_**	2.851851852	**3.49606299**	17.189394	17.4
**2012**	Forest	**321_12L1_**	2.851851852	**-1.50393701**	26.189394	16.8
**2012**	Forest	**323_12L1_**	4.851851852	**-18.503937**	-8.8106061	17
**2012**	Forest	**323_12L1_**	4.851851852	**-18.503937**	34.189394	15.6
**2012**	Forest	**323_12L1_**	4.851851852	**-14.503937**	53.189394	16.6
**2012**	Forest	**340_12L1_**	-0.148148148	**13.496063**	-11.810606	15.9
**2012**	Forest	**340_12L1_**	-0.148148148	**5.49606299**	5.1893939	17.2
**2012**	Forest	**340_12L1_**	-0.148148148	**1.49606299**	13.189394	16
**2012**	Forest	**341_12L1_**	-0.148148148	**-8.50393701**	-20.810606	18.8
**2012**	Forest	**341_12L1_**	-0.148148148	**0.49606299**	23.189394	17.5
**2012**	Forest	**341_12L1_**	-0.148148148	**-4.50393701**	43.189394	17.8
**2012**	Forest	**1_12L2_**	-4.148148148	**7.49606299**	-38.810606	17.8
**2012**	Forest	**1_12L2_**	-4.148148148	**3.49606299**	-28.810606	18.9
**2012**	Forest	**1_12L2_**	-4.148148148	**-2.50393701**	-18.810606	18
**2012**	Forest	**3_12L2_**	0.851851852	**-9.50393701**	-6.8106061	17.8
**2012**	Forest	**3_12L2_**	0.851851852	**-19.503937**	-0.8106061	17.4
**2012**	Forest	**3_12L2_**	0.851851852	**-23.503937**	9.1893939	15.7
**2012**	Forest	**7_12L2_**	-0.148148148	**17.496063**	-78.810606	18.6
**2012**	Forest	**7_12L2_**	-0.148148148	**9.49606299**	-66.810606	17.9
**2012**	Forest	**7_12L2_**	-0.148148148	**0.49606299**	-53.810606	17.5
**2012**	Forest	**10_12L2_**	1.851851852	**-7.50393701**	-86.810606	15.4
**2012**	Forest	**10_12L2_**	1.851851852	**-15.503937**	-23.810606	15.3
**2012**	Forest	**10_12L2_**	1.851851852	**-12.503937**	-11.810606	15.4
**2012**	Forest	**13_12L2_**	-5.148148148	**2.49606299**	-32.810606	18.9
**2012**	Forest	**13_12L2_**	-5.148148148	**-1.50393701**	-30.810606	18.6
**2012**	Forest	**13_12L2_**	-5.148148148	**-7.50393701**	47.189394	18.4
**2012**	Forest	**16_12L2_**	-5.148148148	**21.496063**	-96.810606	15.9
**2012**	Forest	**16_12L2_**	-5.148148148	**21.496063**	-75.810606	16
**2012**	Forest	**16_12L2_**	-5.148148148	**30.496063**	-64.810606	17.4
**2012**	Forest	**25_12L2_**	2.851851852	**-12.503937**	16.189394	16.3

**2012**	Forest	**25_12L2_**	2.851851852	**10.496063**	83.189394	16
**2012**	Forest	**25_12L2_**	2.851851852	**-0.50393701**	85.189394	14.9
**2012**	Forest	**27_12L2_**	3.851851852	**-11.503937**	-29.810606	15.8
**2012**	Forest	**27_12L2_**	3.851851852	**-4.50393701**	9.1893939	17.1
**2012**	Forest	**27_12L2_**	3.851851852	**-3.50393701**	28.189394	15.7
**2012**	Forest	**30_12L2_**	2.851851852	**-5.50393701**	-93.810606	15.3
**2012**	Forest	**30_12L2_**	2.851851852	**-17.503937**	-57.810606	15.2
**2012**	Forest	**30_12L2_**	2.851851852	**-20.503937**	-36.810606	16
**2012**	Forest	**31_12L2_**	-0.148148148	**19.496063**	-66.810606	14.9
**2012**	Forest	**31_12L2_**	-0.148148148	**11.496063**	-55.810606	16.5
**2012**	Forest	**31_12L2_**	-0.148148148	**5.49606299**	-30.810606	15
**2012**	Forest	**36_12L2_**	-1.148148148	**-4.50393701**	-1.8106061	17.3
**2012**	Forest	**36_12L2_**	-1.148148148	**-1.50393701**	5.1893939	17.5
**2012**	Forest	**36_12L2_**	-1.148148148	**-0.50393701**	44.189394	18.2
**2012**	Forest	**41_12L2_**	-0.148148148	**-16.503937**	30.189394	17.8
**2012**	Forest	**41_12L2_**	-0.148148148	**-11.503937**	36.189394	19
**2012**	Forest	**41_12L2_**	-0.148148148	**-13.503937**	38.189394	19.6
**2012**	Park	**10_12b_**	-0.62962963	**18.2234637**	-94.643678	13
**2012**	Park	**10_12b_**	-0.62962963	**15.2234637**	-87.643678	15
**2012**	Park	**10_12b_**	-0.62962963	**9.22346369**	-43.643678	13.8
**2012**	Park	**14_12b_**	-2.62962963	**8.22346369**	-93.643678	15.6
**2012**	Park	**14_12b_**	-2.62962963	**-4.77653631**	-71.643678	16.9
**2012**	Park	**14_12b_**	-2.62962963	**4.22346369**	-26.643678	16.3
**2012**	Park	**14_12b_**	-2.62962963	**-12.7765363**		15.7
**2012**	Park	**19_12b_**	-1.62962963	**-20.7765363**	-49.643678	14.9
**2012**	Park	**19_12b_**	-1.62962963	**-3.77653631**	0.3563218	16.4
**2012**	Park	**19_12b_**	-1.62962963	**23.2234637**	44.356322	14.3
**2012**	Park	**26_12b_**	-2.62962963	**9.22346369**	-67.643678	16.8
**2012**	Park	**26_12b_**	-2.62962963	**17.2234637**	-54.643678	17.1
**2012**	Park	**26_12b_**	-2.62962963	**8.22346369**	-41.643678	16.2
**2012**	Park	**28_12b_**	-0.62962963	**12.2234637**	-57.643678	15.6
**2012**	Park	**28_12b_**	-0.62962963	**9.22346369**	-5.6436782	17.5
**2012**	Park	**28_12b_**	-0.62962963	**13.2234637**	21.356322	17.2
**2012**	Park	**30_12b_**	7.37037037	**6.22346369**	-54.643678	16.4
**2012**	Park	**30_12b_**	7.37037037	**-0.77653631**	-37.643678	14.1
**2012**	Park	**30_12b_**	7.37037037	**5.22346369**	52.356322	13.9
**2012**	Park	**31_12b_**	3.37037037	**22.2234637**	-90.643678	17.8
**2012**	Park	**31_12b_**	3.37037037	**32.2234637**	-86.643678	17
**2012**	Park	**31_12b_**	3.37037037	**17.2234637**	-61.643678	17.9
**2012**	Park	**32_12b_**	6.37037037	**-7.77653631**	13.356322	17.2
**2012**	Park	**32_12b_**	6.37037037	**-14.7765363**	74.356322	16.3
**2012**	Park	**32_12b_**	6.37037037	**-20.7765363**	85.356322	17.6
**2012**	Park	**33_12b_**	7.37037037	**-14.7765363**	-72.643678	14.1
**2012**	Park	**33_12b_**	7.37037037	**-4.77653631**	-36.643678	14.4
**2012**	Park	**33_12b_**	7.37037037	**-10.7765363**	0.3563218	14.7
**2012**	Park	**33_12b_**	7.37037037	**12.2234637**		13.4
**2012**	Park	**34_12b_**	2.37037037	**5.22346369**	-2.6436782	13.6
**2012**	Park	**34_12b_**	2.37037037	**7.22346369**	121.35632	14.6
**2012**	Park	**34_12b_**	2.37037037	**28.2234637**	121.35632	10
**2012**	Park	**35_12b_**	5.37037037	**8.22346369**	-36.643678	18.5
**2012**	Park	**35_12b_**	5.37037037	**5.22346369**	-3.6436782	17.3
**2012**	Park	**35_12b_**	5.37037037	**-16.7765363**	37.356322	16.2
**2012**	Park	**37_12b_**	9.37037037		-6.6436782	15.7
**2012**	Park	**37_12b_**	9.37037037		-3.6436782	14.1

**2012**	Park	**37_12b_**	9.37037037		37.356322	13.5
**2012**	Park	**40_12b_**	3.37037037	**5.22346369**	-101.64368	15.7
**2012**	Park	**40_12b_**	3.37037037	**4.22346369**	-88.643678	15.1
**2012**	Park	**40_12b_**	3.37037037	**8.22346369**	-77.643678	16.3
**2012**	Park	**43_12b_**	0.37037037	**-7.77653631**	-33.643678	17.7
**2012**	Park	**43_12b_**	0.37037037	**-0.77653631**	-19.643678	17.9
**2012**	Park	**43_12b_**	0.37037037	**-0.77653631**	8.3563218	17.3
**2012**	Park	**45_12b_**	0.37037037	**9.22346369**	-76.643678	17.7
**2012**	Park	**45_12b_**	0.37037037	**12.2234637**	-42.643678	17.8
**2012**	Park	**45_12b_**	0.37037037	**20.2234637**	-17.643678	17.4
**2012**	Park	**47_12b_**	0.37037037	**-21.7765363**	-19.643678	16.8
**2012**	Park	**47_12b_**	0.37037037	**-15.7765363**	22.356322	16.8
**2012**	Park	**47_12b_**	0.37037037	**-24.7765363**	40.356322	16.3
**2012**	Park	**48_12b_**	-0.62962963	**21.2234637**	-4.6436782	18.3
**2012**	Park	**48_12b_**	-0.62962963	**2.22346369**	0.3563218	18.2
**2012**	Park	**48_12b_**	-0.62962963	**1.22346369**	37.356322	18.4
**2012**	Park	**50_12b_**	3.37037037	**-27.7765363**	-22.643678	15.7
**2012**	Park	**50_12b_**	3.37037037	**-23.7765363**	-11.643678	16.1
**2012**	Park	**50_12b_**	3.37037037	**-20.7765363**	15.356322	17.2
**2012**	Park	**54_12b_**	6.37037037	**5.22346369**	-13.643678	16.5
**2012**	Park	**54_12b_**	6.37037037	**13.2234637**	-10.643678	15.3
**2012**	Park	**54_12b_**	6.37037037	**-10.7765363**	13.356322	14.6
**2012**	Park	**57_12b_**	-0.62962963	**8.22346369**	-14.643678	17.8
**2012**	Park	**57_12b_**	-0.62962963	**2.22346369**	37.356322	17.2
**2012**	Park	**57_12b_**	-0.62962963	**1.22346369**	50.356322	17.4
**2012**	Park	**60_12b_**	4.37037037	**26.2234637**	-98.643678	15.9
**2012**	Park	**60_12b_**	4.37037037	**6.22346369**	-20.643678	16
**2012**	Park	**60_12b_**	4.37037037	**5.22346369**	74.356322	15.4
**2012**	Park	**64_12b_**	1.37037037	**20.2234637**	37.356322	16.6
**2012**	Park	**64_12b_**	1.37037037	**19.2234637**	42.356322	17.2
**2012**	Park	**64_12b_**	1.37037037	**11.2234637**	44.356322	17.1
**2012**	Park	**67_12b_**	5.37037037	**-19.7765363**	-4.6436782	15.8
**2012**	Park	**67_12b_**	5.37037037	**-36.7765363**	45.356322	15.7
**2012**	Park	**67_12b_**	5.37037037	**-29.7765363**	71.356322	14.2
**2012**	Park	**68_12b_**	-1.62962963	**-5.77653631**	-118.64368	16.5
**2012**	Park	**68_12b_**	-1.62962963	**1.22346369**	-56.643678	16.8
**2012**	Park	**68_12b_**	-1.62962963	**-1.77653631**	38.356322	16.3
**2012**	Park	**70_12b_**	3.37037037	**-4.77653631**	12.356322	16.2
**2012**	Park	**70_12b_**	3.37037037	**12.2234637**	53.356322	16.6
**2012**	Park	**70_12b_**	3.37037037	**18.2234637**	53.356322	17
**2012**	Park	**75_12b_**	0.37037037	**14.2234637**	-29.643678	15.7
**2012**	Park	**75_12b_**	0.37037037	**9.22346369**	52.356322	15.4
**2012**	Park	**75_12b_**	0.37037037	**9.22346369**	95.356322	15.6
**2012**	Park	**81_12b_**	-2.62962963	**15.2234637**	-64.643678	16.2
**2012**	Park	**81_12b_**	-2.62962963	**21.2234637**	-53.643678	16.2
**2012**	Park	**81_12b_**	-2.62962963	**-0.77653631**	-6.6436782	17.2
**2012**	Park	**84_12b_**	-0.62962963	**3.22346369**	-39.643678	15.3
**2012**	Park	**84_12b_**	-0.62962963	**-1.77653631**	-7.6436782	15.7
**2012**	Park	**84_12b_**	-0.62962963	**-1.77653631**	14.356322	16.7
**2012**	Park	**85_12b_**	4.37037037	**5.22346369**	-62.643678	16.7
**2012**	Park	**85_12b_**	4.37037037	**0.22346369**	28.356322	16.5
**2012**	Park	**85_12b_**	4.37037037	**5.22346369**	58.356322	16.7
**2012**	Park	**87_12b_**	0.37037037	**27.2234637**	-3.6436782	16
**2012**	Park	**87_12b_**	0.37037037	**16.2234637**	39.356322	14.1

**2012**	Park	**87_12b_**	0.37037037	**10.2234637**	63.356322	11
**2012**	Park	**91_12b_**	-1.62962963	**-17.7765363**	61.356322	18.8
**2012**	Park	**91_12b_**	-1.62962963	**1.22346369**	63.356322	17.2
**2012**	Park	**91_12b_**	-1.62962963	**-13.7765363**	121.35632	18
**2012**	Park	**92_12b_**	3.37037037	**14.2234637**	-34.643678	18.5
**2012**	Park	**92_12b_**	3.37037037	**27.2234637**	-16.643678	18.4
**2012**	Park	**92_12b_**	3.37037037	**26.2234637**	-0.6436782	17.7
**2012**	Park	**93_12b_**	-0.62962963	**5.22346369**	-56.643678	17.5
**2012**	Park	**93_12b_**	-0.62962963	**-12.7765363**	-46.643678	17.6
**2012**	Park	**93_12b_**	-0.62962963	**-9.77653631**	-3.6436782	18.3
**2012**	Park	**95_12b_**	1.37037037	**-18.7765363**	83.356322	15.8
**2012**	Park	**95_12b_**	1.37037037	**-27.7765363**	91.356322	15.1
**2012**	Park	**95_12b_**	1.37037037	**-9.77653631**		17.1
**2012**	Park	**97_12b_**	-0.62962963	**21.2234637**	7.3563218	18.4
**2012**	Park	**97_12b_**	-0.62962963	**24.2234637**	12.356322	18.4
**2012**	Park	**97_12b_**	-0.62962963	**15.2234637**	97.356322	18.6
**2012**	Park	**100_12b_**	-1.62962963	**20.2234637**	-95.643678	16.4
**2012**	Park	**100_12b_**	-1.62962963	**14.2234637**	-56.643678	17.6
**2012**	Park	**100_12b_**	-1.62962963	**22.2234637**	59.356322	17.3
**2012**	Park	**108_12b_**	2.37037037	**-8.77653631**	-8.6436782	13.4
**2012**	Park	**108_12b_**	2.37037037	**-23.7765363**	28.356322	14.4
**2012**	Park	**108_12b_**	2.37037037	**-22.7765363**	58.356322	15.1
**2012**	Park	**110_12b_**	-10.62962963	**10.2234637**	-31.643678	16.7
**2012**	Park	**110_12b_**	-10.62962963	**14.2234637**	0.3563218	16.1
**2012**	Park	**110_12b_**	-10.62962963	**-0.77653631**	32.356322	16.6
**2012**	Park	**113_12b_**	1.37037037	**-5.77653631**	-6.6436782	16.5
**2012**	Park	**113_12b_**	1.37037037	**-4.77653631**	40.356322	15.8
**2012**	Park	**113_12b_**	1.37037037	**-14.7765363**	86.356322	16.9
**2012**	Park	**116_12b_**	-2.62962963	**11.2234637**	-31.643678	17.8
**2012**	Park	**116_12b_**	-2.62962963	**15.2234637**	-16.643678	16.2
**2012**	Park	**116_12b_**	-2.62962963	**8.22346369**	5.3563218	16.5
**2012**	Park	**117_12b_**	-10.62962963	**-3.77653631**		16.2
**2012**	Park	**117_12b_**	-10.62962963	**10.2234637**		16.2
**2012**	Park	**117_12b_**	-10.62962963	**10.2234637**		17.1
**2012**	Park	**121_12b_**	0.37037037		-3.6436782	11
**2012**	Park	**121_12b_**	0.37037037	**-43.7765363**	121.35632	11.5
**2012**	Park	**121_12b_**	0.37037037	**-37.7765363**	121.35632	11.1
**2012**	Park	**121_12b_**	0.37037037	**-51.7765363**		10.4
**2012**	Park	**121_12b_**	0.37037037	**-40.7765363**		11
**2012**	Park	**124_12b_**	-0.62962963		-28.643678	12.6
**2012**	Park	**124_12b_**	-0.62962963		108.35632	14.1
**2012**	Park	**124_12b_**	-0.62962963	**-12.7765363**	121.35632	14.5
**2012**	Park	**124_12b_**	-0.62962963	**7.22346369**		13.7
**2012**	Park	**124_12b_**	-0.62962963	**11.2234637**		13.9
**2012**	Park	**125_12b_**	-8.62962963	**-8.77653631**	-90.643678	14.8
**2012**	Park	**125_12b_**	-8.62962963	**-4.77653631**	-79.643678	15.3
**2012**	Park	**125_12b_**	-8.62962963	**-5.77653631**	-63.643678	15.3
**2012**	Park	**56a_12b_**	-1.62962963	**10.2234637**	-39.643678	18
**2012**	Park	**56a_12b_**	-1.62962963	**20.2234637**	-8.6436782	16.7
**2012**	Park	**56a_12b_**	-1.62962963	**4.22346369**	69.356322	16.7
**2012**	Park	**65a_12b_**	3.37037037		-58.643678	16
**2012**	Park	**65a_12b_**	3.37037037		-48.643678	14.7
**2012**	Park	**65a_12b_**	3.37037037		17.356322	15.8
**2012**	Park	**92a_12b_**	-0.62962963	**25.2234637**	-7.6436782	18.1

**2012**	Park	**92a_12b_**	-0.62962963	**1.22346369**	28.356322	16
**2012**	Park	**92a_12b_**	-0.62962963	**14.2234637**	70.356322	16.9
**2012**	Park	**3_12z_**	-6.62962963	**-12.7765363**	11.356322	16
**2012**	Park	**3_12z_**	-6.62962963	**-14.7765363**	46.356322	15.8
**2012**	Park	**3_12z_**	-6.62962963	**-19.7765363**	48.356322	14.5
**2012**	Park	**5_12z_**	2.37037037	**-58.7765363**	-30.643678	9.9
**2012**	Park	**5_12z_**	2.37037037		-22.643678	11.4
**2012**	Park	**5_12z_**	2.37037037	**-61.7765363**	35.356322	10.6
**2012**	Park	**5_12z_**	2.37037037	**-29.7765363**		10.8
**2012**	Park	**10_12z_**	-7.62962963	**0.22346369**	-21.643678	14.2
**2012**	Park	**10_12z_**	-7.62962963	**-7.77653631**	-15.643678	15.1
**2012**	Park	**10_12z_**	-7.62962963	**-14.7765363**	75.356322	15.3
**2012**	Park	**16_12z_**	-3.62962963	**1.22346369**	-70.643678	17
**2012**	Park	**16_12z_**	-3.62962963	**-17.7765363**	-11.643678	16
**2012**	Park	**16_12z_**	-3.62962963	**-29.7765363**	121.35632	15.1
**2012**	Park	**20_12z_**	1.37037037	**15.2234637**	-110.64368	14
**2012**	Park	**20_12z_**	1.37037037	**13.2234637**		11.2
**2012**	Park	**20_12z_**	1.37037037	**31.2234637**		12.6
**2012**	Park	**23_12z_**	-1.62962963	**-12.7765363**	-56.643678	15.8
**2012**	Park	**23_12z_**	-1.62962963	**-14.7765363**	23.356322	17.3
**2012**	Park	**23_12z_**	-1.62962963	**-11.7765363**	45.356322	18.5
**2012**	Park	**27_12z_**	-5.62962963	**12.2234637**	-52.643678	17
**2012**	Park	**27_12z_**	-5.62962963	**-4.77653631**	-42.643678	18.4
**2012**	Park	**27_12z_**	-5.62962963	**-9.77653631**	76.356322	17.2
**2012**	Park	**27_12z_**	-5.62962963	**6.22346369**		16.9
**2012**	Park	**35_12z_**	-8.62962963	**-8.77653631**	6.3563218	16.7
**2012**	Park	**35_12z_**	-8.62962963	**-20.7765363**	11.356322	16.3
**2012**	Park	**35_12z_**	-8.62962963	**-9.77653631**	38.356322	16.1
**2012**	Park	**37_12z_**	1.37037037	**3.22346369**	-73.643678	16.5
**2012**	Park	**37_12z_**	1.37037037	**-3.77653631**	0.3563218	17.1
**2012**	Park	**37_12z_**	1.37037037	**-3.77653631**	12.356322	16.9
**2012**	Park	**37_12z_**	1.37037037	**-0.77653631**		16.5
**2012**	Park	**44_12z_**	-2.62962963	**10.2234637**	-31.643678	16.4
**2012**	Park	**44_12z_**	-2.62962963	**5.22346369**	-18.643678	18.8
**2012**	Park	**44_12z_**	-2.62962963	**9.22346369**	57.356322	17.7
**2012**	Park	**54_12z_**	3.37037037	**-15.7765363**	-26.643678	16.1
**2012**	Park	**54_12z_**	3.37037037	**16.2234637**	-16.643678	16
**2012**	Park	**54_12z_**	3.37037037	**-3.77653631**	86.356322	15.9
**2012**	Park	**57_12z_**	4.37037037	**23.2234637**	-77.643678	15.9
**2012**	Park	**57_12z_**	4.37037037	**-11.7765363**	-0.6436782	15.4
**2012**	Park	**57_12z_**	4.37037037	**9.22346369**	0.3563218	15.5
**2012**	Park	**60_12z_**	-10.62962963	**4.22346369**	-37.643678	14.8
**2012**	Park	**60_12z_**	-10.62962963	**-3.77653631**	0.3563218	15.7
**2012**	Park	**60_12z_**	-10.62962963	**4.22346369**	4.3563218	15.5
**2013**	Forest	**2_13L1_**	0.689320388	**-8.53398058**	-34.29703	18.9
**2013**	Forest	**2_13L1_**	0.689320388	**1.46601942**	-28.29703	17.3
**2013**	Forest	**2_13L1_**	0.689320388	**7.46601942**	23.70297	17.2
**2013**	Forest	**27_13_L1_**	-0.310679612	**0.46601942**	-21.29703	17.3
**2013**	Forest	**27_13_L1_**	-0.310679612	**-12.5339806**	-13.29703	16.7
**2013**	Forest	**27_13_L1_**	-0.310679612	**6.46601942**	4.7029703	17
**2013**	Forest	**30_13L1_**	-1.310679612	**-6.53398058**	-19.29703	18.1
**2013**	Forest	**30_13L1_**	-1.310679612	**-4.53398058**	15.70297	17.4
**2013**	Forest	**30_13L1_**	-1.310679612	**1.46601942**	49.70297	18
**2013**	Forest	**47_13L1_**	2.689320388	**18.4660194**	-59.29703	18.2

**2013**	Forest	**47_13L1_**	2.689320388	**10.4660194**	-11.29703	17.6
**2013**	Forest	**47_13L1_**	2.689320388	**16.4660194**	8.7029703	19
**2013**	Forest	**63_13L1_**	-0.310679612	**-12.5339806**	28.70297	17.1
**2013**	Forest	**63_13L1_**	-0.310679612	**-10.5339806**	34.70297	16.9
**2013**	Forest	**63_13L1_**	-0.310679612	**-8.53398058**	105.70297	15.8
**2013**	Forest	**80_13L1_**	0.689320388	**30.4660194**	-60.29703	13.9
**2013**	Forest	**80_13L1_**	0.689320388	**6.46601942**	13.70297	15.5
**2013**	Forest	**80_13L1_**	0.689320388	**17.4660194**	21.70297	15.1
**2013**	Forest	**81_13L1_**	2.689320388	**3.46601942**	-49.29703	17.6
**2013**	Forest	**81_13L1_**	2.689320388	**-19.5339806**	15.70297	17.9
**2013**	Forest	**81_13L1_**	2.689320388	**-5.53398058**	15.70297	17.4
**2013**	Forest	**102_13L1_**	-2.310679612	**-8.53398058**	-1.2970297	17.5
**2013**	Forest	**102_13L1_**	-2.310679612	**-12.5339806**	10.70297	17.7
**2013**	Forest	**102_13L1_**	-2.310679612	**0.46601942**	87.70297	17.2
**2013**	Forest	**104_13L1_**	2.689320388	**-10.5339806**	43.70297	15.1
**2013**	Forest	**104_13L1_**	2.689320388	**-2.53398058**	54.70297	17.1
**2013**	Forest	**104_13L1_**	2.689320388	**-10.5339806**	83.70297	16.3
**2013**	Forest	**105_13L1_**	-1.310679612	**25.4660194**	7.7029703	18.7
**2013**	Forest	**105_13L1_**	-1.310679612	**5.46601942**	66.70297	18.5
**2013**	Forest	**105_13L1_**	-1.310679612	**12.4660194**	87.70297	18.5
**2013**	Forest	**129_13L1_**	6.689320388	**-1.53398058**	-29.29703	14.7
**2013**	Forest	**129_13L1_**	6.689320388	**-15.5339806**	-12.29703	15.6
**2013**	Forest	**129_13L1_**	6.689320388	**8.46601942**	32.70297	16
**2013**	Forest	**150_13L1_**	-0.310679612	**15.4660194**	-43.29703	14.7
**2013**	Forest	**150_13L1_**	-0.310679612	**23.4660194**	-37.29703	15.4
**2013**	Forest	**150_13L1_**	-0.310679612	**12.4660194**	-27.29703	14.6
**2013**	Forest	**161_13L1_**	2.689320388	**16.4660194**	-66.29703	16.8
**2013**	Forest	**161_13L1_**	2.689320388	**13.4660194**	-2.2970297	16.6
**2013**	Forest	**161_13L1_**	2.689320388	**23.4660194**	68.70297	17.2
**2013**	Forest	**163_13L1_**	-1.310679612	**-10.5339806**	-20.29703	18.6
**2013**	Forest	**163_13L1_**	-1.310679612	**-6.53398058**	12.70297	17.9
**2013**	Forest	**163_13L1_**	-1.310679612	**-11.5339806**	38.70297	16.6
**2013**	Forest	**164_13L1_**	-4.310679612	**10.4660194**	-86.29703	16.5
**2013**	Forest	**164_13L1_**	-4.310679612	**14.4660194**	-47.29703	18.1
**2013**	Forest	**164_13L1_**	-4.310679612	**3.46601942**	-7.2970297	17.6
**2013**	Forest	**165_13L1_**	1.689320388	**-3.53398058**	-58.29703	17.3
**2013**	Forest	**165_13L1_**	1.689320388	**-8.53398058**	-49.29703	17.6
**2013**	Forest	**165_13L1_**	1.689320388	**2.46601942**	-25.29703	16.4
**2013**	Forest	**166_13L1_**	0.689320388	**-11.5339806**	-53.29703	16.8
**2013**	Forest	**166_13L1_**	0.689320388	**4.46601942**	-50.29703	16.5
**2013**	Forest	**166_13L1_**	0.689320388	**-0.53398058**	-36.29703	15.8
**2013**	Forest	**175_13L1_**	-1.310679612	**-11.5339806**	-33.29703	17.7
**2013**	Forest	**175_13L1_**	-1.310679612	**-9.53398058**	-15.29703	16.4
**2013**	Forest	**175_13L1_**	-1.310679612	**-0.53398058**	4.7029703	18.4
**2013**	Forest	**182_13L1_**	1.689320388	**13.4660194**	-41.29703	12.7
**2013**	Forest	**182_13L1_**	1.689320388	**13.4660194**	28.70297	14.7
**2013**	Forest	**182_13L1_**	1.689320388	**-3.53398058**	30.70297	15.1
**2013**	Forest	**197_13L1_**	-1.310679612	**3.46601942**	-63.29703	16.2
**2013**	Forest	**197_13L1_**	-1.310679612	**21.4660194**	-9.2970297	17.7
**2013**	Forest	**197_13L1_**	-1.310679612	**-13.5339806**	145.70297	17.3
**2013**	Forest	**304_13L1_**	-0.310679612	**1.46601942**	-36.29703	15.4
**2013**	Forest	**304_13L1_**	-0.310679612	**8.46601942**	-9.2970297	17.1
**2013**	Forest	**304_13L1_**	-0.310679612	**-5.53398058**	-5.2970297	16.5
**2013**	Forest	**307_13L1_**	1.689320388	**-25.5339806**	3.7029703	12.8

**2013**	Forest	**307_13L1_**	1.689320388	**-30.5339806**	4.7029703	14.9
**2013**	Forest	**307_13L1_**	1.689320388	**-38.5339806**	25.70297	13.5
**2013**	Forest	**324_13L1_**	-0.310679612	**21.4660194**	-32.29703	16.9
**2013**	Forest	**324_13L1_**	-0.310679612	**6.46601942**	-5.2970297	17.6
**2013**	Forest	**324_13L1_**	-0.310679612	**15.4660194**	98.70297	15.3
**2013**	Forest	**328_13L1_**	1.689320388	**-21.5339806**	14.70297	15.9
**2013**	Forest	**328_13L1_**	1.689320388	**-35.5339806**	55.70297	15.1
**2013**	Forest	**328_13L1_**	1.689320388	**-40.5339806**	91.70297	17.7
**2013**	Forest	**330_13L1_**	-1.310679612	**13.4660194**	-54.29703	13.5
**2013**	Forest	**330_13L1_**	-1.310679612	**-0.53398058**	41.70297	13.1
**2013**	Forest	**330_13L1_**	-1.310679612	**1.46601942**	66.70297	13.4
**2013**	Forest	**334_13L1_**	1.689320388	**-12.5339806**	-57.29703	17
**2013**	Forest	**334_13L1_**	1.689320388	**-11.5339806**	-42.29703	16.5
**2013**	Forest	**334_13L1_**	1.689320388	**12.4660194**	-25.29703	15.8
**2013**	Forest	**335_13L1_**	-2.310679612	**14.4660194**	-55.29703	17.1
**2013**	Forest	**335_13L1_**	-2.310679612	**7.46601942**	-45.29703	17.3
**2013**	Forest	**335_13L1_**	-2.310679612	**1.46601942**	-17.29703	17.7
**2013**	Forest	**343_13L1_**	4.689320388	**-2.53398058**	-64.29703	15.7
**2013**	Forest	**343_13L1_**	4.689320388	**-9.53398058**	-63.29703	16
**2013**	Forest	**343_13L1_**	4.689320388	**4.46601942**	-42.29703	14.7
**2013**	Forest	**344_13L1_**	-1.310679612	**-15.5339806**	-17.29703	17
**2013**	Forest	**344_13L1_**	-1.310679612	**0.46601942**	5.7029703	17.1
**2013**	Forest	**344_13L1_**	-1.310679612	**8.46601942**	33.70297	18.8
**2013**	Forest	**344_13L1_**	-1.310679612	**-12.5339806**		16
**2013**	Forest	**1_13L2_**	-3.310679612	**-1.53398058**	-16.29703	17.3
**2013**	Forest	**1_13L2_**	-3.310679612	**-16.5339806**	38.70297	15.8
**2013**	Forest	**1_13L2_**	-3.310679612	**-7.53398058**		15.5
**2013**	Forest	**19_13L2_**	-3.310679612	**0.46601942**	1.7029703	19.4
**2013**	Forest	**19_13L2_**	-3.310679612	**3.46601942**	1.7029703	18.6
**2013**	Forest	**19_13L2_**	-3.310679612	**19.4660194**	71.70297	18.1
**2013**	Forest	**33_13L2_**	-2.310679612	**-7.53398058**	-50.29703	17.3
**2013**	Forest	**33_13L2_**	-2.310679612	**-2.53398058**	16.70297	19.3
**2013**	Forest	**33_13L2_**	-2.310679612	**-3.53398058**	32.70297	18.1
**2013**	Forest	**36_13L2_**	-3.310679612	**4.46601942**	-12.29703	17.5
**2013**	Forest	**36_13L2_**	-3.310679612	**-3.53398058**	9.7029703	18.2
**2013**	Forest	**36_13L2_**	-3.310679612	**-17.5339806**	37.70297	17.6
**2013**	Forest	**47_13L2_**	-0.310679612	**17.4660194**	-48.29703	15.4
**2013**	Forest	**47_13L2_**	-0.310679612	**22.4660194**	-45.29703	16.2
**2013**	Forest	**47_13L2_**	-0.310679612	**14.4660194**	60.70297	14.9
**2013**	Park	**5_13b_**	-1.130177515	**-2.18934911**	-95.94012	15.7
**2013**	Park	**5_13b_**	-1.130177515	**-16.1893491**	-22.94012	16.7
**2013**	Park	**5_13b_**	-1.130177515	**-26.1893491**	-20.94012	15.8
**2013**	Park	**12_13b_**	1.869822485	**-22.1893491**	-48.94012	14.4
**2013**	Park	**12_13b_**	1.869822485	**-31.1893491**	28.05988	14.6
**2013**	Park	**12_13b_**	1.869822485	**-26.1893491**	29.05988	14.3
**2013**	Park	**15_13b_**	2.869822485	**11.8106509**	-49.94012	16.3
**2013**	Park	**15_13b_**	2.869822485	**5.81065089**	26.05988	16.9
**2013**	Park	**15_13b_**	2.869822485	**6.81065089**	47.05988	15.5
**2013**	Park	**21_13b_**	9.869822485	**-8.18934911**	-54.94012	11.5
**2013**	Park	**21_13b_**	9.869822485	**-3.18934911**	-50.94012	11.6
**2013**	Park	**21_13b_**	9.869822485	**-19.1893491**	-9.9401198	9
**2013**	Park	**22_13b_**	0.869822485	**16.8106509**	-45.94012	14
**2013**	Park	**22_13b_**	0.869822485	**26.8106509**	-42.94012	12.7
**2013**	Park	**22_13b_**	0.869822485	**9.81065089**	27.05988	14.6

**2013**	Park	**25_13b_**	9.869822485	**1.81065089**	96.05988	18.1
**2013**	Park	**28_13b_**	0.869822485	**-35.1893491**	16.05988	14.9
**2013**	Park	**28_13b_**	0.869822485	**-40.1893491**	86.05988	12.3
**2013**	Park	**28_13b_**	0.869822485	**-35.1893491**	114.05988	13.7
**2013**	Park	**29_13b_**	0.869822485	**-12.1893491**	-38.94012	16.3
**2013**	Park	**29_13b_**	0.869822485	**-19.1893491**	-5.9401198	17.1
**2013**	Park	**29_13b_**	0.869822485	**-10.1893491**	26.05988	17.5
**2013**	Park	**30_13b_**	-1.130177515	**2.81065089**	-48.94012	15.2
**2013**	Park	**30_13b_**	-1.130177515	**0.81065089**	-17.94012	15.7
**2013**	Park	**30_13b_**	-1.130177515	**18.8106509**	-11.94012	17
**2013**	Park	**32_13b_**	0.869822485	**19.8106509**	15.05988	16.5
**2013**	Park	**32_13b_**	0.869822485	**6.81065089**	46.05988	17.1
**2013**	Park	**32_13b_**	0.869822485	**14.8106509**	72.05988	16.4
**2013**	Park	**34_13b_**	2.869822485	**-25.1893491**	-15.94012	12.3
**2013**	Park	**34_13b_**	2.869822485	**-30.1893491**	52.05988	11.7
**2013**	Park	**34_13b_**	2.869822485	**-4.18934911**	83.05988	13.7
**2013**	Park	**37_13b_**	0.869822485	**5.81065089**	-63.94012	17.8
**2013**	Park	**37_13b_**	0.869822485	**16.8106509**	-60.94012	19.1
**2013**	Park	**37_13b_**	0.869822485	**15.8106509**	23.05988	18.3
**2013**	Park	**42_13b_**	5.869822485	**7.81065089**	-44.94012	13.4
**2013**	Park	**42_13b_**	5.869822485	**-1.18934911**	-41.94012	14.4
**2013**	Park	**42_13b_**	5.869822485	**-1.18934911**	9.0598802	14.5
**2013**	Park	**43_13b_**	-0.130177515	**4.81065089**	-74.94012	15
**2013**	Park	**43_13b_**	-0.130177515	**-14.1893491**	-13.94012	15.6
**2013**	Park	**43_13b_**	-0.130177515	**-4.18934911**	50.05988	16.1
**2013**	Park	**43_13b_**	-0.130177515	**-12.1893491**		15.4
**2013**	Park	**46_13b_**	-4.130177515	**-2.18934911**	-45.94012	18.2
**2013**	Park	**46_13b_**	-4.130177515	**14.8106509**	-37.94012	16.6
**2013**	Park	**46_13b_**	-4.130177515	**12.8106509**	3.0598802	17.9
**2013**	Park	**49_13b_**	-0.130177515	**23.8106509**	-87.94012	16.7
**2013**	Park	**49_13b_**	-0.130177515	**25.8106509**	-71.94012	17.5
**2013**	Park	**49_13b_**	-0.130177515	**15.8106509**	-63.94012	16.1
**2013**	Park	**53_13b_**	1.869822485	**-17.1893491**	62.05988	16.8
**2013**	Park	**53_13b_**	1.869822485	**-19.1893491**	89.05988	16.2
**2013**	Park	**53_13b_**	1.869822485	**-21.1893491**	110.05988	16.6
**2013**	Park	**55_13b_**	-4.130177515	**0.81065089**	-19.94012	16
**2013**	Park	**55_13b_**	-4.130177515	**-17.1893491**	-17.94012	18
**2013**	Park	**55_13b_**	-4.130177515	**11.8106509**	41.05988	17.2
**2013**	Park	**59_13b_**	-2.130177515	**9.81065089**	24.05988	16.6
**2013**	Park	**59_13b_**	-2.130177515	**10.8106509**	25.05988	18.5
**2013**	Park	**59_13b_**	-2.130177515	**15.8106509**	30.05988	17.2
**2013**	Park	**61_13b_**	1.869822485	**-0.18934911**	-55.94012	18.5
**2013**	Park	**61_13b_**	1.869822485	**19.8106509**	-46.94012	19.6
**2013**	Park	**61_13b_**	1.869822485	**-4.18934911**	37.05988	18.5
**2013**	Park	**64_13b_**	-1.130177515	**10.8106509**	29.05988	15.3
**2013**	Park	**64_13b_**	-1.130177515	**9.81065089**	41.05988	17.3
**2013**	Park	**64_13b_**	-1.130177515	**2.81065089**	48.05988	17.2
**2013**	Park	**65_13b_**	-0.130177515	**0.81065089**	17.05988	19.7
**2013**	Park	**65_13b_**	-0.130177515	**-9.18934911**	66.05988	17.1
**2013**	Park	**65_13b_**	-0.130177515	**12.8106509**	92.05988	18.1
**2013**	Park	**66_13b_**	3.869822485	**14.8106509**	-43.94012	16.3
**2013**	Park	**66_13b_**	3.869822485	**-4.18934911**	-21.94012	15.4
**2013**	Park	**66_13b_**	3.869822485	**0.81065089**	-15.94012	14.9
**2013**	Park	**69_13b_**	-0.130177515	**-22.1893491**	104.05988	16.2

**2013**	Park	**69_13b_**	-0.130177515	**-20.1893491**	110.05988	17.3
**2013**	Park	**69_13b_**	-0.130177515	**-19.1893491**	118.05988	15.8
**2013**	Park	**72_13b_**	-0.130177515	**-6.18934911**	17.05988	16.4
**2013**	Park	**72_13b_**	-0.130177515	**1.81065089**	18.05988	15.2
**2013**	Park	**72_13b_**	-0.130177515	**-1.18934911**	62.05988	18.4
**2013**	Park	**76_13b_**	-1.130177515	**2.81065089**	-60.94012	16
**2013**	Park	**76_13b_**	-1.130177515	**2.81065089**	-23.94012	16.4
**2013**	Park	**76_13b_**	-1.130177515	**15.8106509**	-5.9401198	16.7
**2013**	Park	**80_13b_**	-1.130177515	**6.81065089**	-72.94012	16.4
**2013**	Park	**80_13b_**	-1.130177515	**5.81065089**	-28.94012	17.3
**2013**	Park	**80_13b_**	-1.130177515	**-8.18934911**	-22.94012	16.8
**2013**	Park	**84_13b_**	-2.130177515	**2.81065089**	12.05988	17
**2013**	Park	**84_13b_**	-2.130177515	**14.8106509**	32.05988	16.3
**2013**	Park	**84_13b_**	-2.130177515	**14.8106509**		14
**2013**	Park	**85_13b_**	0.869822485	**2.81065089**	11.05988	18.7
**2013**	Park	**85_13b_**	0.869822485	**8.81065089**	83.05988	19.7
**2013**	Park	**85_13b_**	0.869822485	**35.8106509**	98.05988	17.4
**2013**	Park	**90_13b_**	-2.130177515	**6.81065089**	-38.94012	15.4
**2013**	Park	**90_13b_**	-2.130177515	**3.81065089**	-37.94012	18.9
**2013**	Park	**90_13b_**	-2.130177515	**9.81065089**	-12.94012	17.4
**2013**	Park	**92_13b_**	0.869822485	**45.8106509**	-10.94012	16.4
**2013**	Park	**92_13b_**	0.869822485	**18.8106509**	21.05988	18.9
**2013**	Park	**92_13b_**	0.869822485	**-7.18934911**	33.05988	17.9
**2013**	Park	**93_13b_**	-0.130177515	**8.81065089**	-86.94012	14.6
**2013**	Park	**93_13b_**	-0.130177515	**27.8106509**	-46.94012	15.8
**2013**	Park	**93_13b_**	-0.130177515	**24.8106509**	54.05988	15.8
**2013**	Park	**95_13b_**	-1.130177515	**13.8106509**	-40.94012	15.4
**2013**	Park	**95_13b_**	-1.130177515	**11.8106509**	-31.94012	18.1
**2013**	Park	**95_13b_**	-1.130177515	**9.81065089**	-20.94012	17.5
**2013**	Park	**97_13b_**	-1.130177515	**3.81065089**	-71.94012	17.3
**2013**	Park	**97_13b_**	-1.130177515	**15.8106509**	-59.94012	16.6
**2013**	Park	**97_13b_**	-1.130177515	**16.8106509**	-54.94012	17.2
**2013**	Park	**99_13b_**	-0.130177515	**10.8106509**	-27.94012	15.2
**2013**	Park	**99_13b_**	-0.130177515	**-2.18934911**	-23.94012	14.4
**2013**	Park	**99_13b_**	-0.130177515	**-1.18934911**	76.05988	17
**2013**	Park	**100_13b_**	-4.130177515	**8.81065089**	-30.94012	16.1
**2013**	Park	**100_13b_**	-4.130177515	**2.81065089**	-13.94012	14.8
**2013**	Park	**100_13b_**	-4.130177515	**-14.1893491**	73.05988	16.6
**2013**	Park	**101_13b_**	2.869822485	**-1.18934911**	-21.94012	15
**2013**	Park	**101_13b_**	2.869822485	**-24.1893491**	32.05988	15
**2013**	Park	**101_13b_**	2.869822485	**-35.1893491**	54.05988	14.2
**2013**	Park	**107_13b_**	1.869822485	**5.81065089**	-56.94012	18.5
**2013**	Park	**107_13b_**	1.869822485	**10.8106509**	-41.94012	17
**2013**	Park	**107_13b_**	1.869822485	**9.81065089**	35.05988	17.1
**2013**	Park	**110_13b_**	-1.130177515	**17.8106509**	-50.94012	14.5
**2013**	Park	**110_13b_**	-1.130177515	**-3.18934911**	-35.94012	14.4
**2013**	Park	**110_13b_**	-1.130177515	**-9.18934911**	13.05988	15.2
**2013**	Park	**114_13b_**	-2.130177515	**8.81065089**	-15.94012	14.3
**2013**	Park	**114_13b_**	-2.130177515	**-6.18934911**	-1.9401198	13.9
**2013**	Park	**114_13b_**	-2.130177515	**4.81065089**	13.05988	14.6
**2013**	Park	**115_13b_**	-3.130177515	**-23.1893491**	-60.94012	14
**2013**	Park	**115_13b_**	-3.130177515	**-16.1893491**	-57.94012	13.1
**2013**	Park	**119_13b_**	-1.130177515	**5.81065089**	-85.94012	15.9
**2013**	Park	**119_13b_**	-1.130177515	**6.81065089**	-69.94012	14.9

**2013**	Park	**119_13b_**	-1.130177515	**0.81065089**	-52.94012	15.6
**2013**	Park	**124_13b_**	-1.130177515	**17.8106509**	-76.94012	13.7
**2013**	Park	**124_13b_**	-1.130177515	**10.8106509**	-37.94012	15.4
**2013**	Park	**124_13b_**	-1.130177515	**17.8106509**	45.05988	15.8
**2013**	Park	**125_13b_**	-2.130177515	**0.81065089**	-55.94012	17.2
**2013**	Park	**125_13b_**	-2.130177515	**20.8106509**	-54.94012	18.6
**2013**	Park	**125_13b_**	-2.130177515	**10.8106509**	13.05988	18.3
**2013**	Park	**9_13z_**	0.869822485	**10.8106509**	-68.94012	15.9
**2013**	Park	**9_13z_**	0.869822485	**1.81065089**	-36.94012	16.8
**2013**	Park	**9_13z_**	0.869822485	**5.81065089**	-6.9401198	16.8
**2013**	Park	**10_13z_**	-1.130177515	**-15.1893491**	-42.94012	17.4
**2013**	Park	**10_13z_**	-1.130177515	**4.81065089**	-34.94012	16.7
**2013**	Park	**10_13z_**	-1.130177515	**7.81065089**	17.05988	17.2
**2013**	Park	**14_13z_**	-1.130177515	**-21.1893491**	-79.94012	16.5
**2013**	Park	**14_13z_**	-1.130177515	**-13.1893491**	34.05988	17.4
**2013**	Park	**14_13z_**	-1.130177515	**-10.1893491**	76.05988	17.6
**2013**	Park	**16_13z_**	2.869822485	**-10.1893491**	29.05988	18.2
**2013**	Park	**16_13z_**	2.869822485	**-7.18934911**	52.05988	16.9
**2013**	Park	**16_13z_**	2.869822485	**-7.18934911**	61.05988	17.2
**2013**	Park	**22_13z_**	-3.130177515	**-23.1893491**	27.05988	15.1
**2013**	Park	**22_13z_**	-3.130177515	**-19.1893491**	33.05988	15.3
**2013**	Park	**22_13z_**	-3.130177515	**-19.1893491**	59.05988	14.2
**2013**	Park	**28_13z_**	2.869822485	**1.81065089**	-31.94012	18.4
**2013**	Park	**28_13z_**	2.869822485	**11.8106509**	24.05988	18.6
**2013**	Park	**28_13z_**	2.869822485	**8.81065089**	83.05988	18
**2013**	Park	**35_13z_**	-3.130177515	**-4.18934911**	11.05988	16.6
**2013**	Park	**35_13z_**	-3.130177515	**-6.18934911**	19.05988	16.2
**2013**	Park	**35_13z_**	-3.130177515	**-17.1893491**	101.05988	16.6
**2013**	Park	**39_13z_**	0.869822485	**10.8106509**	-55.94012	16.5
**2013**	Park	**39_13z_**	0.869822485	**14.8106509**	-49.94012	16.8
**2013**	Park	**39_13z_**	0.869822485	**-6.18934911**	84.05988	15.9
**2013**	Park	**44_13z_**	-3.130177515	**3.81065089**	-63.94012	17.1
**2013**	Park	**44_13z_**	-3.130177515	**-3.18934911**	-39.94012	18.3
**2013**	Park	**44_13z_**	-3.130177515	**8.81065089**	-5.9401198	16.7
**2013**	Park	**45_13z_**	-0.130177515	**9.81065089**	-9.9401198	15.6
**2013**	Park	**45_13z_**	-0.130177515	**0.81065089**	29.05988	15.7
**2013**	Park	**45_13z_**	-0.130177515	**-2.18934911**	48.05988	17.6
**2013**	Park	**48_13z_**	4.869822485	**1.81065089**	-20.94012	15.7
**2013**	Park	**48_13z_**	4.869822485	**-4.18934911**	-12.94012	15
**2013**	Park	**48_13z_**	4.869822485	**-10.1893491**	33.05988	14.5
**2013**	Park	**58_13z_**	0.869822485	**-18.1893491**	23.05988	16
**2013**	Park	**58_13z_**	0.869822485	**-16.1893491**	42.05988	16.6
**2013**	Park	**58_13z_**	0.869822485	**-19.1893491**	52.05988	14
**2013**	Park	**60_13z_**	-9.130177515	**-11.1893491**	-57.94012	14.6
**2013**	Park	**60_13z_**	-9.130177515	**-23.1893491**	3.0598802	14.1
**2013**	Park	**60_13z_**	-9.130177515	**-18.1893491**	10.05988	14.5
**2014**	Forest	**4_14L1_**	2.75	**0.24342105**	-42.724832	17
**2014**	Forest	**4_14L1_**	2.75	**-0.75657895**	-28.724832	18.1
**2014**	Forest	**4_14L1_**	2.75	**0.24342105**	-15.724832	18.2
**2014**	Forest	**7_14L1_**	1.75	**-7.75657895**	-63.724832	17.7
**2014**	Forest	**7_14L1_**	1.75	**-12.7565789**	-21.724832	17.1
**2014**	Forest	**7_14L1_**	1.75	**-20.7565789**	72.275168	16.4
**2014**	Forest	**20_14L1_**	-0.25	**-2.75657895**	-37.724832	18.2
**2014**	Forest	**20_14L1_**	-0.25	**-29.7565789**	-23.724832	15.9

**2014**	Forest	**20_14L1_**	-0.25	**-15.7565789**	-16.724832	17.2
**2014**	Forest	**31_14L1_**	-6.25	**10.2434211**	-2.7248322	16.1
**2014**	Forest	**31_14L1_**	-6.25	**13.2434211**	19.275168	16.5
**2014**	Forest	**31_14L1_**	-6.25	**3.24342105**	110.27517	16.9
**2014**	Forest	**34_14L1_**	-1.25	**-11.7565789**	-15.724832	18.3
**2014**	Forest	**34_14L1_**	-1.25	**-17.7565789**	-2.7248322	17.7
**2014**	Forest	**34_14L1_**	-1.25	**-14.7565789**	-2.7248322	16
**2014**	Forest	**53_14L1_**	1.75	**19.2434211**	-72.724832	18.2
**2014**	Forest	**53_14L1_**	1.75	**0.24342105**	-9.7248322	16.5
**2014**	Forest	**53_14L1_**	1.75	**-10.7565789**	39.275168	17.6
**2014**	Forest	**64_14L1_**	-1.25	**1.24342105**	-19.724832	16.9
**2014**	Forest	**64_14L1_**	-1.25	**-6.75657895**	12.275168	16.6
**2014**	Forest	**64_14L1_**	-1.25	**-13.7565789**	21.275168	17
**2014**	Forest	**68_14L1_**	1.75	**11.2434211**	-4.7248322	19.1
**2014**	Forest	**68_14L1_**	1.75	**-0.75657895**	2.2751678	16.3
**2014**	Forest	**68_14L1_**	1.75	**12.2434211**	53.275168	16.3
**2014**	Forest	**74_14L1_**	8.75	**-22.7565789**	-39.724832	16.6
**2014**	Forest	**74_14L1_**	8.75	**-20.7565789**	55.275168	16.7
**2014**	Forest	**74_14L1_**	8.75	**-0.75657895**	59.275168	17.9
**2014**	Forest	**76_14L1_**	-0.25	**-2.75657895**	-34.724832	16.8
**2014**	Forest	**76_14L1_**	-0.25	**-1.75657895**	-11.724832	16.4
**2014**	Forest	**76_14L1_**	-0.25	**5.24342105**	1.2751678	17.1
**2014**	Forest	**80_14L1_**	6.75	**-0.75657895**	-46.724832	16.9
**2014**	Forest	**80_14L1_**	6.75	**-7.75657895**	30.275168	17.4
**2014**	Forest	**84_14L1_**	11.75	**-15.7565789**	-20.724832	16.2
**2014**	Forest	**84_14L1_**	11.75	**-13.7565789**	-9.7248322	16
**2014**	Forest	**84_14L1_**	11.75	**-14.7565789**	5.2751678	16.3
**2014**	Forest	**93_14L1_**	-4.25	**-13.7565789**	-31.724832	
**2014**	Forest	**93_14L1_**	-4.25	**-8.75657895**	-31.724832	
**2014**	Forest	**93_14L1_**	-4.25	**-1.75657895**	61.275168	
**2014**	Forest	**99_14L1_**	0.75	**-13.7565789**	-26.724832	16.1
**2014**	Forest	**99_14L1_**	0.75	**-22.7565789**	-5.7248322	15.9
**2014**	Forest	**99_14L1_**	0.75	**-25.7565789**	16.275168	16.4
**2014**	Forest	**101_14L1_**	-7.25	**-6.75657895**	2.2751678	17
**2014**	Forest	**101_14L1_**	-7.25	**-10.7565789**	73.275168	16.9
**2014**	Forest	**103_14L1_**	-0.25	**-12.7565789**	-18.724832	17.2
**2014**	Forest	**103_14L1_**	-0.25	**0.24342105**	10.275168	16.5
**2014**	Forest	**103_14L1_**	-0.25	**-1.75657895**	54.275168	16.1
**2014**	Forest	**105_14L1_**	-4.25	**12.2434211**	-101.72483	16.8
**2014**	Forest	**105_14L1_**	-4.25	**12.2434211**	-22.724832	18.2
**2014**	Forest	**107_14L1_**	2.75	**-10.7565789**	1.2751678	17.9
**2014**	Forest	**107_14L1_**	2.75	**-16.7565789**	2.2751678	16.9
**2014**	Forest	**107_14L1_**	2.75	**-19.7565789**	107.27517	18.8
**2014**	Forest	**108_14L1_**	-3.25	**-8.75657895**	-9.7248322	16.3
**2014**	Forest	**108_14L1_**	-3.25	**6.24342105**	-5.7248322	17
**2014**	Forest	**108_14L1_**	-3.25	**9.24342105**	57.275168	17.3
**2014**	Forest	**109_14L1_**	-1.25	**4.24342105**	-18.724832	15.5
**2014**	Forest	**109_14L1_**	-1.25	**2.24342105**	-8.7248322	15
**2014**	Forest	**109_14L1_**	-1.25	**-11.7565789**	63.275168	15.5
**2014**	Forest	**109_14L1_**	-1.25	**15.2434211**		16.5
**2014**	Forest	**117_14L1_**	-0.25	**-11.7565789**	-17.724832	15.2
**2014**	Forest	**117_14L1_**	-0.25	**-27.7565789**	-5.7248322	15.9
**2014**	Forest	**117_14L1_**	-0.25	**-22.7565789**	23.275168	17.3
**2014**	Forest	**121_14L1_**	-0.25	**6.24342105**	-46.724832	19.1

**2014**	Forest	**121_14L1_**	-0.25	**18.2434211**	-46.724832	18.6
**2014**	Forest	**121_14L1_**	-0.25	**7.24342105**	8.2751678	18.9
**2014**	Forest	**135_14L1_**	4.75	**12.2434211**	-41.724832	17.2
**2014**	Forest	**135_14L1_**	4.75	**5.24342105**	-32.724832	16.6
**2014**	Forest	**135_14L1_**	4.75	**17.2434211**	5.2751678	17.2
**2014**	Forest	**147_14L1_**	2.75	**19.2434211**	-36.724832	17.1
**2014**	Forest	**147_14L1_**	2.75	**15.2434211**	-20.724832	19.1
**2014**	Forest	**147_14L1_**	2.75	**8.24342105**	10.275168	18.8
**2014**	Forest	**149_14L1_**	-3.25	**0.24342105**	-60.724832	16.9
**2014**	Forest	**149_14L1_**	-3.25	**1.24342105**	-22.724832	16.7
**2014**	Forest	**149_14L1_**	-3.25	**-10.7565789**	9.2751678	18.4
**2014**	Forest	**165_14L1_**	-1.25	**1.24342105**	-40.724832	16.3
**2014**	Forest	**165_14L1_**	-1.25	**1.24342105**	-29.724832	18.3
**2014**	Forest	**165_14L1_**	-1.25	**-3.75657895**	-23.724832	17.4
**2014**	Forest	**169_14L1_**	-1.25	**34.2434211**	-35.724832	17.9
**2014**	Forest	**169_14L1_**	-1.25	**4.24342105**	41.275168	18.1
**2014**	Forest	**169_14L1_**	-1.25	**0.24342105**	52.275168	18.7
**2014**	Forest	**170_14L1_**	-7.25	**6.24342105**	2.2751678	17.7
**2014**	Forest	**170_14L1_**	-7.25	**7.24342105**	27.275168	16.6
**2014**	Forest	**170_14L1_**	-7.25	**-9.75657895**	53.275168	17.6
**2014**	Forest	**171_14L1_**	0.75	**0.24342105**	-36.724832	15.9
**2014**	Forest	**171_14L1_**	0.75	**8.24342105**	14.275168	15.9
**2014**	Forest	**171_14L1_**	0.75	**11.2434211**	23.275168	14.9
**2014**	Forest	**179_14L1_**	4.75	**24.2434211**	-47.724832	18.2
**2014**	Forest	**179_14L1_**	4.75	**5.24342105**	-18.724832	17.4
**2014**	Forest	**179_14L1_**	4.75	**9.24342105**	10.275168	18
**2014**	Forest	**183_14L1_**	-1.25	**-11.7565789**	-11.724832	15.8
**2014**	Forest	**183_14L1_**	-1.25	**-6.75657895**	6.2751678	16.7
**2014**	Forest	**183_14L1_**	-1.25	**0.24342105**	42.275168	16.2
**2014**	Forest	**196_14L1_**	0.75	**3.24342105**	-12.724832	16.5
**2014**	Forest	**196_14L1_**	0.75	**8.24342105**	40.275168	15.7
**2014**	Forest	**196_14L1_**	0.75	**-5.75657895**		17.3
**2014**	Forest	**302_14L1_**	-3.25	**-6.75657895**	-30.724832	15.6
**2014**	Forest	**302_14L1_**	-3.25	**-20.7565789**	-6.7248322	15.7
**2014**	Forest	**302_14L1_**	-3.25	**-10.7565789**	16.275168	16.1
**2014**	Forest	**302_14L1_**	-1.25	**37.2434211**	27.275168	16
**2014**	Forest	**302_14L1_**	-1.25	**11.2434211**	49.275168	17.2
**2014**	Forest	**302_14L1_**	-1.25	**25.2434211**	62.275168	16.6
**2014**	Forest	**302_14L1_**	-1.25	**19.2434211**	76.275168	17.2
**2014**	Forest	**307_14L1_**	0.75	**-8.75657895**	62.275168	17.9
**2014**	Forest	**307_14L1_**	0.75	**5.24342105**	87.275168	16.8
**2014**	Forest	**307_14L1_**	0.75	**20.2434211**	103.27517	17.9
**2014**	Forest	**310_14L1_**	-2.25	**24.2434211**	-14.724832	17.8
**2014**	Forest	**310_14L1_**	-2.25	**3.24342105**	-13.724832	17.1
**2014**	Forest	**310_14L1_**	-2.25	**0.24342105**	14.275168	18.4
**2014**	Forest	**313_14L1_**	1.75	**7.24342105**	-32.724832	17.2
**2014**	Forest	**313_14L1_**	1.75	**8.24342105**	-11.724832	18.5
**2014**	Forest	**313_14L1_**	1.75	**-7.75657895**	-0.7248322	18.4
**2014**	Forest	**315_14L1_**	5.75	**-2.75657895**	-33.724832	17.3
**2014**	Forest	**315_14L1_**	5.75	**2.24342105**	-22.724832	17.3
**2014**	Forest	**315_14L1_**	5.75	**17.2434211**	-12.724832	17.6
**2014**	Forest	**321_14L1_**	-3.25	**4.24342105**	-18.724832	16.9
**2014**	Forest	**321_14L1_**	-3.25	**14.2434211**	-17.724832	16.1
**2014**	Forest	**321_14L1_**	-3.25	**17.2434211**	-9.7248322	17.2

**2014**	Forest	**333_14L1_**	1.75	**4.24342105**	-11.724832	18.5
**2014**	Forest	**333_14L1_**	1.75	**20.2434211**	11.275168	19.7
**2014**	Forest	**333_14L1_**	1.75	**10.2434211**	15.275168	19.8
**2014**	Forest	**335_14L1_**	-2.25	**14.2434211**	-48.724832	16.8
**2014**	Forest	**335_14L1_**	-2.25	**6.24342105**	13.275168	18.4
**2014**	Forest	**335_14L1_**	-2.25	**6.24342105**	16.275168	16.6
**2014**	Forest	**341_14L1_**	-1.25	**4.24342105**	-46.724832	17.4
**2014**	Forest	**341_14L1_**	-1.25	**9.24342105**	0.2751678	17.5
**2014**	Forest	**341_14L1_**	-1.25	**17.2434211**	0.2751678	17.2
**2014**	Forest	**344_14L1_**	0.75	**-2.75657895**	14.275168	17.4
**2014**	Forest	**344_14L1_**	0.75	**4.24342105**	17.275168	18.5
**2014**	Forest	**344_14L1_**	0.75	**-4.75657895**	70.275168	16.8
**2014**	Forest	**350_14L1_**	-0.25	**10.2434211**	-51.724832	17.6
**2014**	Forest	**350_14L1_**	-0.25	**-0.75657895**	13.275168	16.9
**2014**	Forest	**350_14L1_**	-0.25	**42.2434211**	27.275168	18.6
**2014**	Forest	**10_14L2_**	2.75	**1.24342105**	-28.724832	15.4
**2014**	Forest	**10_14L2_**	2.75	**-2.75657895**	-25.724832	15.8
**2014**	Forest	**10_14L2_**	2.75	**5.24342105**	-24.724832	17
**2014**	Forest	**11_14L2_**	-1.25	**-1.75657895**	-23.724832	16.8
**2014**	Forest	**11_14L2_**	-1.25	**-11.7565789**	-12.724832	17.5
**2014**	Forest	**11_14L2_**	-1.25	**-8.75657895**	13.275168	16.6
**2014**	Forest	**15_14L2_**	-2.25	**7.24342105**	1.2751678	16.6
**2014**	Forest	**15_14L2_**	-2.25	**13.2434211**	6.2751678	15.9
**2014**	Forest	**15_14L2_**	-2.25	**-0.75657895**	58.275168	15.8
**2014**	Forest	**33_14L2_**	-2.25	**-21.7565789**	-19.724832	15.2
**2014**	Forest	**33_14L2_**	-2.25	**-10.7565789**	-15.724832	16.7
**2014**	Forest	**33_14L2_**	-2.25	**-1.75657895**	-10.724832	16.1
**2014**	Forest	**35_14L2_**	0.75	**-10.7565789**	-37.724832	15.6
**2014**	Forest	**35_14L2_**	0.75	**4.24342105**	-12.724832	17.3
**2014**	Forest	**35_14L2_**	0.75	**2.24342105**	-5.7248322	16.2
**2014**	Forest	**36_14L2_**	4.75	**-31.7565789**	1.2751678	15.7
**2014**	Forest	**36_14L2_**	4.75	**-28.7565789**	46.275168	16.2
**2014**	Forest	**36_14L2_**	4.75	**-13.7565789**		14.9
**2014**	Forest	**47_14L2_**	-8.25	**5.24342105**	-47.724832	14.2
**2014**	Forest	**47_14L2_**	-8.25	**-6.75657895**	-41.724832	14.6
**2014**	Forest	**47_14L2_**	-8.25	**4.24342105**	-14.724832	15.5
**2014**	Park	**1_14b_**	-6.014563107	**7.33170732**	-54.352332	16.8
**2014**	Park	**1_14b_**	-6.014563107	**46.3317073**	-53.352332	15.7
**2014**	Park	**1_14b_**	-6.014563107	**24.3317073**	-38.352332	16.3
**2014**	Park	**2_14b_**	-2.014563107	**9.33170732**	-45.352332	16.9
**2014**	Park	**2_14b_**	-2.014563107	**-4.66829268**	31.647668	15.6
**2014**	Park	**2_14b_**	-2.014563107	**-14.6682927**	92.647668	15.8
**2014**	Park	**8_14b_**	-1.014563107	**-6.66829268**	-17.352332	13.9
**2014**	Park	**8_14b_**	-1.014563107	**-20.6682927**	70.647668	15.4
**2014**	Park	**8_14b_**	-1.014563107	**-13.6682927**	90.647668	14.9
**2014**	Park	**11_14b_**	-1.014563107	**4.33170732**	-19.352332	15.5
**2014**	Park	**11_14b_**	-1.014563107		-12.352332	17
**2014**	Park	**11_14b_**	-1.014563107	**8.33170732**	5.6476684	15.3
**2014**	Park	**11_14b_**	-1.014563107	**-3.66829268**		15.2
**2014**	Park	**13_14b_**	-4.014563107	**-13.6682927**	-42.352332	16.3
**2014**	Park	**13_14b_**	-4.014563107	**5.33170732**	-35.352332	16.2
**2014**	Park	**13_14b_**	-4.014563107	**10.3317073**	32.647668	16.6
**2014**	Park	**15_14b_**	-5.014563107	**22.3317073**	-70.352332	16.3
**2014**	Park	**15_14b_**	-5.014563107	**17.3317073**	-51.352332	15.5

**2014**	Park	**15_14b_**	-5.014563107	**13.3317073**	-31.352332	15.5
**2014**	Park	**16_14b_**	-3.014563107	**4.33170732**	-72.352332	16.4
**2014**	Park	**16_14b_**	-3.014563107	**1.33170732**	-44.352332	14.2
**2014**	Park	**16_14b_**	-3.014563107	**-4.66829268**	-32.352332	15.1
**2014**	Park	**22_14b_**	0.985436893	**-10.6682927**	-70.352332	16.3
**2014**	Park	**22_14b_**	0.985436893	**-0.66829268**	-53.352332	14.3
**2014**	Park	**22_14b_**	0.985436893	**-12.6682927**	-25.352332	16.2
**2014**	Park	**23_14b_**	-1.014563107	**-8.66829268**	-59.352332	14.1
**2014**	Park	**23_14b_**	-1.014563107	**18.3317073**	-50.352332	16.2
**2014**	Park	**23_14b_**	-1.014563107	**-8.66829268**	35.647668	16.8
**2014**	Park	**24_14b_**	-2.014563107	**-21.6682927**	29.647668	15.5
**2014**	Park	**24_14b_**	-2.014563107	**-12.6682927**	41.647668	16.9
**2014**	Park	**24_14b_**	-2.014563107	**-16.6682927**	81.647668	16.4
**2014**	Park	**25_14b_**	9.985436893	**-41.6682927**	11.647668	13.5
**2014**	Park	**25_14b_**	9.985436893	**-27.6682927**	40.647668	14.2
**2014**	Park	**25_14b_**	9.985436893	**-27.6682927**	45.647668	15.5
**2014**	Park	**28_14b_**	-2.014563107	**21.3317073**	-121.35233	12.1
**2014**	Park	**28_14b_**	-2.014563107	**42.3317073**	-90.352332	12.7
**2014**	Park	**28_14b_**	-2.014563107	**12.3317073**	-78.352332	12.9
**2014**	Park	**30_14b_**	-1.014563107	**9.33170732**	-57.352332	15.7
**2014**	Park	**30_14b_**	-1.014563107	**-2.66829268**	-36.352332	16.7
**2014**	Park	**30_14b_**	-1.014563107	**-3.66829268**	33.647668	16
**2014**	Park	**32_14b_**	1.985436893	**6.33170732**	-4.3523316	16.7
**2014**	Park	**32_14b_**	1.985436893	**-2.66829268**	6.6476684	17.9
**2014**	Park	**32_14b_**	1.985436893	**-3.66829268**	39.647668	17.5
**2014**	Park	**33_14b_**	3.985436893	**22.3317073**	-77.352332	15.6
**2014**	Park	**33_14b_**	3.985436893	**20.3317073**	-36.352332	15.6
**2014**	Park	**33_14b_**	3.985436893	**41.3317073**	21.647668	16.8
**2014**	Park	**34_14b_**	6.985436893	**-26.6682927**	-1.3523316	14.5
**2014**	Park	**34_14b_**	6.985436893	**-17.6682927**	-1.3523316	15.2
**2014**	Park	**34_14b_**	6.985436893	**-35.6682927**	6.6476684	14.5
**2014**	Park	**35_14b_**	1.985436893	**-6.66829268**	-55.352332	16.8
**2014**	Park	**35_14b_**	1.985436893	**17.3317073**	-6.3523316	16.5
**2014**	Park	**35_14b_**	1.985436893	**0.33170732**	-4.3523316	16.9
**2014**	Park	**39_14b_**	-3.014563107	**10.3317073**	-55.352332	14.6
**2014**	Park	**39_14b_**	-3.014563107	**20.3317073**	-33.352332	14.9
**2014**	Park	**39_14b_**	-3.014563107	**14.3317073**	48.647668	14.9
**2014**	Park	**41_14b_**	-1.014563107	**-31.6682927**	0.6476684	12.4
**2014**	Park	**41_14b_**	-1.014563107	**-54.6682927**	61.647668	16.6
**2014**	Park	**41_14b_**	-1.014563107	**-39.6682927**	101.64767	12.8
**2014**	Park	**43_14b_**	2.985436893	**-34.6682927**	-56.352332	14.1
**2014**	Park	**43_14b_**	2.985436893	**-20.6682927**	-44.352332	15.4
**2014**	Park	**43_14b_**	2.985436893	**-30.6682927**	16.647668	14.6
**2014**	Park	**44_14b_**	-6.014563107	**22.3317073**	-41.352332	16.2
**2014**	Park	**44_14b_**	-6.014563107	**33.3317073**	28.647668	18.7
**2014**	Park	**44_14b_**	-6.014563107	**15.3317073**	52.647668	17.9
**2014**	Park	**45_14b_**	-4.014563107	**13.3317073**	17.647668	14.7
**2014**	Park	**45_14b_**	-4.014563107	**-8.66829268**	53.647668	14
**2014**	Park	**45_14b_**	-4.014563107	**-12.6682927**	81.647668	13.5
**2014**	Park	**49_14b_**	4.985436893	**8.33170732**	-81.352332	15.3
**2014**	Park	**49_14b_**	4.985436893	**3.33170732**	-56.352332	13.6
**2014**	Park	**49_14b_**	4.985436893	**-21.6682927**	-2.3523316	12.5
**2014**	Park	**50_14b_**	4.985436893	**12.3317073**	-62.352332	15.3
**2014**	Park	**50_14b_**	4.985436893	**20.3317073**	-60.352332	17.6

**2014**	Park	**50_14b_**	4.985436893	**20.3317073**	-54.352332	15.6
**2014**	Park	**57_14b_**	-3.014563107	**-11.6682927**		16.2
**2014**	Park	**57_14b_**	-3.014563107	**-11.6682927**		16
**2014**	Park	**57_14b_**	-3.014563107	**0.33170732**		15.9
**2014**	Park	**58_14b_**	-4.014563107	**24.3317073**	-78.352332	17.8
**2014**	Park	**58_14b_**	-4.014563107	**14.3317073**	-52.352332	17.2
**2014**	Park	**58_14b_**	-4.014563107	**6.33170732**	-20.352332	17.9
**2014**	Park	**59_14b_**	2.985436893	**-6.66829268**	-3.3523316	16.5
**2014**	Park	**59_14b_**	2.985436893	**-6.66829268**	10.647668	15.4
**2014**	Park	**59_14b_**	2.985436893	**-10.6682927**	51.647668	15.9
**2014**	Park	**60_14b_**	-0.014563107	**15.3317073**	-41.352332	17.4
**2014**	Park	**60_14b_**	-0.014563107	**7.33170732**	38.647668	17.6
**2014**	Park	**60_14b_**	-0.014563107	**18.3317073**	48.647668	17.8
**2014**	Park	**61_14b_**	2.985436893	**-1.66829268**	1.6476684	16.5
**2014**	Park	**61_14b_**	2.985436893	**20.3317073**	26.647668	16.7
**2014**	Park	**61_14b_**	2.985436893	**2.33170732**	27.647668	17.5
**2014**	Park	**63_14b_**	2.985436893	**8.33170732**	-8.3523316	17.5
**2014**	Park	**63_14b_**	2.985436893	**-1.66829268**	21.647668	16.7
**2014**	Park	**63_14b_**	2.985436893	**28.3317073**	25.647668	16.6
**2014**	Park	**64_14b_**	2.985436893	**13.3317073**	-31.352332	16.5
**2014**	Park	**64_14b_**	2.985436893	**20.3317073**	-26.352332	17.1
**2014**	Park	**64_14b_**	2.985436893	**13.3317073**	-21.352332	17.9
**2014**	Park	**67_14b_**	-0.014563107	**3.33170732**	-45.352332	18.2
**2014**	Park	**67_14b_**	-0.014563107	**13.3317073**	40.647668	16.8
**2014**	Park	**67_14b_**	-0.014563107	**-11.6682927**	58.647668	18.3
**2014**	Park	**68_14b_**	-1.014563107	**22.3317073**	-19.352332	16.6
**2014**	Park	**68_14b_**	-1.014563107	**12.3317073**	34.647668	16.7
**2014**	Park	**68_14b_**	-1.014563107	**11.3317073**	72.647668	16.6
**2014**	Park	**70_14b_**	15.98543689	**4.33170732**	-9.3523316	14.9
**2014**	Park	**70_14b_**	15.98543689	**7.33170732**	51.647668	14.7
**2014**	Park	**70_14b_**	15.98543689	**10.3317073**	91.647668	14
**2014**	Park	**75_14b_**	-5.014563107	**6.33170732**	-68.352332	15.9
**2014**	Park	**75_14b_**	-5.014563107	**0.33170732**	12.647668	17.3
**2014**	Park	**75_14b_**	-5.014563107	**-4.66829268**	54.647668	16.3
**2014**	Park	**79_14b_**	-4.014563107	**-1.66829268**	-20.352332	18
**2014**	Park	**79_14b_**	-4.014563107	**-8.66829268**	26.647668	18.1
**2014**	Park	**79_14b_**	-4.014563107	**2.33170732**	37.647668	17.3
**2014**	Park	**81_14b_**	-4.014563107	**10.3317073**	-50.352332	13.9
**2014**	Park	**81_14b_**	-4.014563107	**-10.6682927**	-41.352332	15.1
**2014**	Park	**81_14b_**	-4.014563107	**12.3317073**	43.647668	13.6
**2014**	Park	**84_14b_**	-3.014563107	**-38.6682927**	-27.352332	12.4
**2014**	Park	**84_14b_**	-3.014563107	**-42.6682927**	-23.352332	12
**2014**	Park	**84_14b_**	-3.014563107	**-30.6682927**	-22.352332	12.5
**2014**	Park	**88_14b_**	-8.014563107	**20.3317073**	-64.352332	15.6
**2014**	Park	**88_14b_**	-8.014563107	**2.33170732**	-60.352332	14.9
**2014**	Park	**88_14b_**	-8.014563107	**18.3317073**	-19.352332	15.1
**2014**	Park	**91_14b_**	-2.014563107	**17.3317073**	-57.352332	
**2014**	Park	**91_14b_**	-2.014563107	**13.3317073**	-5.3523316	
**2014**	Park	**91_14b_**	-2.014563107	**1.33170732**	36.647668	
**2014**	Park	**92_14b_**	13.98543689	**17.3317073**	0.6476684	14.5
**2014**	Park	**92_14b_**	13.98543689	**-5.66829268**	18.647668	14.5
**2014**	Park	**92_14b_**	13.98543689	**17.3317073**	83.647668	13.9
**2014**	Park	**93_14b_**	2.985436893	**38.3317073**	-64.352332	14.8
**2014**	Park	**93_14b_**	2.985436893	**16.3317073**	-0.3523316	17.4

**2014**	Park	**93_14b_**	2.985436893	**19.3317073**	26.647668	17.2
**2014**	Park	**95_14b_**	-4.014563107	**-1.66829268**	36.647668	17.1
**2014**	Park	**95_14b_**	-4.014563107	**-3.66829268**	71.647668	17.1
**2014**	Park	**95_14b_**	-4.014563107	**-1.66829268**	75.647668	17.3
**2014**	Park	**100_14b_**	-6.014563107	**-0.66829268**	-62.352332	16.1
**2014**	Park	**100_14b_**	-6.014563107	**9.33170732**	-19.352332	15.2
**2014**	Park	**100_14b_**	-6.014563107	**-13.6682927**	-16.352332	15.9
**2014**	Park	**101_14b_**	-4.014563107	**15.3317073**	-35.352332	16.2
**2014**	Park	**101_14b_**	-4.014563107	**24.3317073**	38.647668	16.3
**2014**	Park	**101_14b_**	-4.014563107	**19.3317073**	57.647668	16.9
**2014**	Park	**102_14b_**	2.985436893	**0.33170732**	-47.352332	17.2
**2014**	Park	**102_14b_**	2.985436893	**16.3317073**	-11.352332	16.1
**2014**	Park	**102_14b_**	2.985436893	**-9.66829268**	3.6476684	15.7
**2014**	Park	**104_14b_**	-6.014563107	**0.33170732**	-54.352332	13.4
**2014**	Park	**104_14b_**	-6.014563107	**16.3317073**	10.647668	14.7
**2014**	Park	**104_14b_**	-6.014563107	**0.33170732**	50.647668	15.9
**2014**	Park	**107_14b_**	-1.014563107	**-20.6682927**	2.6476684	11.7
**2014**	Park	**107_14b_**	-1.014563107	**-19.6682927**	32.647668	14
**2014**	Park	**107_14b_**	-1.014563107	**-19.6682927**	65.647668	15.1
**2014**	Park	**108_14b_**	6.985436893	**0.33170732**	-55.352332	12.8
**2014**	Park	**108_14b_**	6.985436893	**9.33170732**	-42.352332	13.7
**2014**	Park	**108_14b_**	6.985436893	**34.3317073**	-27.352332	13.7
**2014**	Park	**111_14b_**	-3.014563107	**-15.6682927**		16.5
**2014**	Park	**111_14b_**	-3.014563107	**-5.66829268**		17.2
**2014**	Park	**111_14b_**	-3.014563107	**0.33170732**		15.9
**2014**	Park	**113_14b_**	-8.014563107	**3.33170732**	-29.352332	17.3
**2014**	Park	**113_14b_**	-8.014563107	**-7.66829268**	17.647668	16.4
**2014**	Park	**113_14b_**	-8.014563107	**4.33170732**	39.647668	17.7
**2014**	Park	**118_14b_**	-1.014563107	**-13.6682927**	-45.352332	17.6
**2014**	Park	**118_14b_**	-1.014563107	**-17.6682927**	-14.352332	16.6
**2014**	Park	**118_14b_**	-1.014563107	**10.3317073**	1.6476684	16.2
**2014**	Park	**121_14b_**	0.985436893	**12.3317073**	-67.352332	14.1
**2014**	Park	**121_14b_**	0.985436893	**15.3317073**	-37.352332	15.4
**2014**	Park	**121_14b_**	0.985436893	**8.33170732**		14.4
**2014**	Park	**121_14b_**	0.985436893	**17.3317073**		13.3
**2014**	Park	**126_14b_**	-6.014563107	**2.33170732**	-62.352332	14.8
**2014**	Park	**126_14b_**	-6.014563107	**21.3317073**	-37.352332	15.8
**2014**	Park	**126_14b_**	-6.014563107	**15.3317073**	58.647668	16.6
**2014**	Park	**650_14b_**	0.985436893	**28.3317073**	-51.352332	16.5
**2014**	Park	**650_14b_**	0.985436893	**22.3317073**	34.647668	16.8
**2014**	Park	**650_14b_**	0.985436893	**22.3317073**		15.6
**2014**	Park	**920_14b_**	-0.014563107	**14.3317073**	-4.3523316	16.7
**2014**	Park	**920_14b_**	-0.014563107	**-5.66829268**	17.647668	15.6
**2014**	Park	**920_14b_**	-0.014563107	**-4.66829268**	64.647668	16.2
**2014**	Park	**8_14z_**	-2.014563107	**-9.66829268**	-27.352332	14.7
**2014**	Park	**8_14z_**	-2.014563107	**-8.66829268**	40.647668	15.3
**2014**	Park	**8_14z_**	-2.014563107	**-15.6682927**	97.647668	16.5
**2014**	Park	**10_14z_**	-4.014563107	**15.3317073**		15
**2014**	Park	**10_14z_**	-4.014563107	**22.3317073**		13.6
**2014**	Park	**10_14z_**	-4.014563107	**30.3317073**		13.9
**2014**	Park	**14_14z_**	0.985436893	**-18.6682927**	25.647668	16.4
**2014**	Park	**14_14z_**	0.985436893	**-32.6682927**	41.647668	16
**2014**	Park	**14_14z_**	0.985436893	**-39.6682927**	121.64767	14.5
**2014**	Park	**16_14z_**	0.985436893	**-33.6682927**	-21.352332	16.3

**2014**	Park	**16_14z_**	0.985436893	**-24.6682927**	61.647668	16.7
**2014**	Park	**16_14z_**	0.985436893	**-30.6682927**	65.647668	15.6
**2014**	Park	**17_14z_**	0.985436893	**-20.6682927**	-29.352332	16.7
**2014**	Park	**17_14z_**	0.985436893	**-27.6682927**	-27.352332	17.6
**2014**	Park	**17_14z_**	0.985436893	**-15.6682927**	32.647668	16.2
**2014**	Park	**29_14z_**	7.985436893	**3.33170732**	-49.352332	13.5
**2014**	Park	**29_14z_**	7.985436893	**9.33170732**	6.6476684	14.8
**2014**	Park	**29_14z_**	7.985436893	**-25.6682927**	21.647668	13.5
**2014**	Park	**33_14z_**	1.985436893	**14.3317073**	-26.352332	17.4
**2014**	Park	**33_14z_**	1.985436893	**-26.6682927**	24.647668	17.1
**2014**	Park	**33_14z_**	1.985436893	**22.3317073**	34.647668	16.9
**2014**	Park	**39_14z_**	-2.014563107	**-25.6682927**	30.647668	16.9
**2014**	Park	**39_14z_**	-2.014563107	**-43.6682927**	58.647668	14.8
**2014**	Park	**39_14z_**	-2.014563107	**-29.6682927**	73.647668	16.8
**2014**	Park	**41_14z_**	5.985436893	**-1.66829268**	-49.352332	15.4
**2014**	Park	**41_14z_**	5.985436893	**1.33170732**	-26.352332	15.4
**2014**	Park	**41_14z_**	5.985436893	**-6.66829268**	10.647668	15.5
**2014**	Park	**44_14z_**	-3.014563107	**-21.6682927**	5.6476684	13.7
**2014**	Park	**44_14z_**	-3.014563107	**-10.6682927**	40.647668	13.8
**2014**	Park	**44_14z_**	-3.014563107	**-12.6682927**	57.647668	15.4
**2014**	Park	**53_14z_**	2.985436893	**24.3317073**	-2.3523316	16.2
**2014**	Park	**53_14z_**	2.985436893	**13.3317073**	55.647668	16.7
**2014**	Park	**53_14z_**	2.985436893	**44.3317073**	71.647668	17.4
**2014**	Park	**58_14z_**	8.985436893	**-45.6682927**	-32.352332	15.8
**2014**	Park	**58_14z_**	8.985436893	**-52.6682927**	52.647668	13.5
**2014**	Park	**58_14z_**	8.985436893	**-40.6682927**	52.647668	14.6
**2015**	Forest	**2_15L1_**	2.905325444	**2.75739645**	-16.482143	15.6
**2015**	Forest	**2_15L1_**	2.905325444	**-14.2426036**	-3.4821429	16.8
**2015**	Forest	**2_15L1_**	2.905325444	**-26.2426036**	71.517857	14.8
**2015**	Forest	**3_15L1_**	3.905325444	**-17.2426036**	-19.482143	18.9
**2015**	Forest	**3_15L1_**	3.905325444	**-7.24260355**	1.5178571	18.7
**2015**	Forest	**3_15L1_**	3.905325444	**-11.2426036**	16.517857	19.5
**2015**	Forest	**9_15L1_**	3.905325444	**6.75739645**	-52.482143	15.5
**2015**	Forest	**9_15L1_**	3.905325444	**-4.24260355**	-41.482143	17
**2015**	Forest	**9_15L1_**	3.905325444	**21.7573964**	-16.482143	18.1
**2015**	Forest	**13_15L1_**	0.905325444	**-22.2426036**	-20.482143	17.7
**2015**	Forest	**13_15L1_**	0.905325444	**-3.24260355**	-20.482143	17.2
**2015**	Forest	**13_15L1_**	0.905325444	**-27.2426036**	44.517857	17.7
**2015**	Forest	**19_15L1_**	1.905325444	**-8.24260355**	6.5178571	17
**2015**	Forest	**19_15L1_**	1.905325444	**3.75739645**	50.517857	18.9
**2015**	Forest	**19_15L1_**	1.905325444	**-7.24260355**	63.517857	15.7
**2015**	Forest	**24_15L1_**	-2.094674556	**17.7573964**	-65.482143	16.3
**2015**	Forest	**24_15L1_**	-2.094674556	**9.75739645**	-28.482143	17.6
**2015**	Forest	**24_15L1_**	-2.094674556	**3.75739645**	11.517857	15.3
**2015**	Forest	**30_15L_**	0.905325444	**-17.2426036**	14.517857	18
**2015**	Forest	**30_15L_**	0.905325444	**-3.24260355**	114.51786	18.4
**2015**	Forest	**30_15L_**	0.905325444	**1.75739645**	140.51786	19
**2015**	Forest	**40_15L1_**	-3.094674556	**-1.24260355**	-13.482143	17.8
**2015**	Forest	**40_15L1_**	-3.094674556	**-4.24260355**	-1.4821429	17.8
**2015**	Forest	**40_15L1_**	-3.094674556	**-6.24260355**	26.517857	18
**2015**	Forest	**48_15L1_**	1.905325444	**-14.2426036**	-25.482143	17.2
**2015**	Forest	**48_15L1_**	1.905325444	**-4.24260355**	13.517857	16.6
**2015**	Forest	**48_15L1_**	1.905325444	**-17.2426036**	159.51786	17.9
**2015**	Forest	**57_15L1_**	4.905325444	**31.7573964**	-49.482143	17.6

**2015**	Forest	**57_15L1_**	4.905325444	**3.75739645**	-29.482143	17.4
**2015**	Forest	**57_15L1_**	4.905325444	**7.75739645**	67.517857	18.1
**2015**	Forest	**68_15L1_**	-0.094674556	**-5.24260355**	55.517857	17.3
**2015**	Forest	**68_15L1_**	-0.094674556	**1.75739645**	58.517857	19.5
**2015**	Forest	**68_15L1_**	-0.094674556	**-10.2426036**	85.517857	17.3
**2015**	Forest	**80_15L1_**	-0.094674556	**19.7573964**	-33.482143	18.5
**2015**	Forest	**80_15L1_**	-0.094674556	**14.7573964**	-19.482143	18.2
**2015**	Forest	**80_15L1_**	-0.094674556	**19.7573964**	87.517857	19.2
**2015**	Forest	**82_15L1_**	1.905325444	**19.7573964**	2.5178571	19.4
**2015**	Forest	**82_15L1_**	1.905325444	**-6.24260355**	55.517857	18.6
**2015**	Forest	**82_15L1_**	1.905325444	**-0.24260355**	58.517857	19.2
**2015**	Forest	**84_15L1_**	2.905325444	**28.7573964**	-90.482143	17.4
**2015**	Forest	**84_15L1_**	2.905325444	**36.7573964**	-61.482143	18.8
**2015**	Forest	**84_15L1_**	2.905325444	**-6.24260355**	-48.482143	19.1
**2015**	Forest	**97_15L1_**	-4.094674556	**8.75739645**	-50.482143	17.7
**2015**	Forest	**97_15L1_**	-4.094674556	**2.75739645**	-35.482143	18.1
**2015**	Forest	**97_15L1_**	-4.094674556	**-2.24260355**		18.2
**2015**	Forest	**102_15L1_**	0.905325444	**-6.24260355**	21.517857	17.3
**2015**	Forest	**102_15L1_**	0.905325444	**3.75739645**	54.517857	17.4
**2015**	Forest	**102_15L1_**	0.905325444	**10.7573964**	55.517857	17.2
**2015**	Forest	**109_15L1_**	-2.094674556	**-9.24260355**	22.517857	16.6
**2015**	Forest	**109_15L1_**	-2.094674556	**-9.24260355**	29.517857	17.4
**2015**	Forest	**109_15L1_**	-2.094674556	**-14.2426036**	62.517857	16.7
**2015**	Forest	**113_15L1_**	-0.094674556	**23.7573964**	-48.482143	18.4
**2015**	Forest	**113_15L1_**	-0.094674556	**22.7573964**	83.517857	16.7
**2015**	Forest	**113_15L1_**	-0.094674556	**15.7573964**	95.517857	16.1
**2015**	Forest	**117_15L1_**	-0.094674556	**1.75739645**	-1.4821429	17.3
**2015**	Forest	**117_15L1_**	-0.094674556	**-5.24260355**	7.5178571	17.9
**2015**	Forest	**117_15L1_**	-0.094674556	**-19.2426036**	89.517857	17.1
**2015**	Forest	**123_15L1_**	-2.094674556	**-3.24260355**	-33.482143	17.2
**2015**	Forest	**123_15L1_**	-2.094674556	**-20.2426036**	8.5178571	15.6
**2015**	Forest	**123_15L1_**	-2.094674556	**1.75739645**	71.517857	16.3
**2015**	Forest	**127_15L1_**	-6.094674556	**4.75739645**	-56.482143	17.2
**2015**	Forest	**127_15L1_**	-6.094674556	**7.75739645**	-11.482143	17.1
**2015**	Forest	**127_15L1_**	-6.094674556	**9.75739645**	32.517857	16.4
**2015**	Forest	**128_15L1_**	-2.094674556	**11.7573964**	-61.482143	18
**2015**	Forest	**128_15L1_**	-2.094674556	**12.7573964**	-59.482143	16.8
**2015**	Forest	**128_15L1_**	-2.094674556	**6.75739645**	-31.482143	17.5
**2015**	Forest	**132_15L1_**	3.905325444	**-2.24260355**	-75.482143	18.2
**2015**	Forest	**132_15L1_**	3.905325444	**-7.24260355**	-40.482143	18.5
**2015**	Forest	**132_15L1_**	3.905325444	**-9.24260355**	-31.482143	18.8
**2015**	Forest	**136_15L1_**	3.905325444	**-14.2426036**	-44.482143	17.2
**2015**	Forest	**136_15L1_**	3.905325444	**5.75739645**	-42.482143	17.9
**2015**	Forest	**136_15L1_**	3.905325444	**-13.2426036**	-20.482143	17.8
**2015**	Forest	**138_15L1_**	-6.094674556	**-11.2426036**	4.5178571	17.2
**2015**	Forest	**138_15L1_**	-6.094674556	**-27.2426036**	11.517857	15.4
**2015**	Forest	**138_15L1_**	-6.094674556	**-7.24260355**	23.517857	16
**2015**	Forest	**140_15L1_**	3.905325444	**0.75739645**	-75.482143	16.2
**2015**	Forest	**140_15L1_**	3.905325444	**-0.24260355**	-37.482143	18.2
**2015**	Forest	**140_15L1_**	3.905325444	**-2.24260355**	-28.482143	17
**2015**	Forest	**146_15L1_**	4.905325444	**-42.2426036**	34.517857	16
**2015**	Forest	**146_15L1_**	4.905325444	**-36.2426036**	40.517857	17
**2015**	Forest	**146_15L1_**	4.905325444	**-56.2426036**	71.517857	16.5
**2015**	Forest	**152_15L1_**	-1.094674556	**-0.24260355**	-22.482143	16.6

**2015**	Forest	**152_15L1_**	-1.094674556	**-2.24260355**	-19.482143	16.9
**2015**	Forest	**152_15L1_**	-1.094674556	**-14.2426036**	58.517857	16.2
**2015**	Forest	**156_15L1_**	1.905325444	**-2.24260355**	-48.482143	17.1
**2015**	Forest	**156_15L1_**	1.905325444	**25.7573964**	27.517857	18
**2015**	Forest	**156_15L1_**	1.905325444	**16.7573964**	46.517857	16.5
**2015**	Forest	**158_15L1_**	-4.094674556	**14.7573964**	-33.482143	18.1
**2015**	Forest	**158_15L1_**	-4.094674556	**18.7573964**	31.517857	19.1
**2015**	Forest	**158_15L1_**	-4.094674556	**6.75739645**	101.51786	17.7
**2015**	Forest	**164_15L1_**	-0.094674556	**-0.24260355**	20.517857	17.4
**2015**	Forest	**164_15L1_**	-0.094674556	**12.7573964**	23.517857	17.5
**2015**	Forest	**164_15L1_**	-0.094674556	**2.75739645**	114.51786	17.5
**2015**	Forest	**166_15L1_**	-6.094674556	**-19.2426036**	-50.482143	16.5
**2015**	Forest	**166_15L1_**	-6.094674556	**-24.2426036**	20.517857	15.9
**2015**	Forest	**166_15L1_**	-6.094674556	**-27.2426036**	43.517857	16.7
**2015**	Forest	**167_15L1_**	1.905325444	**49.7573964**	-53.482143	19.4
**2015**	Forest	**167_15L1_**	1.905325444	**44.7573964**	-49.482143	19.5
**2015**	Forest	**167_15L1_**	1.905325444	**23.7573964**	-41.482143	17.6
**2015**	Forest	**175_15L1_**	5.905325444	**10.7573964**	-77.482143	17.1
**2015**	Forest	**175_15L1_**	5.905325444	**0.75739645**	-5.4821429	17.7
**2015**	Forest	**175_15L1_**	5.905325444	**2.75739645**	31.517857	17.7
**2015**	Forest	**186_15L1_**	-2.094674556	**14.7573964**	-39.482143	16.6
**2015**	Forest	**186_15L1_**	-2.094674556	**10.7573964**	-9.4821429	16.1
**2015**	Forest	**186_15L1_**	-2.094674556	**15.7573964**	69.517857	17.6
**2015**	Forest	**190_15L1_**	3.905325444	**11.7573964**	-46.482143	15.7
**2015**	Forest	**190_15L1_**	3.905325444	**6.75739645**	-26.482143	18
**2015**	Forest	**190_15L1_**	3.905325444	**9.75739645**	20.517857	16.7
**2015**	Forest	**303_15L1_**	-2.094674556	**27.7573964**	-52.482143	19.4
**2015**	Forest	**303_15L1_**	-2.094674556	**36.7573964**	-44.482143	16.5
**2015**	Forest	**303_15L1_**	-2.094674556	**15.7573964**	5.5178571	17.2
**2015**	Forest	**305_15L1_**	4.905325444	**4.75739645**	-17.482143	18.7
**2015**	Forest	**305_15L1_**	4.905325444	**-0.24260355**	48.517857	18.2
**2015**	Forest	**305_15L1_**	4.905325444	**32.7573964**	58.517857	18.9
**2015**	Forest	**309_15L1_**	-2.094674556	**9.75739645**	-37.482143	16.4
**2015**	Forest	**309_15L1_**	-2.094674556	**9.75739645**	37.517857	16.5
**2015**	Forest	**309_15L1_**	-2.094674556	**0.75739645**	43.517857	17.1
**2015**	Forest	**313_15L1_**	2.905325444	**29.7573964**	-50.482143	16.3
**2015**	Forest	**313_15L1_**	2.905325444	**23.7573964**	-38.482143	18.2
**2015**	Forest	**313_15L1_**	2.905325444	**31.7573964**	-20.482143	18.1
**2015**	Forest	**316_15L1_**	2.905325444	**18.7573964**	-72.482143	16
**2015**	Forest	**316_15L1_**	2.905325444	**15.7573964**	-32.482143	15.3
**2015**	Forest	**316_15L1_**	2.905325444	**-3.24260355**	-27.482143	16.9
**2015**	Forest	**319_15L1_**	2.905325444	**1.75739645**	-49.482143	18.5
**2015**	Forest	**319_15L1_**	2.905325444	**46.7573964**	-30.482143	18.3
**2015**	Forest	**319_15L1_**	2.905325444	**-12.2426036**	37.517857	20
**2015**	Forest	**328_15L1_**	1.905325444	**-15.2426036**	-49.482143	15.4
**2015**	Forest	**328_15L1_**	1.905325444	**-23.2426036**	-25.482143	17
**2015**	Forest	**328_15L1_**	1.905325444	**-30.2426036**	14.517857	15.3
**2015**	Forest	**329_15L1_**	-4.094674556	**54.7573964**	-52.482143	14.1
**2015**	Forest	**329_15L1_**	-4.094674556	**26.7573964**	-33.482143	15.2
**2015**	Forest	**329_15L1_**	-4.094674556	**39.7573964**	-33.482143	15.8
**2015**	Forest	**334_15L1_**	-2.094674556	**29.7573964**	-20.482143	18.7
**2015**	Forest	**334_15L1_**	-2.094674556	**14.7573964**	15.517857	19.5
**2015**	Forest	**334_15L1_**	-2.094674556	**9.75739645**	21.517857	19.1
**2015**	Forest	**350_15L1_**	-6.094674556	**-4.24260355**	-28.482143	18.8

**2015**	Forest	**350_15L1_**	-6.094674556	**-18.2426036**	-0.4821429	18
**2015**	Forest	**350_15L1_**	-6.094674556	**-6.24260355**	23.517857	16.7
**2015**	Forest	**3_15L2_**	3.905325444	**17.7573964**	-44.482143	17.8
**2015**	Forest	**3_15L2_**	3.905325444	**0.75739645**	-36.482143	17.8
**2015**	Forest	**3_15L2_**	3.905325444	**-17.2426036**	-23.482143	17
**2015**	Forest	**13_15L2_**	-6.094674556	**-23.2426036**	-42.482143	16.1
**2015**	Forest	**13_15L2_**	-6.094674556	**-13.2426036**	-26.482143	16.2
**2015**	Forest	**13_15L2_**	-6.094674556	**-19.2426036**	-8.4821429	15.9
**2015**	Forest	**15_15L2_**	-4.094674556	**-45.2426036**	-0.4821429	14.5
**2015**	Forest	**15_15L2_**	-4.094674556	**-35.2426036**	19.517857	14.8
**2015**	Forest	**15_15L2_**	-4.094674556	**-50.2426036**	50.517857	15.9
**2015**	Forest	**28_15L2_**	-2.094674556	**15.7573964**	-26.482143	15
**2015**	Forest	**28_15L2_**	-2.094674556	**20.7573964**	-9.4821429	15.3
**2015**	Forest	**28_15L2_**	-2.094674556	**18.7573964**	26.517857	15.5
**2015**	Forest	**31_15L2_**	1.905325444	**-17.2426036**	-10.482143	18.3
**2015**	Forest	**31_15L2_**	1.905325444	**-21.2426036**	-6.4821429	17.8
**2015**	Forest	**31_15L2_**	1.905325444	**-34.2426036**	60.517857	17
**2015**	Forest	**33_15L2_**	-7.094674556	**-17.2426036**	-25.482143	16
**2015**	Forest	**33_15L2_**	-7.094674556	**-14.2426036**	-20.482143	16.6
**2015**	Forest	**33_15L2_**	-7.094674556	**-15.2426036**	74.517857	16.6
**2015**	Forest	**34_15L2_**	1.905325444	**-18.2426036**	-47.482143	15.5
**2015**	Forest	**34_15L2_**	1.905325444	**-7.24260355**	-26.482143	15.8
**2015**	Forest	**34_15L2_**	1.905325444	**-30.2426036**	34.517857	17.2
**2015**	Forest	**36_15L2_**	-2.094674556	**2.75739645**	-21.482143	16.6
**2015**	Forest	**36_15L2_**	-2.094674556	**4.75739645**	-0.4821429	15.8
**2015**	Forest	**36_15L2_**	-2.094674556	**21.7573964**	16.517857	14.6
**2015**	Forest	**43_15L2_**	0.905325444	**-19.2426036**	-76.482143	15.5
**2015**	Forest	**43_15L2_**	0.905325444	**-23.2426036**	-65.482143	16.2
**2015**	Forest	**43_15L2_**	0.905325444	**-24.2426036**	-47.482143	16.1
**2015**	Forest	**43_15L2_**	0.905325444	**-10.2426036**	-41.482143	17
**2015**	Forest	**44_15L2_**	-2.094674556	**-27.2426036**	17.517857	15.3
**2015**	Forest	**44_15L2_**	-2.094674556	**-9.24260355**	20.517857	15.6
**2015**	Forest	**44_15L2_**	-2.094674556	**-31.2426036**	29.517857	15.4
**2015**	Park	**4_15b_**	-1.619883041	**-9.67961165**	3.872549	15.9
**2015**	Park	**4_15b_**	-1.619883041	**18.3203883**	3.872549	14.8
**2015**	Park	**4_15b_**	-1.619883041	**-19.6796117**	5.872549	14.1
**2015**	Park	**6_15b_**	-8.619883041	**31.3203883**	-27.127451	16.8
**2015**	Park	**6_15b_**	-8.619883041	**55.3203883**	63.872549	18.5
**2015**	Park	**6_15b_**	-8.619883041	**26.3203883**	83.872549	17.6
**2015**	Park	**7_15b_**	-3.619883041	**-35.6796117**	-61.127451	15
**2015**	Park	**7_15b_**	-3.619883041	**-28.6796117**	-59.127451	14.4
**2015**	Park	**7_15b_**	-3.619883041	**-39.6796117**	19.872549	14.9
**2015**	Park	**8_15b_**	-5.619883041	**-29.6796117**	-65.127451	12.2
**2015**	Park	**8_15b_**	-5.619883041	**-4.67961165**	-55.127451	13
**2015**	Park	**8_15b_**	-5.619883041	**-20.6796117**	26.872549	13
**2015**	Park	**11_15b_**	-6.619883041	**-10.6796117**	-29.127451	16.3
**2015**	Park	**11_15b_**	-6.619883041	**-3.67961165**	-8.127451	16.1
**2015**	Park	**11_15b_**	-6.619883041	**9.32038835**	-6.127451	15.3
**2015**	Park	**15_15b_**	3.380116959	**1.32038835**	-13.127451	13.8
**2015**	Park	**15_15b_**	3.380116959	**17.3203883**	22.872549	14.9
**2015**	Park	**15_15b_**	3.380116959	**-11.6796117**	53.872549	13.6
**2015**	Park	**17_15b_**	-1.619883041	**-3.67961165**	-49.127451	14.1
**2015**	Park	**17_15b_**	-1.619883041	**4.32038835**	-19.127451	16.3
**2015**	Park	**17_15b_**	-1.619883041	**23.3203883**	18.872549	15.6

**2015**	Park	**18_15b_**	3.380116959	**-31.6796117**	32.872549	10.4
**2015**	Park	**18_15b_**	3.380116959	**-36.6796117**	60.872549	10.5
**2015**	Park	**18_15b_**	3.380116959	**-57.6796117**	73.872549	11.8
**2015**	Park	**21_15b_**	2.380116959	**-10.6796117**	-61.127451	13.2
**2015**	Park	**21_15b_**	2.380116959	**7.32038835**	-20.127451	13.7
**2015**	Park	**21_15b_**	2.380116959	**2.32038835**	52.872549	14.4
**2015**	Park	**22_15b_**	-3.619883041	**-14.6796117**	-57.127451	14
**2015**	Park	**22_15b_**	-3.619883041	**3.32038835**	37.872549	14.9
**2015**	Park	**22_15b_**	-3.619883041	**-0.67961165**		14.4
**2015**	Park	**23_15b_**	5.380116959	**0.32038835**	49.872549	17.3
**2015**	Park	**23_15b_**	5.380116959	**-7.67961165**	54.872549	14.8
**2015**	Park	**23_15b_**	5.380116959	**-10.6796117**	126.87255	15.6
**2015**	Park	**26_15b_**	-0.619883041	**15.3203883**	-58.127451	15.2
**2015**	Park	**26_15b_**	-0.619883041	**3.32038835**	-38.127451	15.2
**2015**	Park	**26_15b_**	-0.619883041	**8.32038835**	20.872549	15.2
**2015**	Park	**28_15b_**	1.380116959	**-25.6796117**	-2.127451	15.4
**2015**	Park	**28_15b_**	1.380116959	**-27.6796117**	30.872549	12.7
**2015**	Park	**28_15b_**	1.380116959	**-20.6796117**		14.4
**2015**	Park	**28_15b_**	1.380116959	**-10.6796117**		13.4
**2015**	Park	**32_15b_**	1.380116959	**31.3203883**	-62.127451	16.6
**2015**	Park	**32_15b_**	1.380116959	**14.3203883**	4.872549	15.9
**2015**	Park	**32_15b_**	1.380116959	**12.3203883**	25.872549	15.4
**2015**	Park	**37_15b_**	4.380116959	**-32.6796117**	-56.127451	13.4
**2015**	Park	**37_15b_**	4.380116959	**-26.6796117**	-29.127451	14.6
**2015**	Park	**37_15b_**	4.380116959	**-10.6796117**	-12.127451	14.5
**2015**	Park	**40_15b_**	3.380116959	**-9.67961165**	-66.127451	13.2
**2015**	Park	**40_15b_**	3.380116959	**-10.6796117**	-53.127451	10.8
**2015**	Park	**40_15b_**	3.380116959	**-17.6796117**	0.872549	14.2
**2015**	Park	**49_15b_**	-1.619883041	**-22.6796117**	-2.127451	15.7
**2015**	Park	**49_15b_**	-1.619883041	**-21.6796117**	33.872549	15.7
**2015**	Park	**49_15b_**	-1.619883041	**-12.6796117**	62.872549	15.3
**2015**	Park	**52_15b_**	-3.619883041	**3.32038835**	-42.127451	17.2
**2015**	Park	**52_15b_**	-3.619883041	**3.32038835**	-12.127451	17.4
**2015**	Park	**52_15b_**	-3.619883041	**7.32038835**	31.872549	18.2
**2015**	Park	**53_15b_**	4.380116959	**-22.6796117**	21.872549	13.2
**2015**	Park	**53_15b_**	4.380116959	**-15.6796117**	94.872549	12.7
**2015**	Park	**53_15b_**	4.380116959	**-21.6796117**	112.87255	10.3
**2015**	Park	**54_15b_**	7.380116959	**-24.6796117**	-22.127451	14.9
**2015**	Park	**54_15b_**	7.380116959	**-35.6796117**	11.872549	15.8
**2015**	Park	**54_15b_**	7.380116959	**-31.6796117**	27.872549	14.1
**2015**	Park	**55_15b_**	-4.619883041	**0.32038835**	-34.127451	15.1
**2015**	Park	**55_15b_**	-4.619883041	**-9.67961165**	36.872549	14.1
**2015**	Park	**55_15b_**	-4.619883041	**-15.6796117**	59.872549	14.8
**2015**	Park	**59_15b_**	-4.619883041	**-7.67961165**	-52.127451	15.8
**2015**	Park	**59_15b_**	-4.619883041	**8.32038835**	-5.127451	15.4
**2015**	Park	**59_15b_**	-4.619883041	**9.32038835**	6.872549	16
**2015**	Park	**60_15b_**	1.380116959	**-2.67961165**	-38.127451	16.5
**2015**	Park	**60_15b_**	1.380116959	**1.32038835**	8.872549	16.5
**2015**	Park	**60_15b_**	1.380116959	**-10.6796117**	75.872549	17.2
**2015**	Park	**61_15b_**	0.380116959	**20.3203883**	-30.127451	19
**2015**	Park	**61_15b_**	0.380116959	**5.32038835**	-7.127451	18.6
**2015**	Park	**61_15b_**	0.380116959	**9.32038835**	-5.127451	17.9
**2015**	Park	**62_15b_**	5.380116959	**28.3203883**	-76.127451	18.7
**2015**	Park	**62_15b_**	5.380116959	**15.3203883**	-28.127451	17.1

**2015**	Park	**62_15b_**	5.380116959	**18.3203883**	-17.127451	17.9
**2015**	Park	**64_15b_**	0.380116959	**3.32038835**	36.872549	17.5
**2015**	Park	**64_15b_**	0.380116959	**-4.67961165**	37.872549	16.3
**2015**	Park	**64_15b_**	0.380116959	**3.32038835**	75.872549	16.6
**2015**	Park	**66_15b_**	5.380116959	**6.32038835**	-73.127451	16.9
**2015**	Park	**66_15b_**	5.380116959	**5.32038835**	-31.127451	17.6
**2015**	Park	**66_15b_**	5.380116959	**14.3203883**	72.872549	17.8
**2015**	Park	**72_15b_**	-2.619883041	**4.32038835**	-26.127451	15.9
**2015**	Park	**72_15b_**	-2.619883041	**-7.67961165**	2.872549	15.4
**2015**	Park	**72_15b_**	-2.619883041	**-16.6796117**	12.872549	15.3
**2015**	Park	**77_15b_**	0.380116959	**-1.67961165**	22.872549	13.7
**2015**	Park	**77_15b_**	0.380116959	**7.32038835**	66.872549	13.4
**2015**	Park	**77_15b_**	0.380116959	**-4.67961165**	88.872549	15.4
**2015**	Park	**78_15b_**	0.380116959	**-3.67961165**	-38.127451	15.3
**2015**	Park	**78_15b_**	0.380116959	**-18.6796117**	-1.127451	15.2
**2015**	Park	**78_15b_**	0.380116959	**-14.6796117**	38.872549	15.5
**2015**	Park	**80_15b_**	1.380116959	**-6.67961165**	-53.127451	14.9
**2015**	Park	**80_15b_**	1.380116959	**1.32038835**	-28.127451	15.4
**2015**	Park	**80_15b_**	1.380116959	**-15.6796117**	-9.127451	14.8
**2015**	Park	**81_15b_**	2.380116959	**-3.67961165**	-53.127451	13.5
**2015**	Park	**81_15b_**	2.380116959	**0.32038835**	-38.127451	14.8
**2015**	Park	**81_15b_**	2.380116959	**-14.6796117**	28.872549	14.7
**2015**	Park	**83_15b_**	1.380116959	**-33.6796117**	-16.127451	13.8
**2015**	Park	**83_15b_**	1.380116959	**-36.6796117**	16.872549	13
**2015**	Park	**83_15b_**	1.380116959	**-35.6796117**	40.872549	14.1
**2015**	Park	**90_15b_**	-3.619883041	**-26.6796117**	-35.127451	14.8
**2015**	Park	**90_15b_**	-3.619883041	**-42.6796117**	18.872549	12.3
**2015**	Park	**90_15b_**	-3.619883041	**-0.67961165**	32.872549	13.6
**2015**	Park	**91_15b_**	-2.619883041	**11.3203883**	17.872549	15.8
**2015**	Park	**91_15b_**	-2.619883041	**16.3203883**	61.872549	16.4
**2015**	Park	**91_15b_**	-2.619883041	**6.32038835**	62.872549	16.5
**2015**	Park	**92_15b_**	2.380116959	**31.3203883**	-39.127451	17.8
**2015**	Park	**92_15b_**	2.380116959	**46.3203883**	16.872549	17.1
**2015**	Park	**92_15b_**	2.380116959	**44.3203883**	33.872549	16
**2015**	Park	**93_15b_**	4.380116959	**-7.67961165**	29.872549	15.9
**2015**	Park	**93_15b_**	4.380116959	**-22.6796117**	33.872549	16.3
**2015**	Park	**93_15b_**	4.380116959	**-25.6796117**	45.872549	15.9
**2015**	Park	**96_15b_**	3.380116959	**16.3203883**	-17.127451	11.7
**2015**	Park	**96_15b_**	3.380116959	**-59.6796117**	45.872549	10.1
**2015**	Park	**97_15b_**	1.380116959	**32.3203883**	-92.127451	12.6
**2015**	Park	**97_15b_**	1.380116959	**2.32038835**	-87.127451	12.9
**2015**	Park	**97_15b_**	1.380116959	**0.32038835**	-71.127451	12.4
**2015**	Park	**99_15b_**	-1.619883041	**3.32038835**	-63.127451	16.2
**2015**	Park	**99_15b_**	-1.619883041	**18.3203883**	-35.127451	15.7
**2015**	Park	**99_15b_**	-1.619883041	**10.3203883**	32.872549	16.6
**2015**	Park	**100_15b_**	-5.619883041	**-15.6796117**	13.872549	16.6
**2015**	Park	**100_15b_**	-5.619883041	**16.3203883**	35.872549	14.5
**2015**	Park	**100_15b_**	-5.619883041	**-10.6796117**	60.872549	17.1
**2015**	Park	**101_15b_**	1.380116959	**9.32038835**	-42.127451	12.8
**2015**	Park	**101_15b_**	1.380116959	**-10.6796117**	-25.127451	13.9
**2015**	Park	**101_15b_**	1.380116959	**-5.67961165**	17.872549	14.6
**2015**	Park	**102_15b_**	-1.619883041		-39.127451	15.3
**2015**	Park	**102_15b_**	-1.619883041	**5.32038835**	2.872549	16.5
**2015**	Park	**102_15b_**	-1.619883041	**-7.67961165**	24.872549	17.5

**2015**	Park	**103_15b_**	0.380116959	**5.32038835**	-53.127451	16
**2015**	Park	**103_15b_**	0.380116959	**-4.67961165**	-9.127451	16.4
**2015**	Park	**103_15b_**	0.380116959	**-14.6796117**	105.87255	16.1
**2015**	Park	**104_15b_**	-6.619883041	**24.3203883**	-31.127451	14.3
**2015**	Park	**104_15b_**	-6.619883041	**24.3203883**	7.872549	14.2
**2015**	Park	**104_15b_**	-6.619883041	**34.3203883**	18.872549	15.1
**2015**	Park	**106_15b_**	-4.619883041	**31.3203883**	-46.127451	16
**2015**	Park	**106_15b_**	-4.619883041	**37.3203883**	-37.127451	16.2
**2015**	Park	**106_15b_**	-4.619883041	**7.32038835**	-8.127451	15.4
**2015**	Park	**107_15b_**	-4.619883041	**-5.67961165**	-45.127451	14.6
**2015**	Park	**107_15b_**	-4.619883041	**-12.6796117**	-10.127451	15.2
**2015**	Park	**107_15b_**	-4.619883041	**-8.67961165**	37.872549	15
**2015**	Park	**109_15b_**	3.380116959	**-5.67961165**	-14.127451	15.2
**2015**	Park	**109_15b_**	3.380116959	**-3.67961165**	27.872549	16.4
**2015**	Park	**109_15b_**	3.380116959	**-18.6796117**	81.872549	15.7
**2015**	Park	**110_15b_**	-2.619883041	**3.32038835**	-8.127451	13.9
**2015**	Park	**110_15b_**	-2.619883041	**-1.67961165**	-0.127451	14.3
**2015**	Park	**110_15b_**	-2.619883041	**5.32038835**	17.872549	13.3
**2015**	Park	**113_15b_**	-3.619883041	**14.3203883**	-65.127451	14.8
**2015**	Park	**113_15b_**	-3.619883041	**31.3203883**	-27.127451	15.4
**2015**	Park	**113_15b_**	-3.619883041	**5.32038835**	12.872549	15.7
**2015**	Park	**119_15b_**	1.380116959	**-5.67961165**	-53.127451	12.6
**2015**	Park	**119_15b_**	1.380116959	**2.32038835**	-6.127451	12.5
**2015**	Park	**119_15b_**	1.380116959	**22.3203883**	15.872549	12.7
**2015**	Park	**125_15b_**	-2.619883041	**8.32038835**	16.872549	15
**2015**	Park	**125_15b_**	-2.619883041	**26.3203883**	55.872549	18.3
**2015**	Park	**125_15b_**	-2.619883041	**7.32038835**	62.872549	17.2
**2015**	Park	**128_15b_**	-2.619883041	**4.32038835**	19.872549	15.9
**2015**	Park	**128_15b_**	-2.619883041	**7.32038835**	42.872549	16.2
**2015**	Park	**128_15b_**	-2.619883041	**24.3203883**	55.872549	14.3
**2015**	Park	**560_15b_**	7.380116959	**-10.6796117**	-70.127451	16.9
**2015**	Park	**560_15b_**	7.380116959	**0.32038835**	-63.127451	16.1
**2015**	Park	**560_15b_**	7.380116959	**-11.6796117**	-41.127451	16.9
**2015**	Park	**620_15b_**	3.380116959	**12.3203883**	-58.127451	14.3
**2015**	Park	**620_15b_**	3.380116959	**30.3203883**	-55.127451	16.3
**2015**	Park	**620_15b_**	3.380116959	**17.3203883**	-44.127451	16.9
**2015**	Park	**650_15b_**	2.380116959	**36.3203883**	-40.127451	16.1
**2015**	Park	**650_15b_**	2.380116959	**17.3203883**	-14.127451	15.7
**2015**	Park	**650_15b_**	2.380116959	**38.3203883**	-13.127451	15.5
**2015**	Park	**920_15b_**	5.380116959	**-13.6796117**	-25.127451	13.9
**2015**	Park	**920_15b_**	5.380116959	**-37.6796117**	76.872549	15.9
**2015**	Park	**920_15b_**	5.380116959	**-8.67961165**	91.872549	15.4
**2015**	Park	**3_15z_**	-2.619883041	**2.32038835**	-28.127451	16.4
**2015**	Park	**3_15z_**	-2.619883041	**-16.6796117**	-19.127451	15.4
**2015**	Park	**3_15z_**	-2.619883041	**-8.67961165**	5.872549	15.2
**2015**	Park	**11_15z_**	0.380116959	**6.32038835**	-60.127451	14.2
**2015**	Park	**11_15z_**	0.380116959	**6.32038835**	-22.127451	16
**2015**	Park	**11_15z_**	0.380116959	**-4.67961165**	-8.127451	14.4
**2015**	Park	**16_15z_**	-4.619883041	**39.3203883**	-72.127451	16.9
**2015**	Park	**16_15z_**	-4.619883041	**66.3203883**	-53.127451	16.2
**2015**	Park	**16_15z_**	-4.619883041	**31.3203883**	19.872549	16.5
**2015**	Park	**22_15z_**	-0.619883041	**16.3203883**	-57.127451	15.9
**2015**	Park	**22_15z_**	-0.619883041	**8.32038835**	26.872549	16.3
**2015**	Park	**22_15z_**	-0.619883041	**-1.67961165**	32.872549	15.2

**2015**	Park	**27_15z_**	-3.619883041	**-40.6796117**	-10.127451	13.5
**2015**	Park	**27_15z_**	-3.619883041	**-31.6796117**	0.872549	12.6
**2015**	Park	**27_15z_**	-3.619883041	**-36.6796117**	38.872549	12.6
**2015**	Park	**32_15z_**	-3.619883041	**14.3203883**	-3.127451	16.7
**2015**	Park	**32_15z_**	-3.619883041	**14.3203883**	54.872549	14.9
**2015**	Park	**32_15z_**	-3.619883041	**1.32038835**	88.872549	15.7
**2015**	Park	**37_15z_**	-2.619883041	**34.3203883**	-58.127451	14.1
**2015**	Park	**37_15z_**	-2.619883041	**26.3203883**	-53.127451	14.3
**2015**	Park	**37_15z_**	-2.619883041	**30.3203883**	-40.127451	13.5
**2015**	Park	**39_15z_**	-5.619883041	**13.3203883**	-20.127451	16.2
**2015**	Park	**39_15z_**	-5.619883041	**23.3203883**	-3.127451	15.9
**2015**	Park	**39_15z_**	-5.619883041	**24.3203883**	22.872549	15.7
**2015**	Park	**41_15z_**	-4.619883041	**-25.6796117**	29.872549	15.5
**2015**	Park	**41_15z_**	-4.619883041	**1.32038835**	45.872549	15.3
**2015**	Park	**41_15z_**	-4.619883041	**-18.6796117**	131.87255	15
**2015**	Park	**44_15z_**	-2.619883041	**8.32038835**	-45.127451	13.2
**2015**	Park	**44_15z_**	-2.619883041	**-13.6796117**	-35.127451	11.6
**2015**	Park	**44_15z_**	-2.619883041	**-21.6796117**	-1.127451	13.6
**2015**	Park	**59_15z_**	2.380116959	**-9.67961165**	-32.127451	15.2
**2015**	Park	**59_15z_**	2.380116959	**-14.6796117**	-30.127451	15.4
**2015**	Park	**59_15z_**	2.380116959	**-24.6796117**	17.872549	14.5
**2015**	Park	**60_15z_**	-3.619883041	**60.3203883**	-83.127451	16.6
**2015**	Park	**60_15z_**	-3.619883041	**38.3203883**	-50.127451	17.3
**2015**	Park	**60_15z_**	-3.619883041	**49.3203883**	-47.127451	16.2
**2016**	Forest	**5_16L1_**	-2.455696203	**-2.84810127**	-53.360759	17.9
**2016**	Forest	**5_16L1_**	-2.455696203	**19.1518987**	-12.360759	17.6
**2016**	Forest	**5_16L1_**	-2.455696203	**15.1518987**	8.6392405	17.5
**2016**	Forest	**7_16L1_**	-4.455696203	**6.15189873**	-63.360759	17.5
**2016**	Forest	**7_16L1_**	-4.455696203	**2.15189873**	-12.360759	18
**2016**	Forest	**7_16L1_**	-4.455696203	**0.15189873**	16.639241	17.4
**2016**	Forest	**17_16L1_**	0.544303797	**10.1518987**	-10.360759	16.5
**2016**	Forest	**22_16L1_**	-2.455696203	**22.1518987**	-73.360759	15.6
**2016**	Forest	**22_16L1_**	-2.455696203	**18.1518987**	-52.360759	15.8
**2016**	Forest	**22_16L1_**	-2.455696203	**10.1518987**	-49.360759	12.1
**2016**	Forest	**32_16L1_**	3.544303797	**-7.84810127**	-40.360759	17.9
**2016**	Forest	**32_16L1_**	3.544303797	**2.15189873**	37.639241	16.8
**2016**	Forest	**32_16L1_**	3.544303797	**-10.8481013**	50.639241	17.4
**2016**	Forest	**46_16L1_**	-1.455696203	**16.1518987**	-42.360759	17.3
**2016**	Forest	**46_16L1_**	-1.455696203	**9.15189873**	-38.360759	17.2
**2016**	Forest	**46_16L1_**	-1.455696203	**21.1518987**	-13.360759	18.3
**2016**	Forest	**53_16L1_**	17.5443038	**-33.8481013**	-16.360759	14.5
**2016**	Forest	**53_16L1_**	17.5443038	**-10.8481013**	1.6392405	14.4
**2016**	Forest	**53_16L1_**	17.5443038	**-6.84810127**	6.6392405	17
**2016**	Forest	**54_16L1_**	-2.455696203	**-1.84810127**	-69.360759	16.9
**2016**	Forest	**54_16L1_**	-2.455696203	**0.15189873**	-2.3607595	17.5
**2016**	Forest	**54_16L1_**	-2.455696203	**-0.84810127**	32.639241	18.7
**2016**	Forest	**60_16L1_**	1.544303797	**-9.84810127**	-14.360759	17.3
**2016**	Forest	**60_16L1_**	1.544303797	**-9.84810127**	2.6392405	16.1
**2016**	Forest	**60_16L1_**	1.544303797	**-9.84810127**	30.639241	16.9
**2016**	Forest	**63_16L1_**	-1.455696203	**11.1518987**	9.6392405	17
**2016**	Forest	**63_16L1_**	-1.455696203	**17.1518987**	27.639241	17.1
**2016**	Forest	**63_16L1_**	-1.455696203	**12.1518987**	41.639241	18
**2016**	Forest	**66_16L1_**	-1.455696203	**13.1518987**	-9.3607595	17.8
**2016**	Forest	**66_16L1_**	-1.455696203	**7.15189873**	9.6392405	18

**2016**	Forest	**66_16L1_**	-1.455696203	**1.15189873**	91.639241	16.2
**2016**	Forest	**73_16L1_**	1.544303797	**4.15189873**	-63.360759	18
**2016**	Forest	**73_16L1_**	1.544303797	**12.1518987**	-52.360759	17
**2016**	Forest	**73_16L1_**	1.544303797	**8.15189873**	30.639241	18
**2016**	Forest	**79_16L1_**	10.5443038	**-3.84810127**	62.639241	17.2
**2016**	Forest	**79_16L1_**	10.5443038	**-8.84810127**	77.639241	17.4
**2016**	Forest	**79_16L1_**	10.5443038	**-10.8481013**	118.63924	18.3
**2016**	Forest	**81_16L1_**	2.544303797	**-18.8481013**	31.639241	17.6
**2016**	Forest	**81_16L1_**	2.544303797	**-16.8481013**	44.639241	15.1
**2016**	Forest	**81_16L1_**	2.544303797	**-30.8481013**	108.63924	15.8
**2016**	Forest	**82_16L1_**	0.544303797	**26.1518987**	-87.360759	16.2
**2016**	Forest	**82_16L1_**	0.544303797	**13.1518987**	6.6392405	17.2
**2016**	Forest	**82_16L1_**	0.544303797	**24.1518987**	8.6392405	18.4
**2016**	Forest	**85_16L1_**	-2.455696203	**12.1518987**	-54.360759	17.5
**2016**	Forest	**85_16L1_**	-2.455696203	**4.15189873**	-48.360759	17.8
**2016**	Forest	**85_16L1_**	-2.455696203	**9.15189873**	-13.360759	16.8
**2016**	Forest	**95_16L1_**	-6.455696203	**12.1518987**	-64.360759	19
**2016**	Forest	**95_16L1_**	-6.455696203	**15.1518987**	-57.360759	17.4
**2016**	Forest	**95_16L1_**	-6.455696203	**-17.8481013**	-52.360759	17.9
**2016**	Forest	**103_16L1_**	1.544303797	**-7.84810127**	-6.3607595	17.3
**2016**	Forest	**103_16L1_**	1.544303797	**-6.84810127**	16.639241	17.7
**2016**	Forest	**103_16L1_**	1.544303797	**-14.8481013**	68.639241	17.3
**2016**	Forest	**104_16L1_**	-1.455696203	**36.1518987**	-67.360759	17.5
**2016**	Forest	**104_16L1_**	-1.455696203	**24.1518987**	-14.360759	16.3
**2016**	Forest	**104_16L1_**	-1.455696203	**7.15189873**	34.639241	15.6
**2016**	Forest	**105_16L1_**	-1.455696203	**-13.8481013**	4.6392405	17.2
**2016**	Forest	**105_16L1_**	-1.455696203	**-16.8481013**	9.6392405	18.4
**2016**	Forest	**105_16L1_**	-1.455696203	**-17.8481013**	103.63924	17.6
**2016**	Forest	**109_16L1_**	-6.455696203	**4.15189873**	-50.360759	14.8
**2016**	Forest	**109_16L1_**	-6.455696203	**6.15189873**	-11.360759	15.7
**2016**	Forest	**109_16L1_**	-6.455696203	**2.15189873**	2.6392405	16.5
**2016**	Forest	**121_16L1_**	1.544303797	**-6.84810127**	-18.360759	15.4
**2016**	Forest	**121_16L1_**	1.544303797	**4.15189873**	3.6392405	16.2
**2016**	Forest	**121_16L1_**	1.544303797	**-0.84810127**	13.639241	17.3
**2016**	Forest	**127_16L1_**	-3.455696203	**8.15189873**	-50.360759	16.4
**2016**	Forest	**127_16L1_**	-3.455696203	**26.1518987**	-48.360759	16.6
**2016**	Forest	**127_16L1_**	-3.455696203	**7.15189873**	17.639241	17.2
**2016**	Forest	**139_16L1_**	-0.455696203	**-18.8481013**	19.639241	13.7
**2016**	Forest	**139_16L1_**	-0.455696203	**-11.8481013**	27.639241	16.1
**2016**	Forest	**139_16L1_**	-0.455696203	**-1.84810127**	51.639241	18.9
**2016**	Forest	**146_16L1_**	0.544303797	**26.1518987**	-80.360759	16.8
**2016**	Forest	**146_16L1_**	0.544303797	**11.1518987**	-63.360759	17.9
**2016**	Forest	**146_16L1_**	0.544303797	**21.1518987**	11.639241	17.8
**2016**	Forest	**149_16L1_**	-0.455696203	**8.15189873**	11.639241	18.1
**2016**	Forest	**149_16L1_**	-0.455696203	**7.15189873**	16.639241	16.3
**2016**	Forest	**149_16L1_**	-0.455696203	**13.1518987**	25.639241	18
**2016**	Forest	**162_16L1_**	-2.455696203	**6.15189873**	-47.360759	17.3
**2016**	Forest	**162_16L1_**	-2.455696203	**9.15189873**	-34.360759	15.9
**2016**	Forest	**162_16L1_**	-2.455696203	**-12.8481013**	-14.360759	17.5
**2016**	Forest	**163_16L1_**	2.544303797	**8.15189873**	-30.360759	17
**2016**	Forest	**163_16L1_**	2.544303797	**7.15189873**	47.639241	18.5
**2016**	Forest	**163_16L1_**	2.544303797	**31.1518987**	51.639241	18.4
**2016**	Forest	**165_16L1_**	-4.455696203	**-10.8481013**	8.6392405	17.1
**2016**	Forest	**165_16L1_**	-4.455696203	**-3.84810127**	10.639241	15.6

**2016**	Forest	**165_16L1_**	-4.455696203	**0.15189873**	73.639241	15.4
**2016**	Forest	**168_16L1_**	-1.455696203	**14.1518987**	-56.360759	17.1
**2016**	Forest	**168_16L1_**	-1.455696203	**2.15189873**	-30.360759	16.2
**2016**	Forest	**168_16L1_**	-1.455696203	**18.1518987**	-5.3607595	18.1
**2016**	Forest	**170_16L1_**	-2.455696203	**-24.8481013**	27.639241	17.3
**2016**	Forest	**170_16L1_**	-2.455696203	**-23.8481013**	81.639241	15.8
**2016**	Forest	**170_16L1_**	-2.455696203	**-26.8481013**	90.639241	16
**2016**	Forest	**170_16L1_**	-2.455696203	**-27.8481013**	156.63924	16.8
**2016**	Forest	**170_16L1_**	-2.455696203	**-23.8481013**	156.63924	16.9
**2016**	Forest	**170_16L1_**	-2.455696203	**-22.8481013**	156.63924	16.3
**2016**	Forest	**171_16L1_**	0.544303797	**-2.84810127**	3.6392405	15.7
**2016**	Forest	**171_16L1_**	0.544303797	**-4.84810127**	40.639241	16.6
**2016**	Forest	**171_16L1_**	0.544303797	**6.15189873**	57.639241	16.6
**2016**	Forest	**185_16L1_**	1.544303797	**18.1518987**	-59.360759	15.7
**2016**	Forest	**185_16L1_**	1.544303797	**-9.84810127**	-50.360759	16.4
**2016**	Forest	**185_16L1_**	1.544303797	**5.15189873**	-6.3607595	17.1
**2016**	Forest	**187_16L1_**	1.544303797	**-2.84810127**	-48.360759	16.7
**2016**	Forest	**187_16L1_**	1.544303797	**11.1518987**	-33.360759	16.5
**2016**	Forest	**187_16L1_**	1.544303797	**5.15189873**	25.639241	16.9
**2016**	Forest	**311_16L1_**	-4.455696203	**-0.84810127**	-33.360759	14.3
**2016**	Forest	**311_16L1_**	-4.455696203	**15.1518987**	-32.360759	15.6
**2016**	Forest	**311_16L1_**	-4.455696203	**9.15189873**	-26.360759	17.2
**2016**	Forest	**311_16L1_**	-4.455696203	**12.1518987**	-10.360759	14.6
**2016**	Forest	**311_16L1_**	-4.455696203	**20.1518987**	48.639241	15.5
**2016**	Forest	**311_16L1_**	-4.455696203	**-1.84810127**	110.63924	15.8
**2016**	Forest	**311_16L1_**	-4.455696203	**7.15189873**	111.63924	15.2
**2016**	Forest	**317_16L1_**	18.5443038	**-28.8481013**	0.6392405	14
**2016**	Forest	**317_16L1_**	18.5443038	**-18.8481013**	2.6392405	15.5
**2016**	Forest	**317_16L1_**	18.5443038	**-17.8481013**	21.639241	14.9
**2016**	Forest	**320_16L1_**	3.544303797	**7.15189873**	-35.360759	18.2
**2016**	Forest	**320_16L1_**	3.544303797	**8.15189873**	-26.360759	17.1
**2016**	Forest	**320_16L1_**	3.544303797	**21.1518987**	-19.360759	18.5
**2016**	Forest	**324_16L1_**	-2.455696203	**6.15189873**	-72.360759	17.5
**2016**	Forest	**324_16L1_**	-2.455696203	**10.1518987**	-64.360759	17.3
**2016**	Forest	**324_16L1_**	-2.455696203	**2.15189873**	42.639241	17.3
**2016**	Forest	**341_16L1_**	0.544303797	**-1.84810127**	-53.360759	16
**2016**	Forest	**341_16L1_**	0.544303797	**-1.84810127**	-32.360759	16.1
**2016**	Forest	**341_16L1_**	0.544303797	**-12.8481013**	-31.360759	17.3
**2016**	Forest	**341_16L1_**	0.544303797	**-1.84810127**	-29.360759	16.8
**2016**	Forest	**341_16L1_**	0.544303797	**-12.8481013**	-20.360759	16.3
**2016**	Forest	**341_16L1_**	0.544303797	**10.1518987**	22.639241	16.1
**2016**	Forest	**342_16L1_**	3.544303797	**-0.84810127**	-48.360759	12.4
**2016**	Forest	**342_16L1_**	3.544303797	**-4.84810127**	-45.360759	10.7
**2016**	Forest	**342_16L1_**	3.544303797	**-24.8481013**	20.639241	12.6
**2016**	Forest	**344_16L1_**	3.544303797	**2.15189873**	-73.360759	15.6
**2016**	Forest	**344_16L1_**	3.544303797	**8.15189873**	-68.360759	15.3
**2016**	Forest	**344_16L1_**	3.544303797	**2.15189873**	10.639241	15.2
**2016**	Forest	**11_16L2_**	-2.455696203	**-13.8481013**	-73.360759	16
**2016**	Forest	**11_16L2_**	-2.455696203	**-11.8481013**	1.6392405	16.5
**2016**	Forest	**11_16L2_**	-2.455696203	**-6.84810127**	11.639241	15.2
**2016**	Forest	**29_16L2_**	-4.455696203	**16.1518987**	-63.360759	13.4
**2016**	Forest	**29_16L2_**	-4.455696203	**5.15189873**	-58.360759	12.7
**2016**	Forest	**29_16L2_**	-4.455696203	**2.15189873**	-18.360759	14.5
**2016**	Forest	**29_16L2_**	-4.455696203	**9.15189873**	26.639241	16.2

**2016**	Forest	**29_16L2_**	-4.455696203	**2.15189873**	40.639241	15
**2016**	Forest	**29_16L2_**	-4.455696203	**6.15189873**	54.639241	14.8
**2016**	Forest	**32_16L2_**	-3.455696203	**-40.8481013**	-33.360759	14.6
**2016**	Forest	**32_16L2_**	-3.455696203	**-20.8481013**	-28.360759	13.1
**2016**	Forest	**32_16L2_**	-3.455696203	**-28.8481013**	-25.360759	14.3
**2016**	Forest	**32_16L2_**	-3.455696203	**-28.8481013**	-16.360759	14
**2016**	Forest	**32_16L2_**	-3.455696203	**-39.8481013**	16.639241	15.5
**2016**	Forest	**32_16L2_**	-3.455696203	**-17.8481013**	16.639241	15.1
**2016**	Forest	**33_16L2_**	2.544303797	**4.15189873**	-54.360759	16.9
**2016**	Forest	**33_16L2_**	2.544303797	**3.15189873**	-43.360759	17.1
**2016**	Forest	**33_16L2_**	2.544303797	**-7.84810127**	18.639241	16.8
**2016**	Forest	**36_16L2_**	-5.455696203	**-11.8481013**	-26.360759	17.5
**2016**	Forest	**36_16L2_**	-5.455696203	**-9.84810127**	9.6392405	14.6
**2016**	Forest	**36_16L2_**	-5.455696203	**-2.84810127**	9.6392405	15.5
**2016**	Forest	**41_16L2_**	11.5443038	**-9.84810127**	-30.360759	13.7
**2016**	Forest	**41_16L2_**	11.5443038	**-0.84810127**	4.6392405	16.3
**2016**	Forest	**41_16L2_**	11.5443038	**2.15189873**	25.639241	16.1
**2016**	Forest	**44_16L2_**	-3.455696203	**10.1518987**	45.639241	16.7
**2016**	Forest	**44_16L2_**	-3.455696203	**5.15189873**	52.639241	15.9
**2016**	Forest	**44_16L2_**	-3.455696203	**-3.84810127**	72.639241	15.9
**2016**	Park	**2_16b_**	-1.106280193	**-21.4117647**	-58.823529	16.6
**2016**	Park	**2_16b_**	-1.106280193	**-26.4117647**	-52.823529	14.8
**2016**	Park	**2_16b_**	-1.106280193	**-16.4117647**	-41.823529	16
**2016**	Park	**2_16b_**	-1.106280193	**-21.4117647**	-35.823529	17
**2016**	Park	**2_16b_**	-1.106280193	**-16.4117647**	-27.823529	16.4
**2016**	Park	**2_16b_**	-1.106280193	**-24.4117647**	-15.823529	14.9
**2016**	Park	**5_16b_**	-4.106280193	**27.5882353**	-75.823529	13.8
**2016**	Park	**5_16b_**	-4.106280193	**18.5882353**	-64.823529	13.7
**2016**	Park	**5_16b_**	-4.106280193	**23.5882353**	-31.823529	13.6
**2016**	Park	**8_16b_**	3.893719807	**-28.4117647**	20.176471	14.5
**2016**	Park	**8_16b_**	3.893719807	**-51.4117647**	61.176471	12.2
**2016**	Park	**11_16b_**	-3.106280193	**-6.41176471**	-96.823529	13.7
**2016**	Park	**11_16b_**	-3.106280193	**0.58823529**	-16.823529	12.5
**2016**	Park	**11_16b_**	-3.106280193	**10.5882353**	23.176471	12.8
**2016**	Park	**11_16b_**	-3.106280193	**10.5882353**	140.17647	13.4
**2016**	Park	**14_16b_**	6.893719807	**-18.4117647**	-45.823529	13.6
**2016**	Park	**18_16b_**	0.893719807		-53.823529	13.7
**2016**	Park	**18_16b_**	0.893719807	**-32.4117647**	20.176471	12.4
**2016**	Park	**18_16b_**	0.893719807	**-42.4117647**	23.176471	14.1
**2016**	Park	**21_16b_**	-0.106280193	**23.5882353**	-82.823529	16
**2016**	Park	**21_16b_**	-0.106280193	**47.5882353**	-65.823529	15.2
**2016**	Park	**21_16b_**	-0.106280193	**14.5882353**	-54.823529	15.7
**2016**	Park	**22_16b_**	-0.106280193	**41.5882353**	-46.823529	16.9
**2016**	Park	**22_16b_**	-0.106280193	**40.5882353**	-40.823529	17
**2016**	Park	**22_16b_**	-0.106280193	**20.5882353**	-21.823529	14.5
**2016**	Park	**22_16b_**	-0.106280193	**34.5882353**	-7.8235294	16.7
**2016**	Park	**22_16b_**	-0.106280193	**31.5882353**	9.1764706	16
**2016**	Park	**22_16b_**	-0.106280193	**21.5882353**	30.176471	17.6
**2016**	Park	**23_16b_**	-0.106280193	**-34.4117647**	60.176471	12.7
**2016**	Park	**23_16b_**	-0.106280193	**-8.41176471**	65.176471	7.4
**2016**	Park	**26_16b_**	4.893719807	**-3.41176471**	56.176471	15.3
**2016**	Park	**26_16b_**	4.893719807	**3.58823529**	57.176471	16.6
**2016**	Park	**26_16b_**	4.893719807	**3.58823529**	98.176471	16.5
**2016**	Park	**28_16b_**	1.893719807	**20.5882353**	-3.8235294	19.2

**2016**	Park	**28_16b_**	1.893719807	**16.5882353**	36.176471	18
**2016**	Park	**28_16b_**	1.893719807	**21.5882353**	101.17647	18.6
**2016**	Park	**31_16b_**	0.893719807	**-53.4117647**	6.1764706	13.1
**2016**	Park	**31_16b_**	0.893719807	**-30.4117647**	38.176471	15.1
**2016**	Park	**32_16b_**	2.893719807	**1.58823529**	-53.823529	12.4
**2016**	Park	**32_16b_**	2.893719807	**-8.41176471**	-38.823529	13.1
**2016**	Park	**32_16b_**	2.893719807	**-7.41176471**	13.176471	11.9
**2016**	Park	**34_16b_**	2.893719807	**-46.4117647**	6.1764706	14.3
**2016**	Park	**34_16b_**	2.893719807	**-40.4117647**	85.176471	12.2
**2016**	Park	**34_16b_**	2.893719807	**-43.4117647**	140.17647	11.7
**2016**	Park	**41_16b_**	7.893719807	**-54.4117647**	-10.823529	14.3
**2016**	Park	**41_16b_**	7.893719807	**-43.4117647**	71.176471	15.7
**2016**	Park	**41_16b_**	7.893719807	**-38.4117647**	102.17647	14.4
**2016**	Park	**42_16b_**	-0.106280193	**16.5882353**	-81.823529	11.3
**2016**	Park	**42_16b_**	-0.106280193	**17.5882353**	-73.823529	15.3
**2016**	Park	**42_16b_**	-0.106280193	**5.58823529**	-20.823529	14.6
**2016**	Park	**46_16b_**	0.893719807	**3.58823529**	-35.823529	16.4
**2016**	Park	**46_16b_**	0.893719807	**-3.41176471**	-22.823529	15.3
**2016**	Park	**46_16b_**	0.893719807	**3.58823529**	27.176471	16.3
**2016**	Park	**47_16b_**	-7.106280193	**-11.4117647**	-99.823529	14.9
**2016**	Park	**47_16b_**	-7.106280193	**-14.4117647**	-13.823529	11.7
**2016**	Park	**47_16b_**	-7.106280193	**-2.41176471**	-0.8235294	14.4
**2016**	Park	**50_16b_**	-4.106280193	**15.5882353**	-57.823529	15.4
**2016**	Park	**50_16b_**	-4.106280193	**9.58823529**	-31.823529	15.3
**2016**	Park	**50_16b_**	-4.106280193	**7.58823529**	2.1764706	15.3
**2016**	Park	**52_16b_**	-6.106280193	**10.5882353**	-41.823529	14.3
**2016**	Park	**52_16b_**	-6.106280193	**11.5882353**	-27.823529	15.2
**2016**	Park	**52_16b_**	-6.106280193	**5.58823529**	91.176471	15.1
**2016**	Park	**53_16b_**	2.893719807	**-27.4117647**	15.176471	14.7
**2016**	Park	**53_16b_**	2.893719807	**-27.4117647**	99.176471	14
**2016**	Park	**53_16b_**	2.893719807	**-24.4117647**	101.17647	13.5
**2016**	Park	**58_16b_**	-6.106280193	**11.5882353**	-91.823529	14.6
**2016**	Park	**58_16b_**	-6.106280193	**10.5882353**	-52.823529	13.7
**2016**	Park	**58_16b_**	-6.106280193	**10.5882353**	-45.823529	14.5
**2016**	Park	**60_16b_**	-3.106280193	**29.5882353**	-66.823529	16.1
**2016**	Park	**60_16b_**	-3.106280193	**-0.41176471**	-30.823529	16.5
**2016**	Park	**60_16b_**	-3.106280193	**10.5882353**	-20.823529	16.4
**2016**	Park	**62_16b_**	1.893719807	**22.5882353**	-43.823529	17.3
**2016**	Park	**62_16b_**	1.893719807	**32.5882353**	3.1764706	18.4
**2016**	Park	**62_16b_**	1.893719807	**22.5882353**	46.176471	18.7
**2016**	Park	**63_16b_**	1.893719807	**36.5882353**	-9.8235294	17.9
**2016**	Park	**63_16b_**	1.893719807	**20.5882353**	5.1764706	17.7
**2016**	Park	**63_16b_**	1.893719807	**31.5882353**	62.176471	17.3
**2016**	Park	**64_16b_**	4.893719807	**10.5882353**	-23.823529	20.5
**2016**	Park	**64_16b_**	4.893719807	**15.5882353**	38.176471	17.6
**2016**	Park	**64_16b_**	4.893719807	**-1.41176471**	81.176471	18.3
**2016**	Park	**66_16b_**	2.893719807	**4.58823529**	-53.823529	14.8
**2016**	Park	**66_16b_**	2.893719807	**3.58823529**	-45.823529	15.1
**2016**	Park	**66_16b_**	2.893719807	**-3.41176471**	7.1764706	15.7
**2016**	Park	**67_16b_**	3.893719807	**-11.4117647**	-45.823529	15.3
**2016**	Park	**67_16b_**	3.893719807	**-0.41176471**	-45.823529	15.8
**2016**	Park	**67_16b_**	3.893719807	**-3.41176471**	-37.823529	15.4
**2016**	Park	**70_16b_**	3.893719807		-46.823529	15.2
**2016**	Park	**70_16b_**	3.893719807	**-4.41176471**	-44.823529	16

**2016**	Park	**70_16b_**	3.893719807	**-15.4117647**	-33.823529	15.9
**2016**	Park	**73_16b_**	-2.106280193	**24.5882353**	-62.823529	15.6
**2016**	Park	**73_16b_**	-2.106280193	**21.5882353**	-33.823529	15.2
**2016**	Park	**73_16b_**	-2.106280193	**37.5882353**	-13.823529	15.7
**2016**	Park	**79_16b_**	-0.106280193	**24.5882353**	-42.823529	15.7
**2016**	Park	**79_16b_**	-0.106280193	**-1.41176471**	-31.823529	17
**2016**	Park	**79_16b_**	-0.106280193	**2.58823529**	-29.823529	15.9
**2016**	Park	**81_16b_**	1.893719807	**-25.4117647**	57.176471	15.7
**2016**	Park	**81_16b_**	1.893719807	**-7.41176471**	65.176471	16.4
**2016**	Park	**81_16b_**	1.893719807	**-27.4117647**	77.176471	16.3
**2016**	Park	**84_16b_**	1.893719807	**-45.4117647**	-18.823529	11.7
**2016**	Park	**84_16b_**	1.893719807	**-58.4117647**	24.176471	10
**2016**	Park	**84_16b_**	1.893719807	**-48.4117647**	140.17647	11.5
**2016**	Park	**87_16b_**	16.89371981	**-16.4117647**	5.1764706	15.8
**2016**	Park	**87_16b_**	16.89371981	**-0.41176471**	13.176471	17.7
**2016**	Park	**87_16b_**	16.89371981	**-14.4117647**	84.176471	12.3
**2016**	Park	**89_16b_**	2.893719807	**14.5882353**	48.176471	17.5
**2016**	Park	**89_16b_**	2.893719807	**2.58823529**	50.176471	16.8
**2016**	Park	**89_16b_**	2.893719807	**10.5882353**	101.17647	17.4
**2016**	Park	**90_16b_**	-0.106280193	**22.5882353**	-58.823529	17.1
**2016**	Park	**90_16b_**	-0.106280193	**15.5882353**	-43.823529	16.2
**2016**	Park	**90_16b_**	-0.106280193	**20.5882353**	-30.823529	18
**2016**	Park	**92_16b_**	1.893719807	**-30.4117647**	86.176471	16.5
**2016**	Park	**92_16b_**	1.893719807	**-33.4117647**	116.17647	15.2
**2016**	Park	**92_16b_**	1.893719807	**-22.4117647**	129.17647	15.7
**2016**	Park	**93_16b_**	3.893719807	**12.5882353**	-0.8235294	17
**2016**	Park	**93_16b_**	3.893719807	**31.5882353**	60.176471	18.5
**2016**	Park	**93_16b_**	3.893719807	**28.5882353**	77.176471	18.5
**2016**	Park	**98_16b_**	-3.106280193	**8.58823529**	14.176471	15.1
**2016**	Park	**98_16b_**	-3.106280193	**9.58823529**	27.176471	16.5
**2016**	Park	**98_16b_**	-3.106280193	**8.58823529**	39.176471	16.4
**2016**	Park	**99_16b_**	-4.106280193	**13.5882353**	-14.823529	12.8
**2016**	Park	**99_16b_**	-4.106280193	**13.5882353**	8.1764706	11.9
**2016**	Park	**99_16b_**	-4.106280193	**7.58823529**	26.176471	12.7
**2016**	Park	**100_16b_**	-4.106280193	**-22.4117647**	-7.8235294	13.4
**2016**	Park	**100_16b_**	-4.106280193	**-33.4117647**	15.176471	13.3
**2016**	Park	**100_16b_**	-4.106280193	**-24.4117647**	37.176471	12.3
**2016**	Park	**101_16b_**	-4.106280193	**-19.4117647**	-4.8235294	14
**2016**	Park	**101_16b_**	-4.106280193	**-18.4117647**	-3.8235294	12
**2016**	Park	**101_16b_**	-4.106280193	**-24.4117647**	47.176471	13.5
**2016**	Park	**105_16b_**	-4.106280193	**2.58823529**	-70.823529	13.3
**2016**	Park	**105_16b_**	-4.106280193	**-7.41176471**	-51.823529	13.3
**2016**	Park	**105_16b_**	-4.106280193	**-21.4117647**	-35.823529	12.3
**2016**	Park	**107_16b_**	-1.106280193	**-65.4117647**	-86.823529	8.8
**2016**	Park	**107_16b_**	-1.106280193	**-28.4117647**	-80.823529	12.5
**2016**	Park	**107_16b_**	-1.106280193	**-25.4117647**	-64.823529	10.9
**2016**	Park	**107_16b_**	-1.106280193	**-27.4117647**	-41.823529	11.2
**2016**	Park	**107_16b_**	-1.106280193	**-33.4117647**	-11.823529	12.1
**2016**	Park	**107_16b_**	-1.106280193	**-38.4117647**	41.176471	10.1
**2016**	Park	**109_16b_**	1.893719807	**22.5882353**	40.176471	16
**2016**	Park	**109_16b_**	1.893719807	**-4.41176471**	41.176471	15.5
**2016**	Park	**109_16b_**	1.893719807	**-7.41176471**	69.176471	15.8
**2016**	Park	**110_16b_**	-0.106280193	**12.5882353**	-9.8235294	17.8
**2016**	Park	**110_16b_**	-0.106280193	**0.58823529**	15.176471	17.8

**2016**	Park	**110_16b_**	-0.106280193		29.176471	20.3
**2016**	Park	**114_16b_**	-1.106280193	**18.5882353**	-88.823529	13.8
**2016**	Park	**114_16b_**	-1.106280193	**3.58823529**	44.176471	13.8
**2016**	Park	**114_16b_**	-1.106280193	**48.5882353**	54.176471	13.7
**2016**	Park	**116_16b_**	-1.106280193	**43.5882353**	-44.823529	13.6
**2016**	Park	**116_16b_**	-1.106280193	**20.5882353**	-34.823529	13.6
**2016**	Park	**116_16b_**	-1.106280193	**46.5882353**	-25.823529	13.6
**2016**	Park	**120_16b_**	-1.106280193	**9.58823529**	-83.823529	11
**2016**	Park	**120_16b_**	-1.106280193	**14.5882353**	-70.823529	14.4
**2016**	Park	**120_16b_**	-1.106280193	**-9.41176471**	-4.8235294	15.3
**2016**	Park	**121_16b_**	0.893719807	**-8.41176471**	-40.823529	12.9
**2016**	Park	**121_16b_**	0.893719807	**-21.4117647**	-38.823529	12.5
**2016**	Park	**121_16b_**	0.893719807	**-1.41176471**	47.176471	13
**2016**	Park	**122_16b_**	-1.106280193	**8.58823529**	-18.823529	13.7
**2016**	Park	**122_16b_**	-1.106280193	**-20.4117647**	15.176471	13.3
**2016**	Park	**122_16b_**	-1.106280193	**-12.4117647**	61.176471	13.1
**2016**	Park	**125_16b_**	1.893719807	**29.5882353**	-42.823529	16.6
**2016**	Park	**125_16b_**	1.893719807	**27.5882353**	-7.8235294	15.6
**2016**	Park	**125_16b_**	1.893719807	**52.5882353**	59.176471	16.4
**2016**	Park	**620_16b_**	-8.106280193	**22.5882353**		16.1
**2016**	Park	**620_16b_**	-8.106280193	**34.5882353**		16.1
**2016**	Park	**620_16b_**	-8.106280193	**34.5882353**		13.3
**2016**	Park	**920_16b_**	-0.106280193	**21.5882353**	-81.823529	16.1
**2016**	Park	**920_16b_**	-0.106280193	**35.5882353**	-44.823529	17.3
**2016**	Park	**920_16b_**	-0.106280193	**32.5882353**	106.17647	17.3
**2016**	Park	**3_16z_**	-6.106280193	**-4.41176471**	-17.823529	15.9
**2016**	Park	**3_16z_**	-6.106280193	**-9.41176471**	16.176471	15.9
**2016**	Park	**3_16z_**	-6.106280193	**-15.4117647**	67.176471	16.4
**2016**	Park	**11_16z**	0.893719807	**3.58823529**	0.1764706	16.2
**2016**	Park	**11_16z**	0.893719807	**-14.4117647**	4.1764706	15.1
**2016**	Park	**11_16z**	0.893719807	**-12.4117647**	11.176471	15.2
**2016**	Park	**16_16z_**	-7.106280193	**-14.4117647**	-31.823529	14.5
**2016**	Park	**22_16z_**	-6.106280193	**-13.4117647**	-78.823529	14.6
**2016**	Park	**22_16z_**	-6.106280193	**-8.41176471**	-58.823529	15.2
**2016**	Park	**22_16z_**	-6.106280193	**-17.4117647**	-20.823529	15
**2016**	Park	**27_16z_**	5.893719807	**34.5882353**	-86.823529	14.4
**2016**	Park	**27_16z_**	5.893719807	**18.5882353**	-74.823529	14.9
**2016**	Park	**27_16z_**	5.893719807	**26.5882353**	-68.823529	15.1
**2016**	Park	**32_16z_**	0.893719807	**-20.4117647**	-19.823529	16.1
**2016**	Park	**32_16z_**	0.893719807	**6.58823529**	-7.8235294	16
**2016**	Park	**32_16z_**	0.893719807	**-14.4117647**	60.176471	15.5
**2016**	Park	**35_16z_**	1.893719807	**25.5882353**	-44.823529	15.7
**2016**	Park	**35_16z_**	1.893719807	**31.5882353**	37.176471	16.4
**2016**	Park	**35_16z_**	1.893719807	**17.5882353**	53.176471	16.6
**2016**	Park	**45_16z_**	1.893719807	**10.5882353**	-0.8235294	12.9
**2016**	Park	**45_16z_**	1.893719807	**21.5882353**	-0.8235294	16
**2016**	Park	**45_16z_**	1.893719807	**17.5882353**	50.176471	14.1
**2016**	Park	**50_16z_**	0.893719807	**-15.4117647**	-7.8235294	11.8
**2016**	Park	**50_16z_**	0.893719807	**-11.4117647**	3.1764706	15.1
**2016**	Park	**50_16z_**	0.893719807	**-24.4117647**	58.176471	10.4
**2016**	Park	**53_16z_**	-1.106280193	**-18.4117647**	-39.823529	12.9
**2016**	Park	**53_16z_**	-1.106280193	**-2.41176471**	-0.8235294	15.3
**2016**	Park	**53_16z_**	-1.106280193	**-28.4117647**	121.17647	10.7
**2016**	Park	**55_16z_**	0.893719807	**2.58823529**	-18.823529	15.8

**2016**	Park	**55_16z_**	0.893719807	**8.58823529**	-3.8235294	16.2
**2016**	Park	**55_16z_**	0.893719807	**7.58823529**	63.176471	16.3
**2016**	Park	**57_16z_**	-1.106280193	**-5.41176471**	-44.823529	14.8
**2016**	Park	**57_16z_**	-1.106280193	**-6.41176471**	-31.823529	14.5
**2016**	Park	**57_16z_**	-1.106280193	**-12.4117647**	10.176471	15.6
**2016**	Park	**58_16z_**	1.893719807	**22.5882353**	14.176471	15.7
**2016**	Park	**58_16z_**	1.893719807	**17.5882353**	44.176471	16.6
**2016**	Park	**58_16z_**	1.893719807	**16.5882353**	92.176471	14.7
**2016**	Park	**60_16z_**	-8.106280193	**-20.4117647**	-68.823529	12.1
**2016**	Park	**60_16z_**	-8.106280193	**6.58823529**	-14.823529	12.5
**2016**	Park	**60_16z_**	-8.106280193	**-23.4117647**	2.1764706	11.9
